# New Sulfenate Sources for Double Pallado-Catalyzed Cross-Coupling Reaction: Application in Symmetrical Biarylsulfoxide Synthesis, and Evidence of TADF Properties

**DOI:** 10.3390/molecules29204809

**Published:** 2024-10-11

**Authors:** Valentin Magné, Iulia Cretoiu, Sonia Mallet-Ladeira, Eddy Maerten, David Madec

**Affiliations:** 1 Laboratoire Hétérochimie Fondamentale et Appliquée (UMR 5069), Université de Toulouse, CNRS, 118 Route de Narbonne, CEDEX 09, 31062 Toulouse, France; valentin.magne@univ-tlse3.fr (V.M.); iulia.cretoiu@univ-tlse3.fr (I.C.); eddy.maerten@univ-tlse3.fr (E.M.); 2 Institut de Chimie de Toulouse (UAR 2599), 118 Route de Narbonne, CEDEX 09, 31062 Toulouse, France; sonia.ladeira@univ-tlse3.fr

**Keywords:** sulfoxides, sulfenate, palladium, TADF

## Abstract

Tetrahydro-4*H*-thiopyran-4-one 1-oxide **1** and sulfinyl-di-*tert*-butylpropionate **2** were reported as sources of bis-sulfenate anion and applied in a double pallado-catalyzed cross-coupling reaction for the synthesis of symmetrical biarylsulfoxides, tolerating a large array of electronic properties and bulkiness. The photophysical properties of a biarylsulfoxide have been explored, demonstrating an unreported TADF phenomenon on sulfoxide-containing scaffolds.

## 1. Introduction

**Figure 1:**
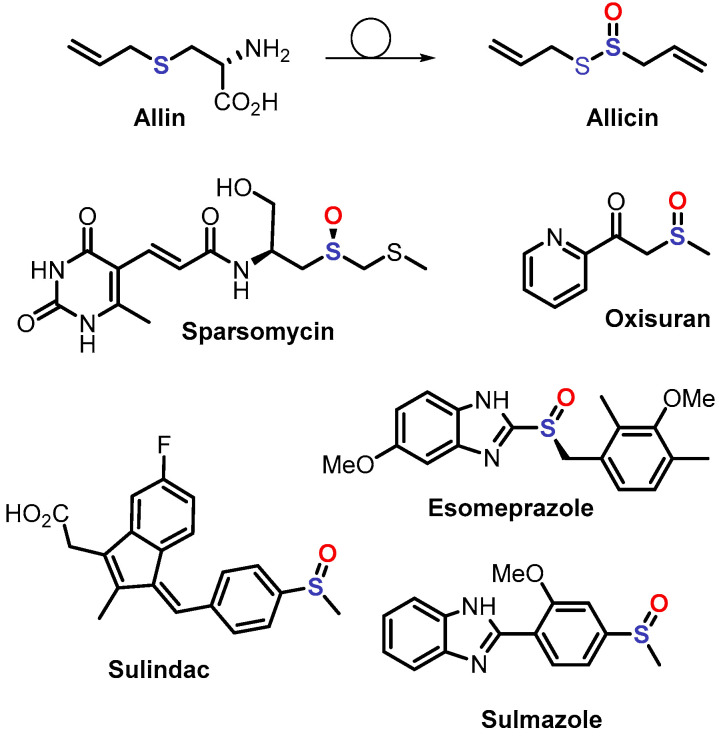
Examples of bioactive sulfoxides or precursors of sulfoxides.

Sulfoxides are ubiquitous scaffolds in organic chemistry. The simplest of the sulfoxides, dimethyl sulfoxide (DMSO) is a solvent of choice for synthetic chemists and biochemists because of its physicochemical properties and biocompatibility [1,2,3,4]. Natural sulfoxides or precursors of sulfoxides are involved in various biological processes [5,6] such as alliin bio-transformed into allicin, an antifungal agent, Sparsomycin with antibiotic activities or Oxisuran with immunosuppressive properties. Aromatic sulfoxides are often used in synthetic therapeutic chemistry, with, for example, Sulindac with anti-inflammatory activity, Sulmazole in the treatment of cardiovascular anomalies or Esomeprazole involved in proton pump inhibition (Figure 1). Aromatic sulfoxides are also important in coordination chemistry for the stabilization of organometallic complexes and their applications in catalysis [7,8,9,10], for the stabilization of low-valent species of main group elements [11,12,13,14,15], and in materials science [16,17]. Aromatic sulfoxides are mainly prepared by oxidation of the corresponding thioethers, or by nucleophilic substitution reaction of sulfinate amides or esters [18]. Each of these two methods suffers from limitations such as the formation of over-oxidation products, or the incompatibility of nucleophilic reagents with different functional groups.

**Scheme 1:**
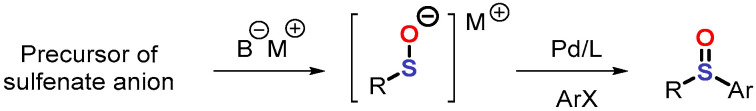
Palladium-catalyzed arylation of sulfenate anions.

Recently, a new synthetic strategy involving a pallado-catalyzed cross coupling with sulfenate anions was developed [19,20,21,22,23]. This approach allows perfect control of the oxidation state of the sulfoxide synthesized, as well as good compatibility with a wide range of functional groups (Scheme 1).

**Figure 2:**
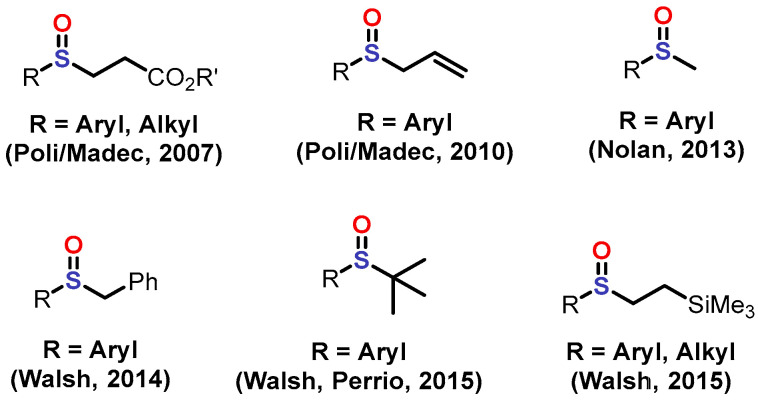
Sources of sulfenate anions in palladium-catalyzed arylation [19,24,25,26,27,28,29,30,31,32,33,34].

This pallado-catalyzed coupling reaction allows the synthesis of various unsymmetrical aromatic sulfoxides from different sulfenate anion precursors (Figure 2) [24,25,26,27,28,29,30,31,32,33,34], and enantioselective versions were also developed [25,34,35,36].

In contrast, the use of sulfenate ions for the synthesis of symmetrical sulfoxides by palladium-catalyzed cross-coupling reaction remains sporadic [28,29,32]. Interested in the preparation of various symmetrical sulfoxides, in particular with highly hindered groups, we decided to explore alternative sources of bis-sulfenate synthon [37].

## 2. Results and Discussion

**Figure 3:**
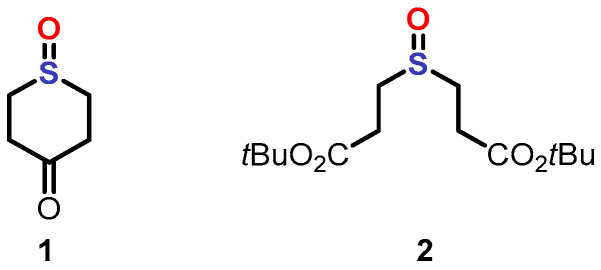
New sources of bis-sulfenate synthons.

Thanks to our team experience with the β-H elimination systems for generation of sulfenate anions, we have rapidly identified two candidates as new sources of bis-sulfenate synthon: the commercially available tetrahydro-4*H*-thiopyran-4-one 1-oxide **1** as a cyclic precursor and sulfinyl-di-*tert*-butylpropionate **2** as a linear precursor (Figure 3).

**Figure 4:**
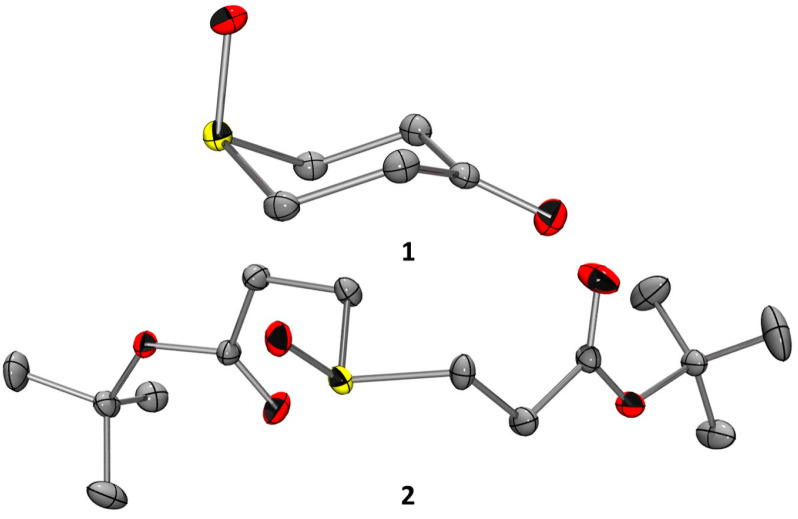
ORTEP view of X-ray structure of **1** and **2** at 50% probability; hydrogen atoms and solvent molecule have been omitted for clarity.

Tetrahydro-4*H*-thiopyran-4-one 1-oxide **1** and sulfinyl-di-*tert*-butylpropionate **2** appear as white crystalline solids that are stable for months when kept in dry conditions (Figure 4). Chemical **2** was synthesized by esterification reaction of inexpensive thiodipropionic acid with *tert*-butyl alcohol in acidic conditions, then oxidation. This two-step procedure is efficient and can be performed on a multigram scale (14.5 g). However, when exposed to moisture, tetrahydro-4*H*-thiopyran-4-one 1-oxide **1** degrades into the corresponding gem diol in the solid state and in solution. Importantly, the latter is unreactive in the catalytic system described herein.

We began our catalytic studies with **1** using 4-iodotoluene **3a** as the model substrate (Table 1). Pleasingly, upon using classic sulfenate cross-coupling conditions, bis(dibenzylideneacetone)palladium(0) as the palladium source, Xantphos as ligand and cesium carbonate as the base in toluene at 110 °C, we obtained the desired product **4a** with 48% yield (entry 1). Noteworthy, 10% of scrambling product (4-tolylsulfinyl-benzene) was obtained as well. This side product, arising from the aryl exchange on Xantphos palladium oxidative addition complexes, is well known and typically observed in sulfenate cross coupling [24]. Surprisingly, Pd_2_(dba)_3_, often used as a palladium(0) source in sulfenate cross-couplings, led to no conversion (entry 2). Different ligands such as SPhos, XPhos or DPEPhos (entries 3–5) or palladium catalysts such as CX21 [Allylchloro[1,3-bis(2,6-diisopropylphenyl)-imidazol-2-ylidene] palladium(II)] (entry 6) were thus investigated, not providing any product and evidencing that Xantphos is the ligand of choice for this cross-coupling reaction. Upon lowering the catalytic charge to 2 mol%, the reaction became sluggish (entries 7–8), convincing us to further optimize the reaction conditions with 5 mol%. The optimization of the base then proved critical in this reaction (entries 9–12). Indeed, when DBU was used, a drastic improvement of the yield to 61% was obtained along with only a very small amount of scrambling product (entry 11). Remarkably, lowering the temperature to 30 °C did not affect the reaction yield, providing the product with 62% yield (entry 12). Noteworthy, to the best of our knowledge, those reaction conditions are the smoothest and most selective scrambling-wise reported to date.

Nonetheless, despite allowing the cross-coupling in smooth conditions along with small amounts of scrambling product, the isolated yield remains quite low using **1** as a bis-sulfenate source, presumably because of its degradation in basic media happening too quickly for the catalytic system. We then naturally turned to the use of sulfinyl-di-*tert*-butylpropionate **2** as a more robust reactant under the reaction conditions.

We first used the reaction conditions previously developed for **1** using 4-iodotoluene **3a** [Pd(dba)_2_/Xantphos, DBU as base at 30 °C], which provided the desired biarylsulfoxide **4a** with 71% yield. However, the reaction needed 72 h for completion at this temperature. We then carried out the reaction at 80 °C and obtained the product **4a** with 83% yield in 24 h. Encouraged by these results, we decided to continue the optimization of the reaction conditions on the more challenging sterically hindered 1-iodonaphatalene **3t** (Table 2). Indeed, the desired product was not observed within the previously developed conditions, even upon increasing the reaction temperature to 80 °C (entry 1). We thus reasoned that the sulfinyl-di-*tert*-butylpropionate **2** was being degraded too quickly in the presence of DBU. Gratifyingly, changing the base to Cs_2_CO_3_ furnished the desired 1,1′-sulfinyldinaphthalene **4t** in 80% of isolated yield (entry 2). Finally, using the Buchwald precatalyst XantPhosPdG3, which is easy to handle and known to furnish highly active catalytic Pd(0) species under basic conditions, provided a major improvement of the reaction both in catalytic charge (2 mol%) and isolated yield (95%) (entry 3).

**Scheme 2:**
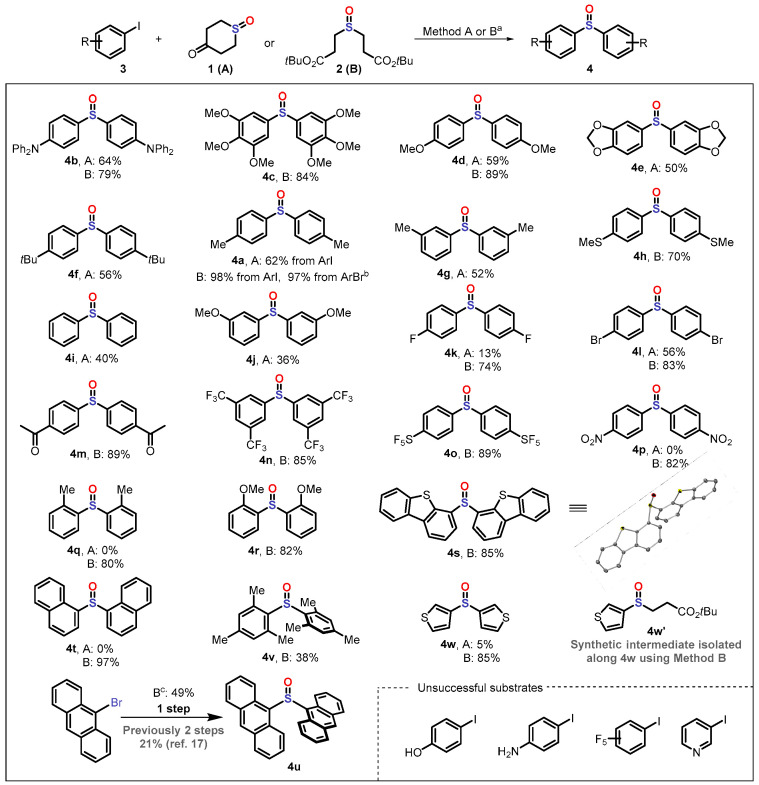
Reaction scope of the pallado-catalyzed cross coupling of sulfenates with aryl iodides. ^a^ Reaction method A: iodoarene (0.9 mmol, 3.0 equiv.), 1 (0.3 mmol, 1.0 equiv.), Pd(dba)_2_ (5 mol%), Xantphos (5 mol%), DBU (1.2 mmol, 4 equiv.), PhMe (3 mL), 30 °C, 6 h. Reaction method B: iodoarene (0.9 mmol, 3.0 equiv.), 2 (0.3 mmol, 1.0 equiv.), XantphosPdG3 (2 mol%), Cs_2_CO_3_ (1.2 mmol, 4 equiv.), PhMe (3 mL), 80 °C, 18 h. ^b^ THF was used instead of PhMe. ^c^ Reaction was carried out at 110 °C using 5 mol% of XantphosPdG3.

After optimizing the reaction conditions with both reactants **1** and **2**, we turned our attention towards exploring their reaction scope and limitations. A large span of electronic effects was thus explored (Scheme 2). Electron-rich substrates are well tolerated using both catalytic systems, with method B using the reactant **2** generally providing the biarylsulfoxides **4** with higher yields. Noteworthy, obtaining **4h** in 70% yield in one step is a striking example of the advantage of the present methodology, the synthesis of this compound by an oxidation approach being really complicated. The reaction method A using **1** showed a drastic loss of activity upon investigating electron-poor iodoarenes with no conversion observed for groups more electron-withdrawing than halogens. Method B, though, allowed the cross-coupling reaction furnishing the highly electron-poor products such as pentafluorosulfanyl **4o** or nitro **4p** derivatives in 89% and 82% yield, respectively. Finally, we turned our attention to the study of steric effects. Although method A proved completely inefficient in the case of ortho-substituted substrates, method B showed robustness providing the desired products in good to excellent yields. Moreover, the cross-coupling could be adapted to accommodate aryl bromides. For example, the sterically demanding 9-bromoanthracene could be engaged in the reaction, increasing both the temperature to 110 °C and the catalyst loading to 5 mol%, furnishing the corresponding biarylsulfoxide **4u** in 49% yield.

**Scheme 3:**
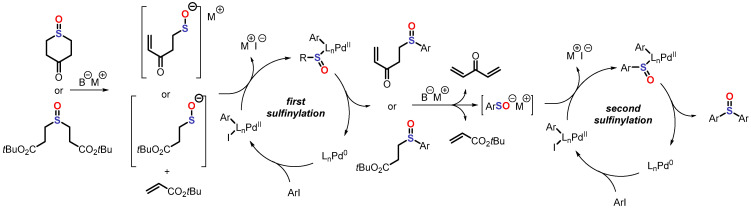
Proposed reaction mechanism for the double pallado-catalyzed cross-coupling of aryl iodides with the sulfenate anion.

Noteworthy, the only known synthesis of this compound required two steps and furnished the product with 21% overall yield [38]. In addition, this product **4u** is particularly interesting for the study of SO extrusion under irradiation [38,39]. Finally, 3-iodothiophene was engaged in the reaction conditions, once again highlighting the superiority of method B over A, providing the desired sulfoxide at 85% and 5% yield, respectively. Interestingly, the compound **4w**, synthesized in method B, was obtained as a 90/10 mixture with **4w′**, thereby confirming the anticipated stepwise cross-coupling mechanism (Scheme 3).

The results presented herein clearly show the superiority of method B using the reactant **2** over method A. The difference in reactivity presumably arises on one hand from the degradation rate of the bis-sulfenate in their respective reaction conditions. On the other hand, the temperature may have an impact on the cross-coupling kinetics itself. The best conditions thus lay in the best balance between the generation rate of the sulfenate and its consumption in the catalytic cross-coupling reaction.

**Figure 5:**
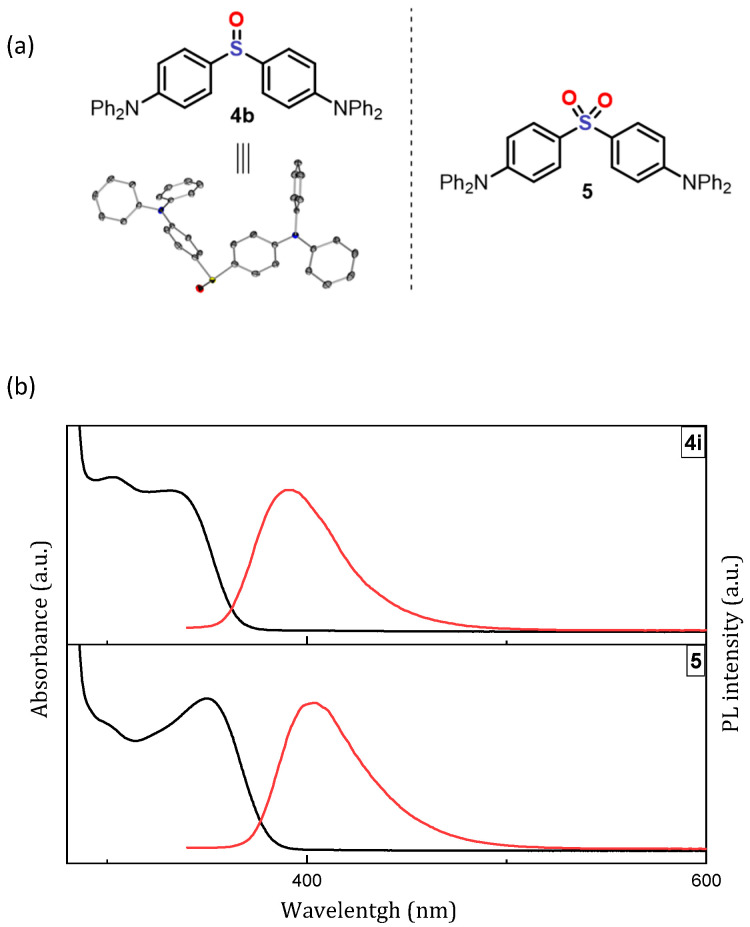
(**a**) Molecular structures of **4b** and **5** and ORTEP view of X-ray structure of **4b** at 50% probability. (**b**) Normalized emission and absorption spectra of **4b** and **5** in toluene at 298 K. black line: absorbance, red line: pl intensity.

Intrigued by the strong fluorescence observed upon handling **4b**, we decided to study its photophysical studies (Figure 5). Noteworthy, its parent sulfone **5** and some closely related derivatives have received much attention in the last decade because of their thermally activated delayed fluorescence (TADF), which makes them promising in a number of applications such as OLEDs [40,41,42]. In a toluene solution, **4b** presents a maximum of absorption at 333 nm and an emission band at 391 nm, while **5**′s maximum of absorption is at 352 nm and emits at 401 nm (Figure 5). Additionally, for both compounds, a strong solvatochromic fluorescence is observed, ranging from 372 nm to 461 nm in cyclohexane and methanol respectively for **4b**, and 383 nm to 462 nm for **5** (see Supplementary Materials). The wider range of **4b**’s solvatochromic emission (89 nm) compared to **5** (79 nm) probably arises from the higher polarizability of the sulfoxide moiety. Finally, we turned our attention towards the lifetime of the excited states (Table 3). We first focused on reproducing the studies on **5** and obtained comparable results with the literature, showing first a fast fluorescence decay component with a lifetime on 2.43 ns followed by a second, slower decay with a 111 μs lifetime, characteristic of the TADF phenomenon. Pleasingly, in a similar fashion, **4b** shown two fluorescence decay components with 0.93 ns and 94 μs of lifetime, thus showing that **4b** possess comparable TADF properties with its parent sulfone **5**. Finally, the luminescence quantum yields were measured, **5** displaying a high 69.1% whereas **4b** furnished a lower yield of 25.7%.

## 3. Materials and Methods

### 3.1. Reagents and Solvents

Unless otherwise noted, reagents were purchased from commercial suppliers and used directly without further purification. Unless indicated, technical grade solvents were purchased from commercial suppliers and used without further purification. Toluene was dried by passing over two columns of activated alumina, kept over activated 4A molecular sieves, and degassed by thorough argon sparging. All water was deionized before use. Unless stated, all reactions were carried out in conventional glassware. ‘Room temperature’ varied between 18 °C and 25 °C.

### 3.2. Analysis and Characterization

Analytical Thin Layer Chromatography (TLC) was performed on Merck aluminum-backed silica gel 60 F254 plates (Darmstadt, Germany). Developed TLC plates were visualized by ultraviolet (UV) irradiation (254 nm) or by staining with a solution of potassium permanganate. Column chromatography was carried using Merk silica gel 60 Å, 220–440 mesh. Fourier Transform Infrared Spectrometry (FTIR) was carried out using a Thermo Nicolet 6700 (Waltham, MA, USA) using an Attenuated Total Reflection (ATR) attachment and peaks are reported in terms of frequency of absorption (cm^−1^). High Resolution Mass Spectrometry (HRMS) data were acquired using a GCT Premier CAB109 TOF mass spectrometer (Milford, MA, USA) equipped with DCI-CH_4_ ionization. HRMS data were quoted to four decimal places (0.1 mDa). All NMR spectra were recorded on either a Bruker AV 300 or Bruker AV 500 (Billerica, MA, USA) and were internally referenced to residual solvent signals (residual CHCl_3_ was referenced at δ 7.26 and CDCl_3_ at δ 77.16 for ^1^H and ^13^C NMR, respectively, while residual CHDCl_2_ is referenced at δ 5.32 and CD_2_Cl_2_ at δ 53.84 for ^1^H and ^13^C NMR, respectively). All NMR chemical shifts (δ) were reported in parts per million (ppm) and coupling constants (*J*) are given in hertz (Hz). The ^1^H NMR spectra are reported as follows: δ (multiplicity, coupling constant *J*, number of protons), “app” stands for apparent.

The solution samples (0.1 mM) for the luminescence studies were prepared in an argon-filled glove-box using dry and degassed toluene. UV-VIS absorption spectra of the compounds in toluene were measured on an Agilent Cary 60 UV-Vis spectrophotometer in the range of 280–600 nm. The photoluminescence spectra were recorded on an Agilent Cary Eclipse spectrofluorometer (Santa Clara, CA, USA). The absolute PL quantum yield (PLQY) was obtained by using Quantaurus-QY Plus (HAMAMATSU, Shizuoka, Japan). The phosphorescence decay characteristics of the solution samples was recorded using a Horiba Fluorolog 3-2iHR 320 spectrofluorometer (Kyoto, Japan), equipped with a UV xenon flash lamp (FL-1035, 0.05–25 Hz flash rate, 3 µs pulse at FWHM). Excitation wavelengths were selected thanks to the double-monochromator of the spectrophotometer. The fast decay components were recorded in TCSPC mode thanks to a Horiba NanoLED module (Kyoto, Japan), piloting a pulsed diode NanoLED (371 nm). TADF measurements were recorded and analyzed using FluorEssence V3.8 from Horiba. Lifetime measurements were recorded with Data Station 2.7.0.4 and analyzed with DAS-6 6.8.0.10, from Horiba.

Single-crystal X-ray data were collected at low temperature (193(2)K) on a Bruker APEX II Quazar diffractometer (Billerica, MA, USA) equipped with a 30 W air-cooled microfocus source (**1**) or on a Bruker D8 VENTURE diffractometer equipped with a PHOTON III detector (**2**, **4b** and **4s**), using MoKα radiation (λ = 0.71037 Å). The structures were solved by intrinsic phasing method [43] and refined by full-matrix least- squares method on F2 [44]. All non-H atoms were refined with anisotropic displacement parameters and all the hydrogen atoms were refined isotropically at calculated positions using a riding model.

### 3.3. Synthesis of the Sulfenate Sources

Tetrahydro-4*H*-thiopyran-4-one 1-oxide 1:

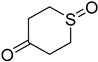



Prepared following a reported procedure [45]. Oxone^®^ (15.35 g, 50 mmol, 1 equiv.) was added to a stirred suspension of wet alumina (50 g, prepared from 50 g of neutral alumina mixed with 10 mL of water) in DCM (500 mL). Tetrahydro-4*H*-thiopyran-4-one (5.8 g, 50 mmol, 1 equiv.) was added in one portion, and the reaction mixture was stirred for 24 h at room temperature. The reaction mixture was filtered over a sintered funnel, rinsing with DCM (2×100 mL), before evaporating the solvent under reduced pressure. The crude mixture was then purified over silica gel chromatography (gradient from 30 to 100% EtOAc:Pentane) yielding the desired crystalline product (2.00 g, 15.2 mmol, 30%).

^1^H NMR (CDCl_3_, 300 MHz) δ_H_ 3.47–3.23 (m, 4H), 2.98–2.78 (m, 2H), 2.61–2.48 (m, 2H). ^13^C{^1^H} NMR (CDCl_3_, 75 MHz) δ_C_ 204.9, 47.5, 32.4.

Data are in agreement with the literature [46].

Di(*tert*-butyl)-3,3′-thiodipropionate:

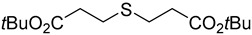



Prepared following a reported procedure [47]. Conc. H_2_SO_4_ (11 mL, 200 mmol, 2 equiv.) was added over a vigorously stirred suspension of anhydrous MgSO_4_ (96.2 g, 800 mmol, 8 eq.) in 500 mL of dichloromethane in a 1 L flask. After 15 min of stirring, thiodipropionic acid (17.8 g, 100 mmol, 1 equiv.) and *tert*-butanol (94 mL, 1.0 mol, 10 equiv.) were added before quickly sealing the flask with a new rubber septum and the reaction mixture was stirred for 65 h at room temperature. A volume of 300 mL of saturated aq. NaHCO_3_ was carefully added in portions before transferring the reaction mixture in a separatory funnel. The organic phase was separated and treated once with brine, then dried over Na_2_SO_4_. The volatiles were then removed in a rotavapor to obtain an oily residue and purified over silica gel column chromatography (gradient from 0 to 3% EtOAc:Pentane), yielding the desired product as a colorless oil (17.2 g, 59 mmol, 59% yield)

^1^H NMR (CDCl_3_, 300 MHz) δ_H_ 2.75 (t, *J* = 7.5 Hz, 4H), 2.51 (t, *J* = 7.4 Hz, 4H), 1.45 (s, 18H). ^13^C{^1^H} NMR (75 MHz, CDCl_3_) δ_c_ 171.3, 81.0, 36.2, 28.2, 27.3. HRMS (DCI-CH_4_) Calc’d for C_14_H_26_O_4_S [M]^+^ 290.1552, found 290.1561. FTIR (neat) ν_max_/cm^−1^ 2978, 1724, 1366, 1245, 1139, 1046, 844, 755.

Di(*tert*-butyl)-3,3′-thiodipropionate S-oxide 2:

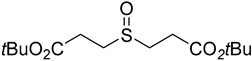



Prepared following a reported procedure [45]. Di(*tert*-butyl)-3,3′-thiodipropionate (17.2 g, 59 mmol, 1 equiv.) was added to a vigorously stirred suspension of wet alumina (60 g, prepared from 50 g of neutral alumina mixed with 10 mL of water) and Oxone^®^ (18.1 g, 59 mmol, 1 equiv.) in 300 mL of CH_2_Cl_2_. The mixture was left stirring for 18 h and filtrated over a sintered funnel. The solids were washed with EtOAc and the filtrate was evaporated under reduced pressure. The crude mixture was then purified over silica gel chromatography (gradient from 0 to 60% EtOAc;Pentane) yielding a pure colorless product, crystallizing upon standing (14.6 g, 47.6 mmol, 81%).

^1^H NMR (CDCl_3_, 300 MHz) δ_H_ 2.97 (ddd, *J* = 12.9, 8.2, 7.4 Hz, 2H), 2.83 (app dt, *J* = 12.9, 6.6 Hz, 2H), 2.73–2.66 (m, 4H), 1.41 (s, 18H). ^13^C{^1^H} NMR (75 MHz, CDCl_3_) δ_C_ 170.4, 81.7, 47.4, 28.2, 28.1. HRMS (DCI-CH_4_) Calc’d for C_14_H_26_O_5_S [M]^+^ 306.1501, found 315.1509. FTIR (neat) ν_max_/cm^−1^ 2980, 2932, 1702, 1364, 1226, 1154, 1032, 950, 844, 754.

### 3.4. General Procedures for the Pallado-Catalyzed Cross Coupling Reactions

Method A:

Dry and degassed toluene (3 mL) was added to a Schlenk tube containing Pd(dba)_2_ (8.6 mg, 0.015 mmol, 5 mol%) and Xantphos (8.7 mg, 0.015 mmol, 5 mol%) under inert atmosphere. The solution was stirred for 2 min, then degassed DBU (180 μL, 1.2 mmol, 4 equiv.) was added, inducing a color change of the solution from dark red to clear orange-yellow. A second Schlenk tube containing tetrahydro-4*H*-thiopyran-4-one 1-oxide (39.6 mg, 0.3 mmol, 1 equiv.) and solid aryl iodide (0.9 mmol, 3 equiv.) was evacuated and back-filled with argon three times. Liquid aryl iodides (0.9 mmol, 3 equiv.) were added subsequently to evacuation and back-filling. The palladium solution was then cannulated in the second Schlenk tube, and the resulting reaction mixture was stirred at 30 °C for 6 h. Then, the solution was evaporated to dryness and purified over silica gel column chromatography.

Method B:

Sulfinyl-di-*tert*-butylpropionate (91.9 mg, 0.3 mmol, 1 equiv.), Xantphos-Phos-Pd-G3 (5.7 mg, 0.006 mmol, 2 mol%), aryl iodide (0.9 mmol, 3 equiv.) and Cs_2_CO_3_ (391 mg, 1.2 mmol, 4 equiv.) were weighted in a Schlenk tube. The Schlenk tube was capped with a rubber septum before being evacuated and backfilled with argon three times. A volume of 3 mL of dry and degassed toluene was added and the reaction mixture was heated at 80 °C for 16 h. A volume of 20 mL of distilled water and 20 mL of dichloromethane were added, and the organic phase separated; the aqueous phase was subsequently extracted with dichloromethane twice. The combined organic phases were dried over Na_2_SO_4_ before removing the volatiles using a rotavapor. The crude product was then purified over silica gel column chromatography.

### 3.5. Biarylsulfoxide Characterization

4,4′-sulfinylbis(N,N-diphenylaniline) 4b:

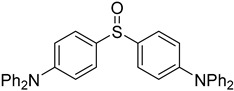



Synthesized according to the general procedure using 4-iodo-*N*,*N*-diphenylaniline (334 mg, 0.9 mmol). Purification by flash column chromatography (gradient from 0 to 25% EtOAc:Pentane) afforded the title compound as a white foam (Method A: 102.8 mg, 0.19 mmol, 64%: Method B: 127.3 mg, 0.24 mmol, 79%).

^1^H NMR (CDCl_3_, 300 MHz) δ_H_ 7.44 (d, *J* = 8.8 Hz, 4H), 7.29 (dd, *J* = 8.5, 6.8 Hz, 8H), 7.15–7.08 (m, 12H), 7.06 (d, *J* = 8.9 Hz, 4H). ^13^C{^1^H} NMR (CDCl_3_, 75 MHz) δ_C_ 150.6, 146.9, 136.8, 129.7, 126.7, 125.7, 124.4, 121.7. HRMS (DCI-CH_4_) Calc’d for C_36_H_29_N_2_OS [M + H]^+^ 537.2001, found 537.2009. FTIR (neat) ν_max_/cm^−1^ 3057, 2923, 1580, 1486, 1315, 1272, 1090, 1045, 754, 696.

5,5′-sulfinylbis(1,2,3-trimethoxybenzene) 4c:

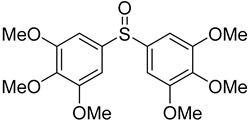



Synthesized according to the general procedure using 5-iodo-1,2,3-trimethoxybenzene (265 mg, 0.9 mmol). Purification by flash column chromatography (gradient from 0 to 80% EtOAc:Pentane) afforded the title compound as a pale yellow amorphous solid (Method B: 96.2 mg, 0.252 mmol, 84%).

^1^H NMR (CDCl_3_, 300 MHz) δ_H_ 6.87 (s, 4H), 3.88 (s, 12H), 3.86 (s, 6H). ^13^C{^1^H} NMR (CDCl_3_, 75 MHz) δ_C_ 154.1, 140.4, 140.2, 101.9, 61.1, 56.6. HRMS (DCI-CH_4_) Calc’d for C_18_H_23_O_7_S [M + H]^+^ 383.1165 found 383.1161. FTIR (neat) ν_max_/cm^−1^ 3084, 3053, 2939, 1590, 1496, 1462, 1409, 1307, 1235, 1127, 1102, 1059, 1004.

4,4′-sulfinylbis(methoxybenzene) 4d:

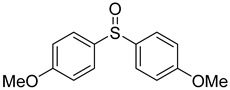



Synthesized according to the general procedure using 4-iodo-anisole (211 mg, 0.9 mmol). Purification by flash column chromatography (gradient from 0 to 25% EtOAc:Pentane) afforded the title compound as a beige amorphous solid (Method A: 46.3 mg, 0.18 mmol, 59% Method B: 69.8 mg, 0.27 mmol, 89%).

^1^H NMR (CDCl_3_, 300 MHz) δ_H_ 7.52 (d, *J* = 8.8 Hz, 4H), 6.94 (d, *J* = 8.8 Hz, 4H), 3.79 (s, 6H). ^13^C{^1^H} NMR (CDCl_3_, 75 MHz) δ_C_ 161.9, 137.1, 126.9, 114.8, 55.6.

Data are in agreement with the literature [48].

5,5′-sulfinylbis(benzo[d][1,3]dioxole) 4e:

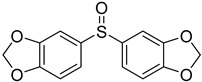



Synthesized according to the general procedure using 5-iodo-1,3-benzodioxole (114 μL, 0.9 mmol). Purification by flash column chromatography (gradient from 0 to 30% EtOAc:Pentane) afforded the title compound as a beige amorphous solid (Method A: 43.9 mg, 0.15 mmol, 50%).

^1^H NMR (CDCl_3,_ 300 MHz) δ_H_ 7.16 (dd, *J* = 8.0, 1.7 Hz, 2H), 7.00 (d, *J* = 1.7 Hz, 2H), 6.85 (d, *J* = 8.0 Hz, 2H), 5.99 (app q, *J* = 1.4 Hz, 4H). ^13^C{^1^H} NMR (CDCl_3_, 75 MHz) δ_C_ 150.4, 148.8, 139.1, 120.0, 108.7, 104.9, 102.0.

Data are in agreement with the literature [48].

4,4′-sulfinylbis(tert-butylbenzene) 4f:

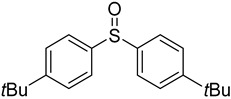



Synthesized according to the general procedure using 4-tert-butyliodobenzene (160 μL, 0.9 mmol). Purification by flash column chromatography (gradient from 0 to 15% EtOAc:Pentane) afforded the title compound as a beige amorphous solid (Method A: 52.5 mg, 0.17 mmol, 56%).

^1^H NMR (CDCl_3_, 300 MHz) δ_H_ 7.57 (d, *J* = 8.6 Hz, 4H), 7.47 (d, *J* = 8.6 Hz, 4H), 1.30 (s, 18H). ^13^C{^1^H} NMR (CDCl_3_, 75 MHz) δ_C_ 154.6, 142.5, 126.4, 125.0, 35.1, 31.3. HRMS (DCI-CH_4_) Calc’d for C_20_H_27_OS [M + H]^+^ 315.1783, found 315.1776. FTIR (neat) ν_max_/cm^−1^ 3058, 3023, 2961, 2929, 2868, 1650, 2592, 1397, 1267, 1083, 1042, 1009, 840, 829, 589, 564.

4,4′-sulfinylbis(methylbenzene) 4a:

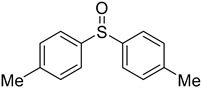



Synthesized according to the general procedure using 4-iodotoluene (196 mg, 0.9 mmol). Purification by flash column chromatography (gradient from 0 to 20% EtOAc:Pentane) afforded the title compound as a beige amorphous solid (Method A: 42.4 mg, 0.18 mmol, 55%; Method B: 67.9 mg, 0.295 mmol, 98%).

Synthesized according to the general procedure using 4-bromotoluene (154 mg, 0.9 mmol), using THF in place of PhMe (Method B: 67.0 mg, 0.29 mmol, 97%).

^1^H NMR (CDCl_3_, 300 MHz) δ_H_ 7.51 (d, *J* = 8.2 Hz, 4H), 7.25 (d, *J* = 8.1 Hz, 4H), 2.36 (s, 6H). ^13^C{^1^H} NMR (CDCl_3_, 75 MHz) δ_C_ 142.8, 141.5, 130.1, 125.0, 21.5.

Data are in agreement with the literature [24].

3,3′-sulfinylbis(methylbenzene) 4g:

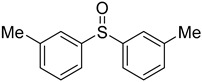



Synthesized according to the general procedure using 3-iodotoluene (115 μL, 0.9 mmol). Purification by flash column chromatography (gradient from 0 to 20% EtOAc:Pentane) afforded the title compound as a beige amorphous solid (Method A: 36.2 mg, 0.16 mmol, 52%).

^1^H NMR (CDCl_3_, 300 MHz) δ_H_ 7.47 (s, 2H), 7.41 (d, *J* = 7.7 Hz, 2H), 7.32 (t, *J* = 7.6 Hz, 2H), 7.22 (d, *J* = 7.4 Hz, 2H), 2.37 (s, 6H). ^13^C{^1^H} NMR (CDCl_3_, 75 MHz) δ_C_ 145.5, 139.6, 131.9, 129.2, 125.1, 122.1, 21.5.

Data are in agreement with the literature [49].

(sulfinylbis(4,1-phenylene))bis(methylsulfane) 4h:

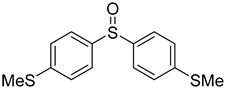



Synthesized according to the general procedure using (4-iodophenyl)(methyl)sulfane (225 mg, 0.9 mmol). Purification by flash column chromatography (gradient from 0 to 30% EtOAc:Pentane) afforded the title compound as beige amorphous solid (Method B: 62.2 mg, 0.21 mmol, 70%).

^1^H NMR (CDCl_3_, 300 MHz) δ_H_ 7.51 (d, *J* = 8.6 Hz, 4H), 7.27 (d, *J* = 8.6 Hz, 4H), 2.47 (s, 6H). ^13^C{^1^H} NMR (CDCl_3_, 75 MHz) δ_C_ 143.4, 141.7, 126.3, 125.4, 15.2. HRMS (DCI-CH_4_) Calc’d for C_14_H_15_OS [M+H]^+^ 295.0285, found 295.0279. FTIR (neat) νmax/cm^−1^ 3051, 2985, 2920, 1575, 1476, 1435, 1391, 1095, 1071, 1046, 811, 744, 554.

Sulfinyldibenzene 4i:

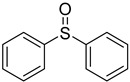



Synthesized according to the general procedure using iodobenzene (100 μL, 0.9 mmol). Purification by flash column chromatography (gradient from 0 to 20% EtOAc:Pentane) afforded the title compound as a beige amorphous solid (Method A: 27.4 mg, 0.12 mmol, 40%).

^1^H NMR (CDCl_3_, 300 MHz) δ_H_ 7.71–7.59 (m, 4H), 7.54–7.34 (m, 6H). ^13^C{^1^H} NMR (CDCl_3_, 75 MHz) δ_C_ 145.7, 131.2, 129.5, 124.9.

Data are in agreement with the literature [48].

3,3′-sulfinylbis(methoxybenzene) 4j:

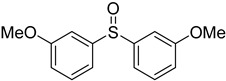



Synthesized according to the general procedure using 3-iodoanisole (107 μL, 0.9 mmol). Purification by flash column chromatography (gradient from 0 to 30% EtOAc:Pentane) afforded the title compound as a beige amorphous solid (Method A: 28.2 mg, 0.11 mmol, 36%).

^1^H NMR (CDCl_3_, 300 MHz) δ_H_ 7.34 (t, *J* = 7.9 Hz, 2H), 7.23 (dd, *J* = 2.4, 1.6 Hz, 2H), 7.22–7.12 (m, 2H), 3.81 (s, 6H). ^13^C{^1^H} NMR (CDCl_3_, 75 MHz) δ_C_ 160.5, 147.0, 130.4, 117.5, 117.1, 109.2, 55.7. HRMS (DCI-CH_4_) Calc’d for C_14_H_15_O_3_S [M+H]^+^ 263.0742, found 263.0733. FTIR (neat) νmax/cm^−1^ 3062, 2957, 2924, 2854, 1725, 1593, 1578, 1477, 1247, 1037, 781, 689.

4,4′-sulfinylbis(fluorobenzene) 4k:

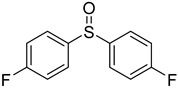



Synthesized according to the general procedure using 4-fluoroiodobenzene (104 μL, 0.9 mmol). Purification by flash column chromatography (gradient from 0 to 20% EtOAc:Pentane) afforded the title compound as a beige amorphous solid (Method A: 12.9 mg, 0.054 mmol, 13%, Method B: 53.2 mg, 0.22 mmol, 74%).

^1^H NMR (CDCl_3_, 300 MHz) δ_H_ 7.61 (dd, *J* = 8.9, 5.1 Hz, 4H), 7.15 (t, *J* = 8.6 Hz, 4H). ^13^C{^1^H} NMR (CDCl_3_, 75 MHz) δ_C_ δ 164.5 (d, *J_C-F_* = 252.2 Hz), 141.1 (d, *J_C-F_* = 2.3 Hz), 127.2 (d, *J_C-F_* = 9.0 Hz), 116.9 (d, *J_C-F_* = 22.6 Hz).

Data are in agreement with the literature [48].

4,4′-sulfinylbis(bromobenzene) 4l:

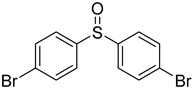



Synthesized according to the general procedure using 1-bromo-4-iodobenzene (255 mg, 0.9 mmol). Purification by flash column chromatography (gradient from 0 to 20% EtOAc:Pentane) afforded the title compound as a beige amorphous solid (Method A: 60.4 mg, 0.17 mmol, 56%, Method B: 89.8 mg, 0.25 mmol, 83%).

^1^H NMR (CDCl_3_, 300 MHz) δ_H_ 7.66–7.55 (m, 4H), 7.55–7.44 (m, 4H). ^13^C{^1^H} NMR (CDCl_3_, 75 MHz) δ_C_ 144.5, 132.9, 126.3, 126.1.

Data are in agreement with the literature [50].

1,1′-(sulfinylbis(4,1-phenylene))bis(ethan-1-one) 4m:

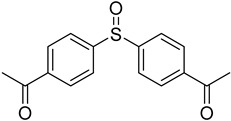



Synthesized according to the general procedure using 1-(4-iodophenyl)ethan-1-one (221.4 mg, 0.9 mmol). Purification by flash column chromatography (gradient from 0 to 70% EtOAc:Pentane) afforded the title compound as a pale yellow amorphous solid (Method B: 76.8 mg, 0.27 mmol, 89%).

^1^H NMR (CDCl_3_, 300 MHz) δ_H_ 8.04 (d, *J* = 8.6 Hz, 4H), 7.77 (d, *J* = 8.6 Hz, 4H), 2.60 (s, 6H). ^13^C{^1^H} NMR (CDCl_3_, 75 MHz) δ_C_ 197.0, 150.1, 139.4, 129.5, 124.8, 26.9.

Data are in agreement with the literature [51].

5,5′-sulfinylbis(1,3-bis(trifluoromethyl)benzene) 4n:

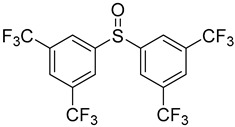



Synthesized according to the general procedure using 1-iodo-3,5-bis(trifluoromethyl)benzene (159 μL, 0.9 mmol). Purification by flash column chromatography (gradient from 0 to 10% EtOAc:Pentane) afforded the title compound as a pale yellow amorphous solid (Method B: 120.9 mg, 0.26 mmol, 85%).

^1^H NMR (CDCl_3_, 600 MHz) δ_H_ 8.16 (s, 4H), 8.02 (s, 2H). ^13^C{^1^H} NMR (CDCl_3_, 151 MHz) δ_C_ 147.8, 133.8 (q, *J* = 34.6 Hz), 126.0 (p, *J* = 3.6 Hz), 124.7 (d, *J* = 3.7 Hz), 122.5 (q, *J* = 273.7 Hz).

Data are in agreement with the literature [52].

(sulfinylbis(4,1-phenylene))bis(pentafluoro-λ^6^-sulfane) 4o:

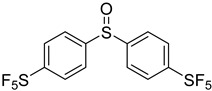



Synthesized according to the general procedure using pentafluoro(4-iodophenyl)-λ^6^-sulfane (297 mg, 0.9 mmol). Purification by flash column chromatography (gradient from 0 to 20% EtOAc:Pentane) afforded the title compound as a pale yellow amorphous solid (Method B: 121.1 mg, 0.27 mmol, 89%).

^1^H NMR (CDCl_3_, 300 MHz) δ_H_ 7.90 (d, *J* = 8.9 Hz, 4H), 7.79 (d, *J* = 9.1 Hz, 4H). ^13^C{^1^H} NMR (CDCl_3_, 151 MHz) δ_C_ 156.1 (p, *J* = 18.4 Hz), 148.7, 127.6 (p, *J* = 4.8 Hz), 125.0. ^19^F{^1^H} NMR (CDCl_3_, 282 MHz) δ_F_ −62.97, -102.95–-103.26 (m). HRMS (DCI-CH_4_) Calc’d for C_12_H_8_OF_10_S_3_ [M]^+^ 453.9578, found 453.9588. FTIR (neat) νmax/cm^−1^ 3103, 3068, 2928, 1605, 1480, 1397, 1103, 1057, 840, 724, 666, 601.

4,4′-sulfinylbis(nitrobenzene) 4p:

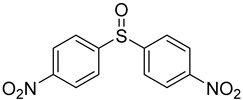



Synthesized according to the general procedure using 1-iodo-4-nitrobenzene (224.1 mg, 0.9 mmol). Purification by flash column chromatography (gradient from 0 to 10% EtOAc:Pentane) afforded the title compound as a pale yellow amorphous solid (Method B: 71.7 mg, 0.25 mmol, 82%).

^1^H NMR (CDCl_3,_ 300 MHz) δ_H_ 8.36 (d, *J* = 8.8 Hz, 4H), 7.90 (d, *J* = 8.8 Hz, 4H). ^13^C{^1^H} NMR (CDCl_3_, 75 MHz) δ_C_ 151.6, 149.9, 125.5, 125.1.

Data are in agreement with the literature [50].

2,2′-sulfinylbis(methylbenzene) 4q:

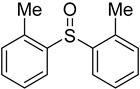



Synthesized according to the general procedure using 2-iodotoluene (114 μL, 0.9 mmol). Purification by flash column chromatography (gradient from 0 to 20% EtOAc:Pentane) afforded the title compound as a beige amorphous solid (Method B: 55.6 mg, 0.24 mmol, 80%).

^1^H NMR (CDCl_3,_ 300 MHz) δ_H_ 7.75–7.64 (m, 2H), 7.42–7.30 (m, 4H), 7.24–7.15 (m, 2H), 2.42 (s, 6H). ^13^C{^1^H} NMR (CDCl_3_, 75 MHz) δ_C_ 141.9, 136.7, 131.2, 131.0, 127.2, 126.1, 18.7.

Data are in agreement with the literature [49].

2,2′-sulfinylbis(methoxybenzene) 4r:

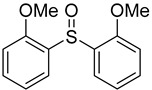



Synthesized according to the general procedure using 1-iodo-2-methoxybenzene (117 μL, 0.9 mmol). Purification by flash column chromatography (gradient from 0 to 60% EtOAc:Pentane) afforded the title compound as a beige amorphous solid (Method B: 64.9 mg, 0.25 mmol, 82%).

^1^H NMR (CDCl_3,_ 300 MHz) δ_H_ 7.65 (dd, *J* = 7.7, 1.7 Hz, 2H), 7.41 (ddd, *J* = 8.3, 7.4, 1.7 Hz, 2H), 7.07 (app. td, *J* = 7.5, 1.0 Hz, 2H), 6.89 (dd, *J* = 8.3, 1.0 Hz, 2H), 3.80 (s, 6H). ^13^C{^1^H} NMR (CDCl_3_, 75 MHz) δ_C_ 156.8, 132.5, 132.4, 127.0, 121.4, 111.3, 56.0.

Data are in agreement with the literature [49].

4,4′-sulfinyldidibenzo[*b*,*d*]thiophene 4s:

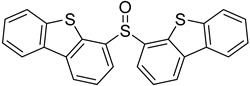



Synthesized according to the general procedure using 4-iododibenzo[*b*,*d*]thiophene (465 mg, 1.5 mmol, 3 equiv. Purification by flash column chromatography (gradient from 0 to 20% EtOAc:Pentane) afforded the title compound as a beige amorphous solid (Method B: 181.8 mg, 0.438 mmol, 85%).

^1^H NMR (CDCl_3,_ 300 MHz) δ_H_ 8.22 (dd, *J* = 7.9, 1.1 Hz, 2H), 8.18 (dd, *J* = 7.6, 1.1 Hz, 2H), 8.14–8.10 (m, 2H), 7.87–7.83 (m, 2H), 7.61 (app t, *J* = 7.7 Hz, 2H), 7.52–7.41 (m, 4H). ^13^C{^1^H} NMR (CDCl_3_, 75 MHz) δ_C_ 140.1, 137.9, 136.4, 136.2, 134.1, 127.7, 125.1, 124.9, 124.9, 124.6, 122.9, 121.9. HRMS (DCI-CH_4_) Calc’d for C_24_H_15_OS_3_ [M+H]^+^ 415.0285 found 415.0268. FTIR (neat) νmax/cm^−1^ 3053, 2922, 1434, 1398, 1389, 1056, 1028, 749, 703.

1,1′-sulfinyldinaphthalene 4t:

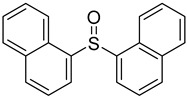



Synthesized according to the general procedure using 1-iodonaphthalene (131 μL, 0.9 mmol). Purification by flash column chromatography (gradient from 0 to 25% EtOAc:Pentane) afforded the title compound as a pale yellow amorphous solid (Method B: 87.7 mg, 0.29 mmol, 97%).

^1^H NMR (CDCl_3,_ 300 MHz) δ_H_ 8.42–8.30 (m, 2H), 8.06 (dd, *J* = 7.3, 1.2 Hz, 2H), 7.97 (app dt, *J* = 8.2, 1.1 Hz, 2H), 7.96–7.85 (m, 2H), 7.61–7.51 (m, 6H). ^13^C{^1^H} NMR (CDCl_3_, 75 MHz) δ_C_ 139.9, 133.8, 132.1, 130.1, 129.1, 127.8, 126.9, 125.8, 125.8, 122.7.

Data are in agreement with the literature [53].

9,9′-sulfinyldianthracene 4u:

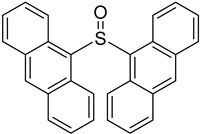



Synthesized according to the general procedure using 9-bromoanthracene (386 mg, 1.5 mmol, 3 equiv.), and carrying the reaction at 110 °C. Purification by flash column chromatography (gradient from 0 to 20% EtOAc:Pentane) afforded the title compound as a bright yellow solid (Method B: 98 mg, 0.24 mmol, 49%).

^1^H NMR (CD_2_Cl_2,_ 300 MHz) δ_H_ 9.32–9.13 (m, 4H), 8.51 (s, 2H), 8.05–7.90 (m, 4H), 7.52–7.32 (m, 8H). ^13^C{^1^H} NMR (CD_2_Cl_2_, 75 MHz) δ_C_ 134.5, 132.8, 131.6, 131.0, 129.9, 128.0, 125.8, 123.5.

Data are in agreement with the literature [38].

2,2′-sulfinylbis(1,3,5-trimethylbenzene) 4v:

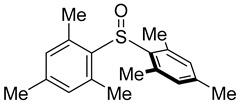



Synthesized according to the general procedure using 2-iodo-1,3,5-trimethylbenzene (221.5 mg, 0.9 mmol). Purification by flash column chromatography (gradient from 0 to 20% EtOAc:Pentane) afforded the title compound as a beige amorphous solid (Method B: 32.8 mg, 0.12 mmol, 38%).

^1^H NMR (CDCl_3,_ 300 MHz) δ_H_ 6.86–6.77 (m, 4H), 2.42 (s, 12H), 2.27 (s, 6H). ^13^C{^1^H} NMR (CDCl_3_, 75 MHz) δ_C_ 140.6, 138.6, 136.6, 131.3, 21.0, 19.6.

Data are in agreement with the literature [54].

3,3′-sulfinyldithiophene 4w:

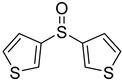



Synthesized according to the general procedure using 3-iodothiophene (91 μL, 0.9 mmol). Purification by flash column chromatography (gradient from 0 to 30% EtOAc:Pentane) afforded the title compound as a yellow oily residue (Method A: 3.4 mg, 0.02 mmol, 5%; Method B: 54.8 mg consisting of a 90/10 mixture of the titled compound and tert-butyl 3-(thiophen-3-ylsulfinyl)propanoate, 0.26 mmol, 85%).

^1^H NMR (CDCl_3_, 300 MHz) δ_H_ 7.80 (dd, *J* = 3.0, 1.3 Hz, 2H), 7.43 (dd, *J* = 5.1, 3.0 Hz, 2H), 7.11 (dd, *J* = 5.2, 1.3 Hz, 2H). ^13^C{^1^H} NMR (CDCl_3_, 75 MHz) δ_C_ 144.3, 128.5, 127.0, 124.2. HRMS (DCI-CH_4_) Calc’d for C_8_H_7_OS_3_ [M+H]^+^ 214.9659 found 215.9660. FTIR (neat) νmax/cm^−1^ 3090, 3066, 1398, 1204, 1096, 1072, 1040, 791, 633.

tert-butyl 3-(thiophen-3-ylsulfinyl)propanoate 4w′:

The titled compound was isolated as a co-eluting byproduct upon isolation of 3,3′-sulfinyldithiophene using method B (10%).

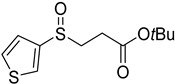



^1^H NMR (CDCl_3_, 300 MHz) δ_H_ 7.76 (dd, *J* = 3.0, 1.3 Hz, 1H), 7.51 (dd, *J* = 5.1, 3.0 Hz, 1H), 7.23 (dd, *J* = 5.1, 1.3 Hz, 1H), 3.20 (ddd, *J* = 13.4, 8.4, 6.9 Hz, 1H), 3.06 (ddd, *J* = 13.4, 8.2, 6.0 Hz, 1H), 2.75 (ddd, *J* = 17.1, 8.2, 6.8 Hz, 1H), 2.50 (ddd, *J* = 17.2, 8.4, 6.0 Hz, 1H), 1.43 (s, 9H).

### 3.6. Photophysical Properties of 4,4′-Sulfinylbis(N,N-Diphenylaniline) 4b and 4,4′-Sulfonylbis(N,N-Diphenylaniline) 5

Solutions of various concentrations of the desired compound were prepared in dry and degassed PhMe in an argon filled glove-box. The solution was transferred in a 3 mL quartz cuvette before sealing it tightly with a PTFE cap. The cuvette was removed from the glove-box and rapidly studied.

### 3.7. X-ray Data

CCDC-2364220 (**1**), CCDC-2364221 (**2**), CCDC-2364222 (**4b**) and CCDC-2364223 (**4s**) contain the supplementary crystallographic data for this paper (Sections S1, S2 and S3). These data can be obtained free of charge from The Cambridge Crystallographic Data Centre via www.ccdc.cam.ac.uk/structures (accessed on 10 October 2024).

## 4. Conclusions

In summary, we designed two new reactants, smoothly furnishing the bis-sulfenate anion synthon in basic conditions. These reactants were then applied in the development of a pallado-catalyzed methodology for the synthesis of symmetrical biarylsulfoxides tolerating a large array of electronic properties and bulkiness. Finally, the photophysical properties of the biarylsulfoxide **4b** have been explored, demonstrating an unreported TADF phenomenon on sulfoxide-containing scaffolds.

## Figures and Tables

**Table 1:** Optimization of the reaction conditions ^a^.

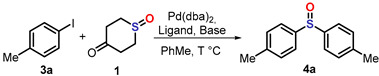			
Entry	Ligand	Base	Yield (%) ^b^
1	Xantphos (12%)	Cs_2_CO_3_	48 (10) ^c^
2	Xantphos (12%)	Cs_2_CO_3_	n.r. ^d^
3	SPhos (10%)	Cs_2_CO_3_	n.r. ^c^
4	XPhos (10%)	Cs_2_CO_3_	n.r. ^c^
5	DPEPhos (5%)	Cs_2_CO_3_	n.r. ^c^
6	CX21 (10%) ^e^	Cs_2_CO_3_	n.r.
7	Xantphos (5%)	Cs_2_CO_3_	33 (7)
8	Xantphos (2%) ^f^	Cs_2_CO_3_	16 (3)
9	Xantphos (5%)	K_3_PO_4_	12 (4)
10	Xantphos (5%)	DIPEA	n.r.
11	Xantphos (5%)	DBU	61 (2)
12	Xantphos (5%)	DBU	62 (1) ^g^

^a^ **3a** (0.3 mmol, 3.0 equiv.), **1** (0.1 mmol, 1.0 equiv.), Pd(dba)_2_ (5 mol%), base (0.4 mmol, 4 equiv.), PhMe (1 mL), 6 h, 110 °C. ^b^ isolated yield, yield of scrambling product within brackets, n.r. stands for no reaction. ^c^ 10 mol% of Pd(dba)_2_ used. ^d^ Pd_2_(dba)_3_ (5 mol%) used. ^e^ CX21 stands for Allylchloro[1,3-bis(2,6-diisopropylphenyl)-imidazol-2-ylidene]palladium(II). ^f^ Pd(dba)_2_ (2 mol%). ^g^ Temperature = 30 °C.

**Table 2:** Optimization of the reaction conditions ^a^.

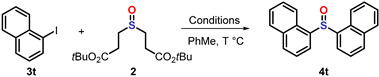		
Entry	Conditions	Yield (%) ^b^
1	Pd(dba)_2_ (5%), Xantphos (5%), DBU, 80 °C	n.r.
2	Pd(dba)_2_ (5%), Xantphos (5%), Cs_2_CO_3_, 80 °C	80 (6)
3	XantphosPdG3 (2%), Cs_2_CO_3_, 80 °C	95 (3)

^a^ **3t** (0.3 mmol, 3.0 equiv.), **2** (0.1 mmol, 1.0 equiv.), base (0.4 mmol, 4 equiv.), PhMe (1 mL), 18 h. ^b^ isolated yield, yield of scrambling product within brackets, n.r. stands for no reaction.

**Table 3:** Photophysical properties for compounds **4b** and **5** in toluene at 298 K.

Compounds	λ_max_ (nm)	*Φ* (%)	τ (ns/μs)
**4b**	391	25.7	0.93/94
**5**	401	69.1	2.43/111

## Data Availability

Data are available on request from the authors.

## Supplementary Materials

The following supporting information can be downloaded at: https://www.mdpi.com/article/10.3390/molecules29204809/s1, S1: Photophysical studies data. S2: NMR Spectra. S3: X-ray diffraction studies.
