# Supplementary material for: New Sulfenate Sources for Double Pallado-Catalyzed Cross-Coupling Reaction: Application in Symmetrical Biarylsulfoxide Synthesis, and Evidence of TADF Properties
Source: Molecules. 2024 Oct 11;29(20):4809. doi: 10.3390/molecules29204809 (PMC11509984; doi:10.3390/molecules29204809)
Supplement: Supplementary file 1 [file molecules-29-04809-s001.zip › molecules-3229392-supplementary.pdf]

Supplementary Materials for

New sulfenate sources for double pallado-catalyzed cross-coupling reaction: Application in Symmetrical Biarylsulfoxides synthesis, and evidence of TADF properties.

Valentin Magné,[1] Iulia Cretoiu,[1] Sonia Mallet-Ladeira,[2] Eddy Maerten,[1] David Madec[*1]

[1] Laboratoire Hétérochimie Fondamentale et Appliquée (UMR 5069), Université de Toulouse, CNRS, 118 Route de Narbonne, F-31062 Toulouse Cedex 09, France.

[2] Institut de Chimie de Toulouse (UAR 2599), 118 Route de Narbonne, 31062 Toulouse Cedex 09, France.

Correspondence: david.madec@univ-tlse3.fr

Table of content

S1 Photophysical studies of 4,4'-sulfinylbis(N,N-diphenylaniline) **4b** and 4,4'-sulfonylbis(N,N-diphenylaniline) **5**: 3

S2 NMR spectra: 6

S3 XRay diffraction data : 34

S1 Photophysical studies of 4,4'-sulfinylbis(N,N-diphenylaniline) **4b** and 4,4'-sulfonylbis(N,N-diphenylaniline) **5**:

Solutions of various concentrations of the desired compound were prepared in dry and degassed PhMe in an argon filled glove-box. The solution was transferred in a 3 mL quartz cuvette before sealing it tightly with a PTFE cap. The cuvette was removed from the glove-box and rapidly studied.

<u>Summary table:</u>

	Emission in PhMe (1.10^{-4}M)		
	λ_{max} (nm)	ϕ	τ (ns/µs)
**4b**	391	0.26	0.93/94
**5**	401	0.69	2.43/111

<u>Data obtained as following:</u>

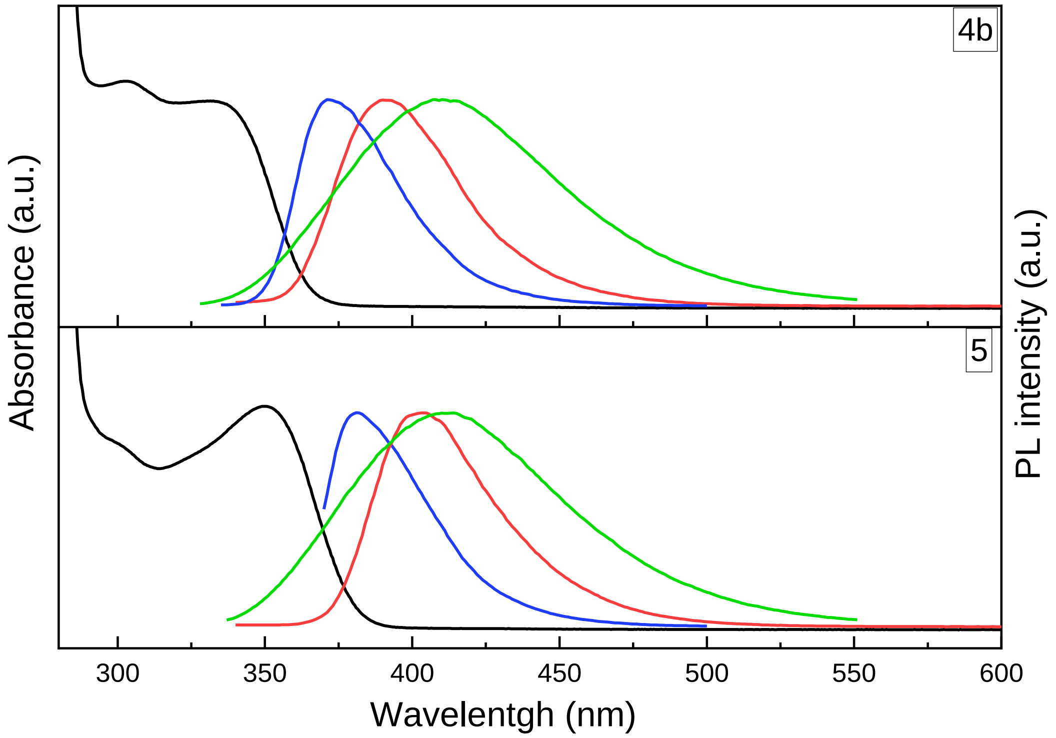

Black: Absorbance spectra, Blue: normalized emission spectra in cyclohexane, red: normalized emission spectra in toluene, green: normalized emission spectra in methanol.

4b Fluorescence decay (τ_1)

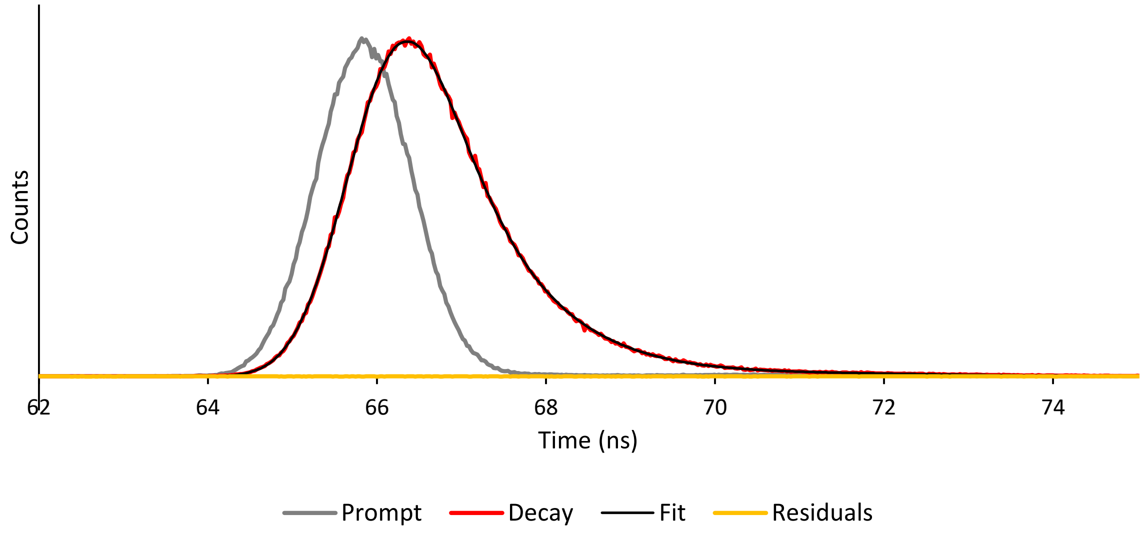

4b TADF decay (τ_2)

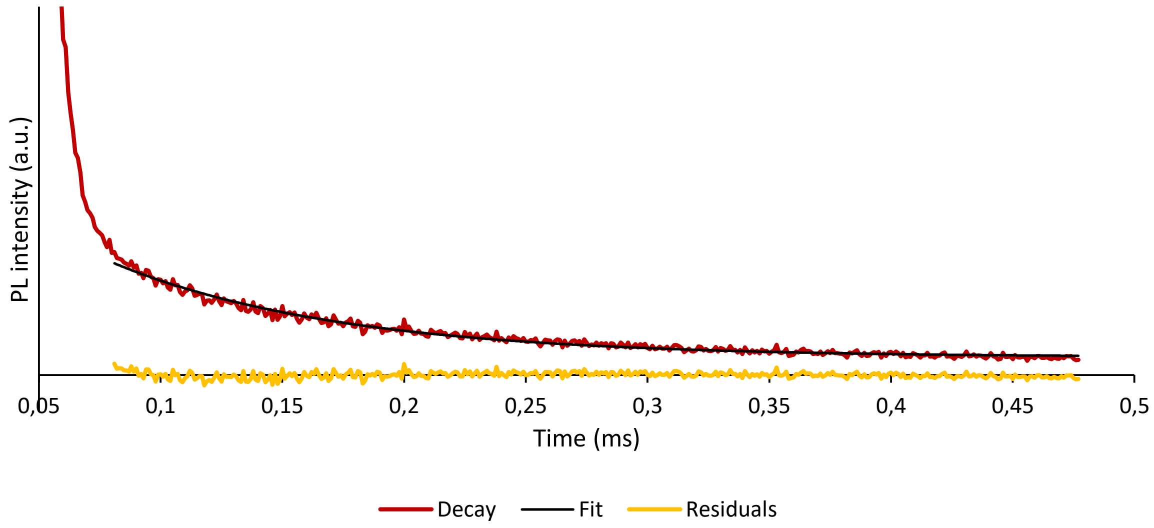

5 Fluorescence decay (τ_1)

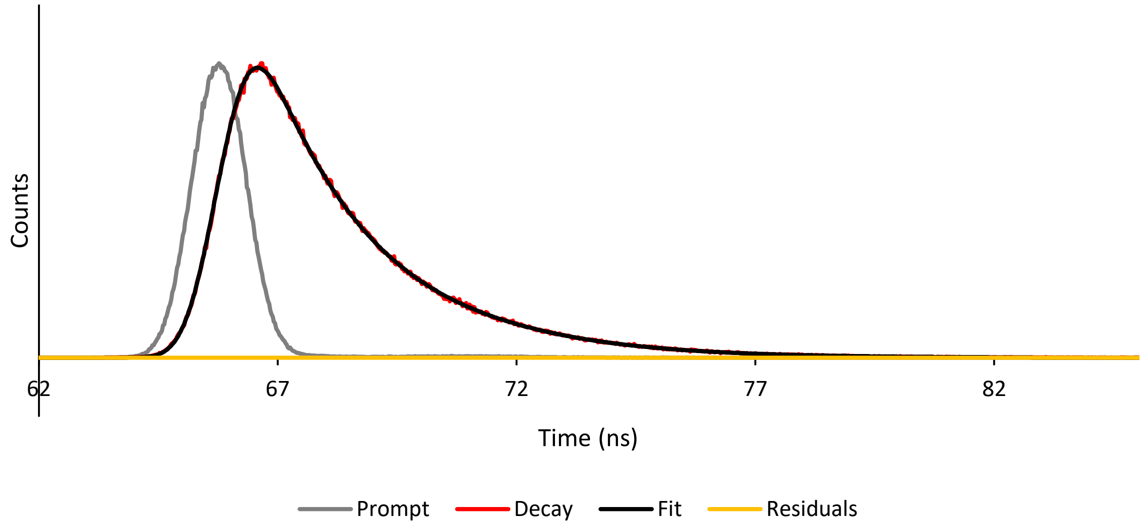

5 TADF decay (τ_2)

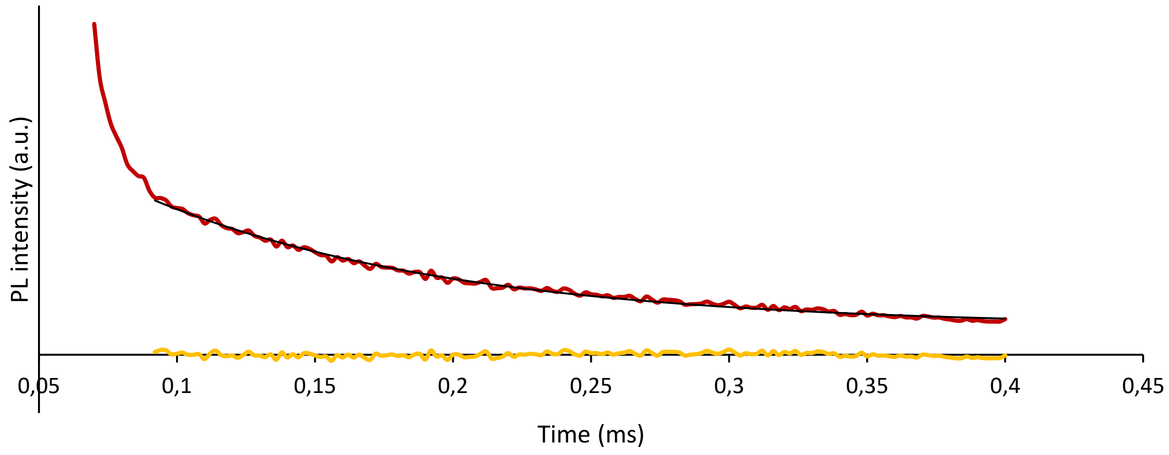

S2 NMR spectra:

Tetrahydro-4H-thiopyran-4-one 1-oxide 1:

^{1}H NMR ($CDCl_3$, 300 MHz)

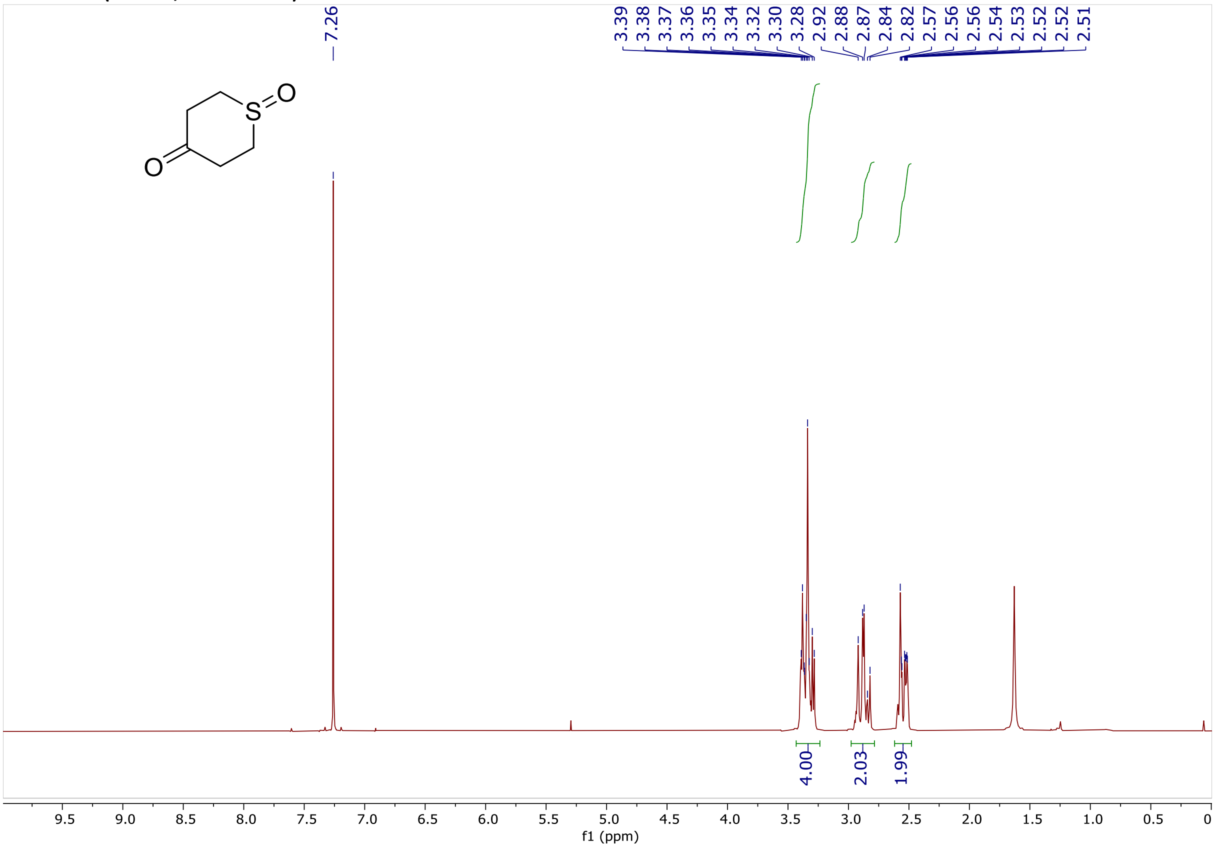

^{13}C{^{1}H} NMR ($CDCl_3$, 75 MHz)

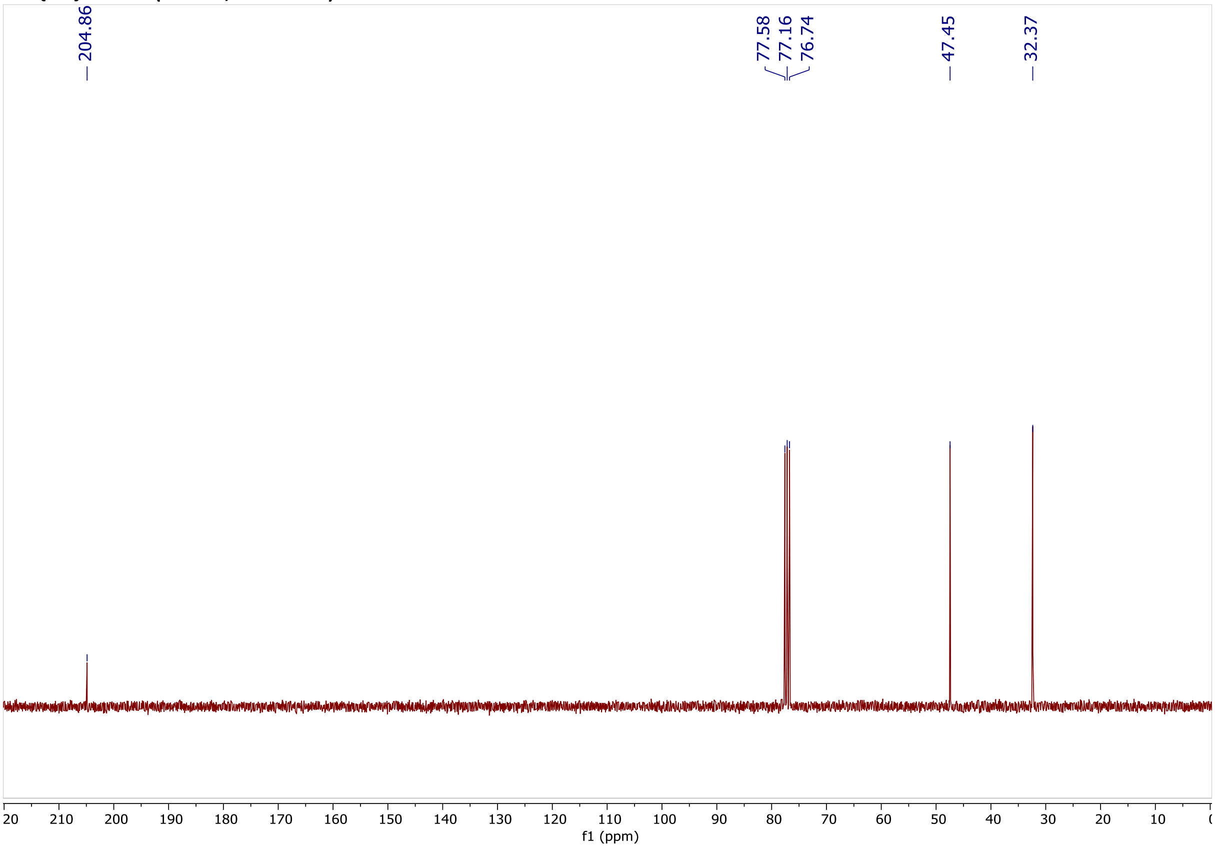

Di(tert-butyl)-3,3′-thiodipropionate:

^{1}H NMR ($CDCl_3$, 300 MHz)

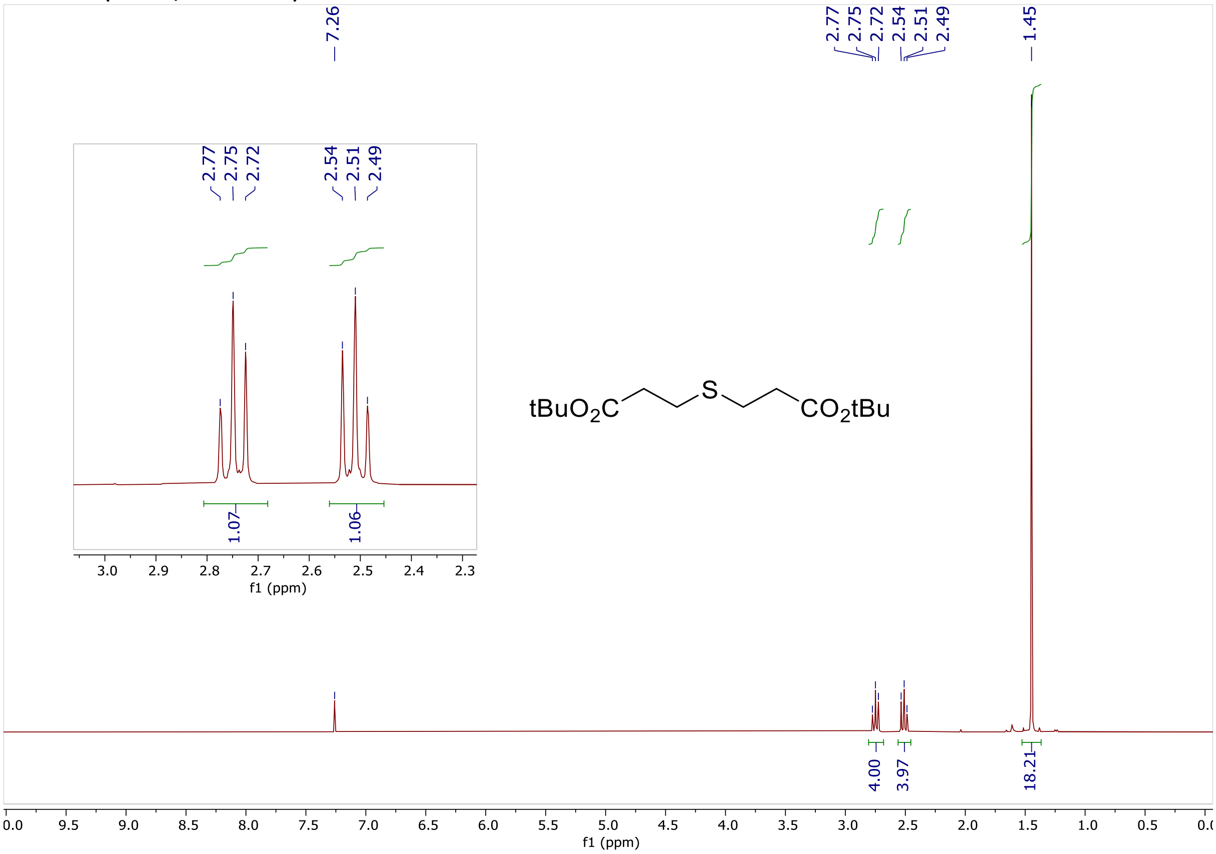

$^{13}C\{^{1}H\}$ NMR ($CDCl_3$, 75 MHz)

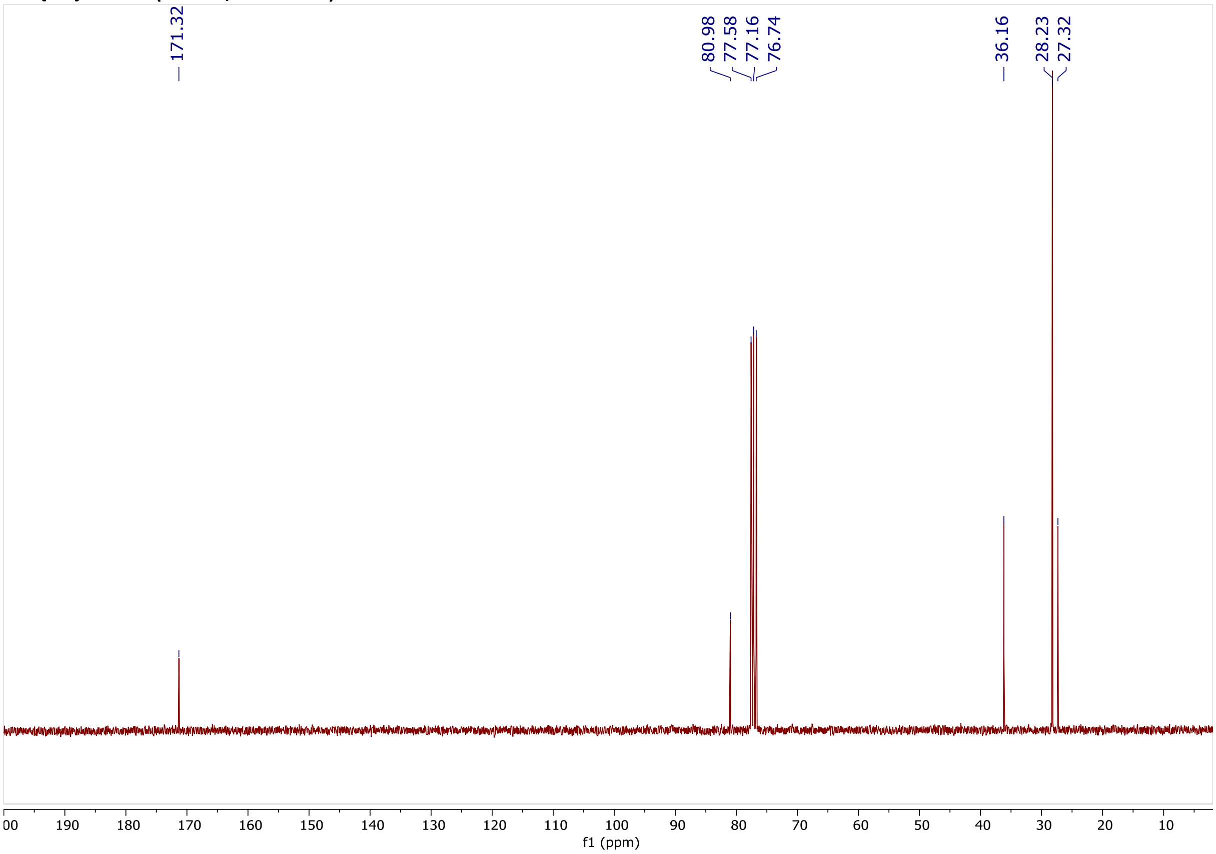

Di(tert-butyl)-3,3′-thiodipropionate S-oxide 2:

^{1}H NMR ($CDCl_3$, 300 MHz)

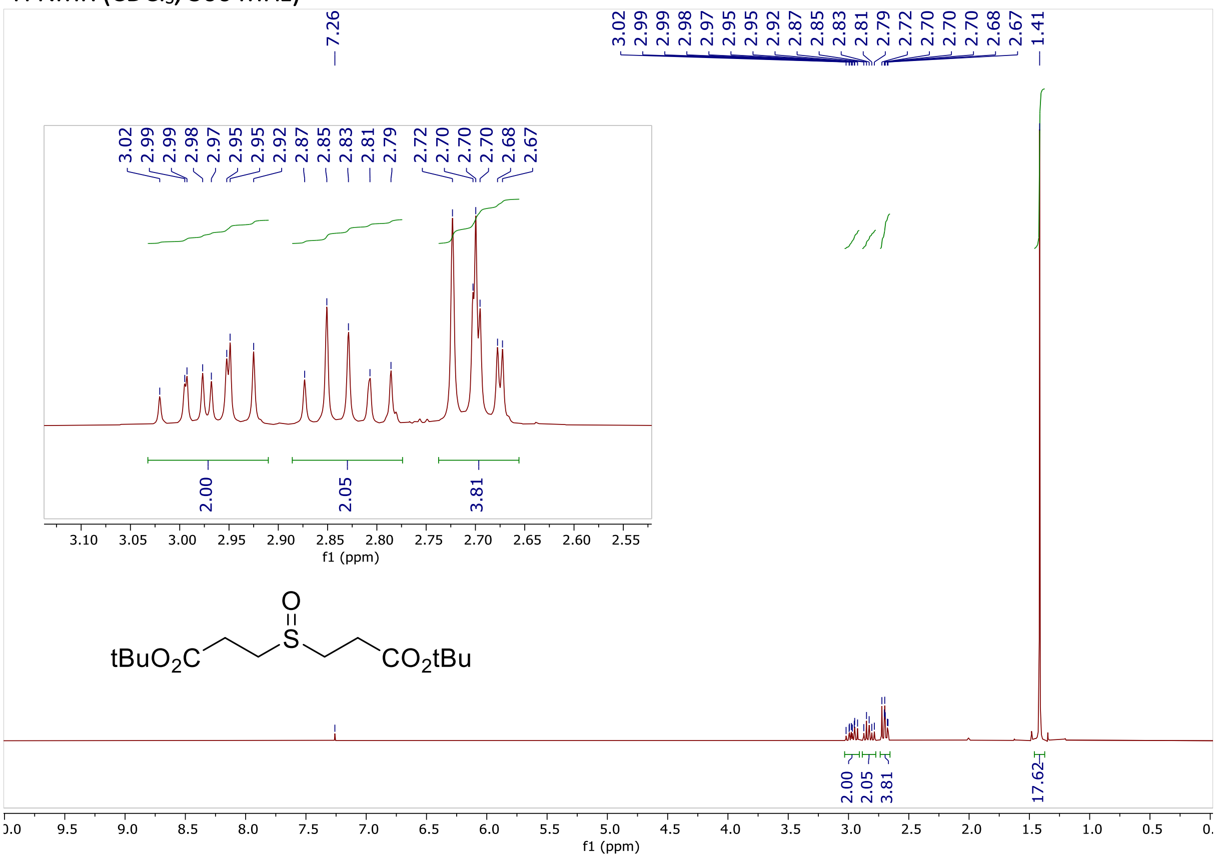

$^{13}C\{^{1}H\}$ NMR ($CDCl_3$, 75 MHz)

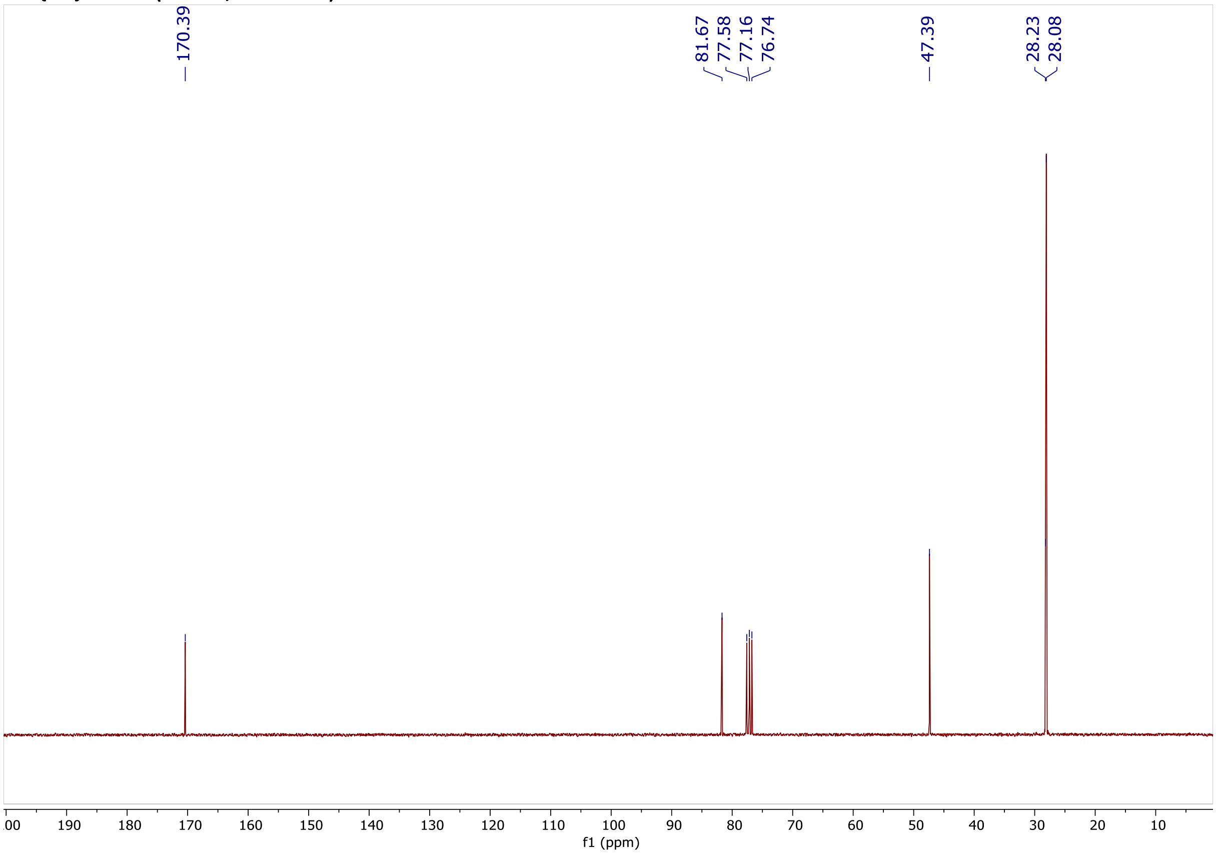

4,4'-sulfinylbis(N,N-diphenylaniline) 4b:

^{1}H NMR ($CDCl_3$, 300 MHz)

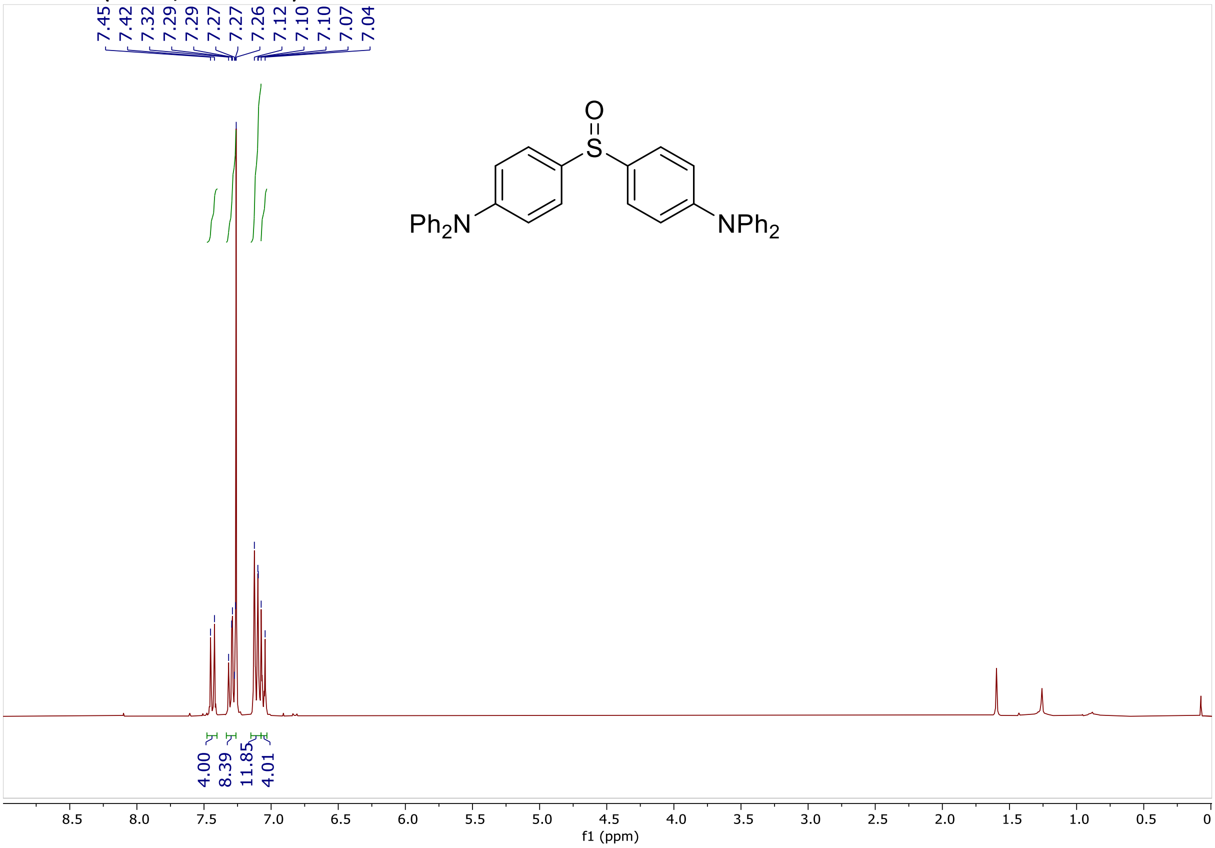

^{13}C{^{1}H} NMR ($CDCl_3$, 75 MHz)

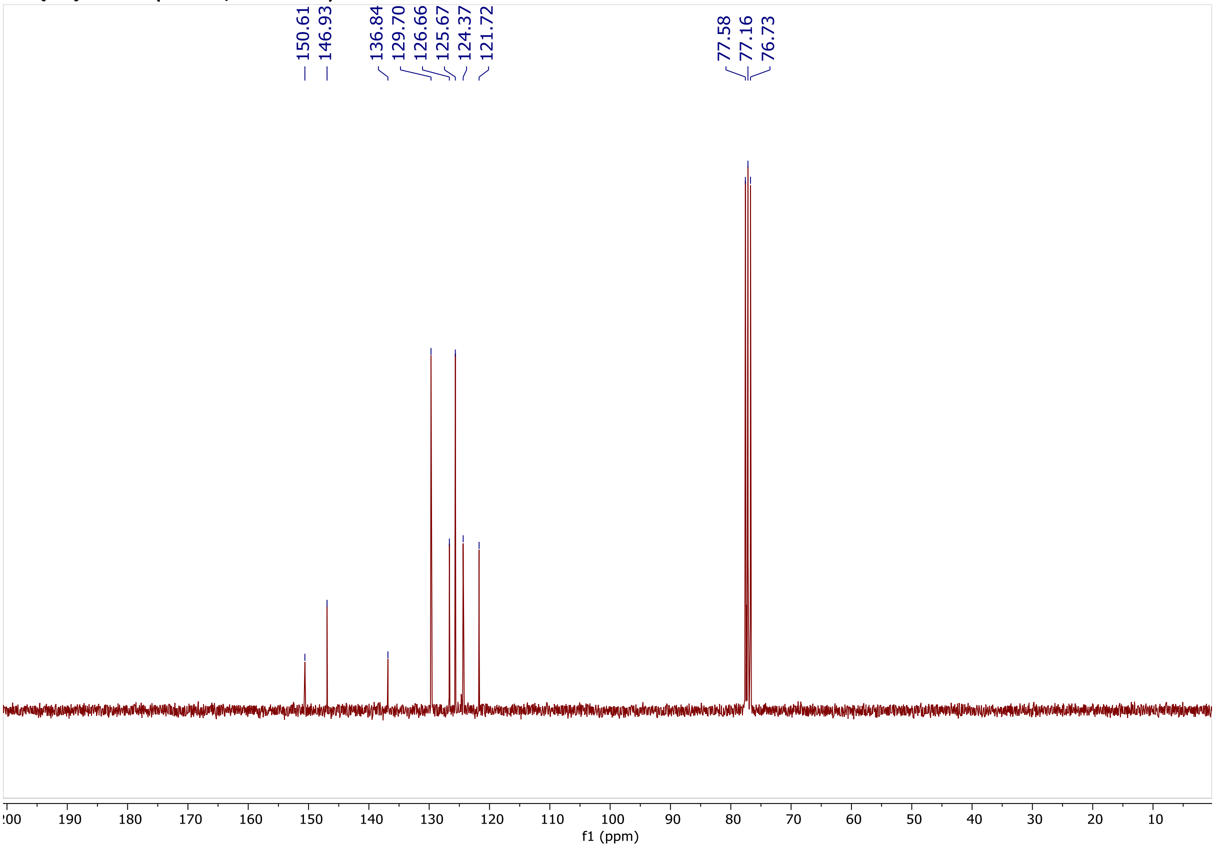

5,5'-sulfinylbis(1,2,3-trimethoxybenzene) 4c:

^{1}H NMR ($CDCl_3$, 300 MHz)

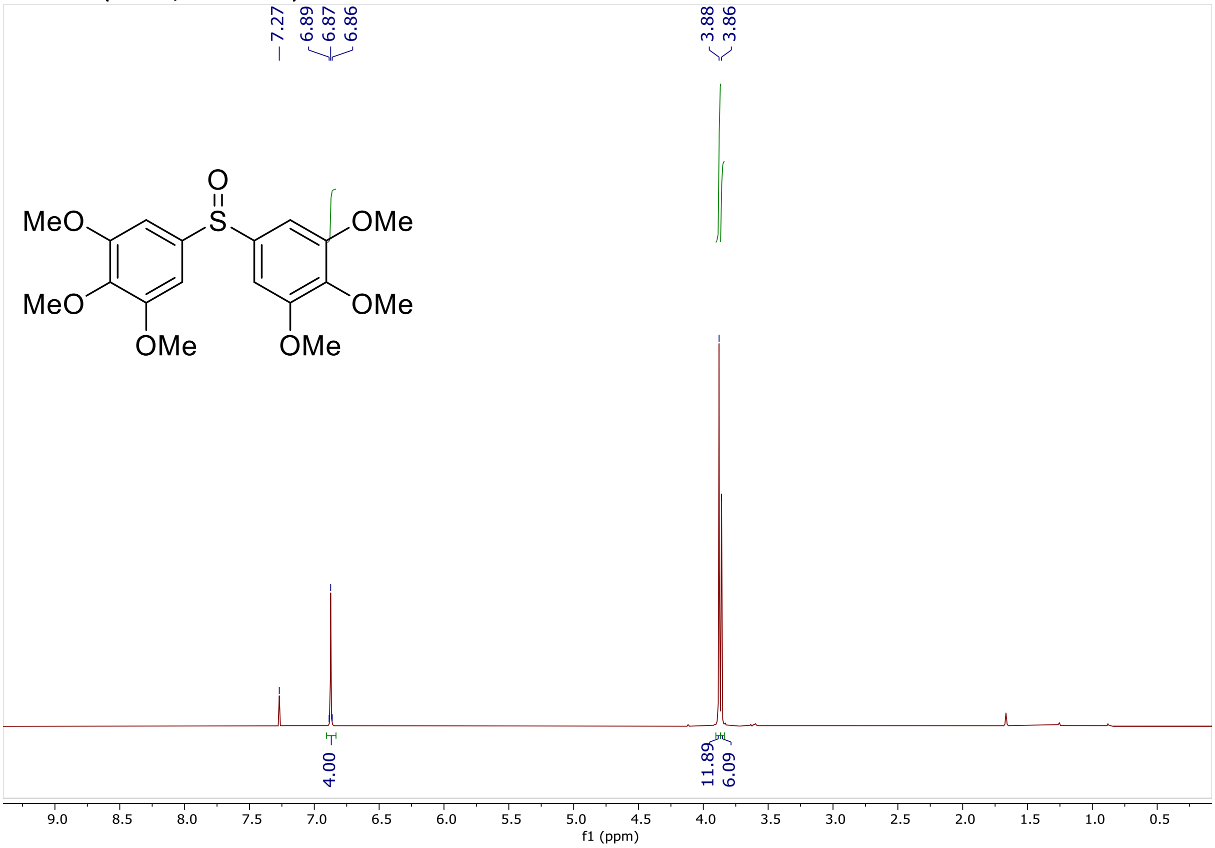

^{13}C{^{1}H} NMR ($CDCl_3$, 75 MHz)

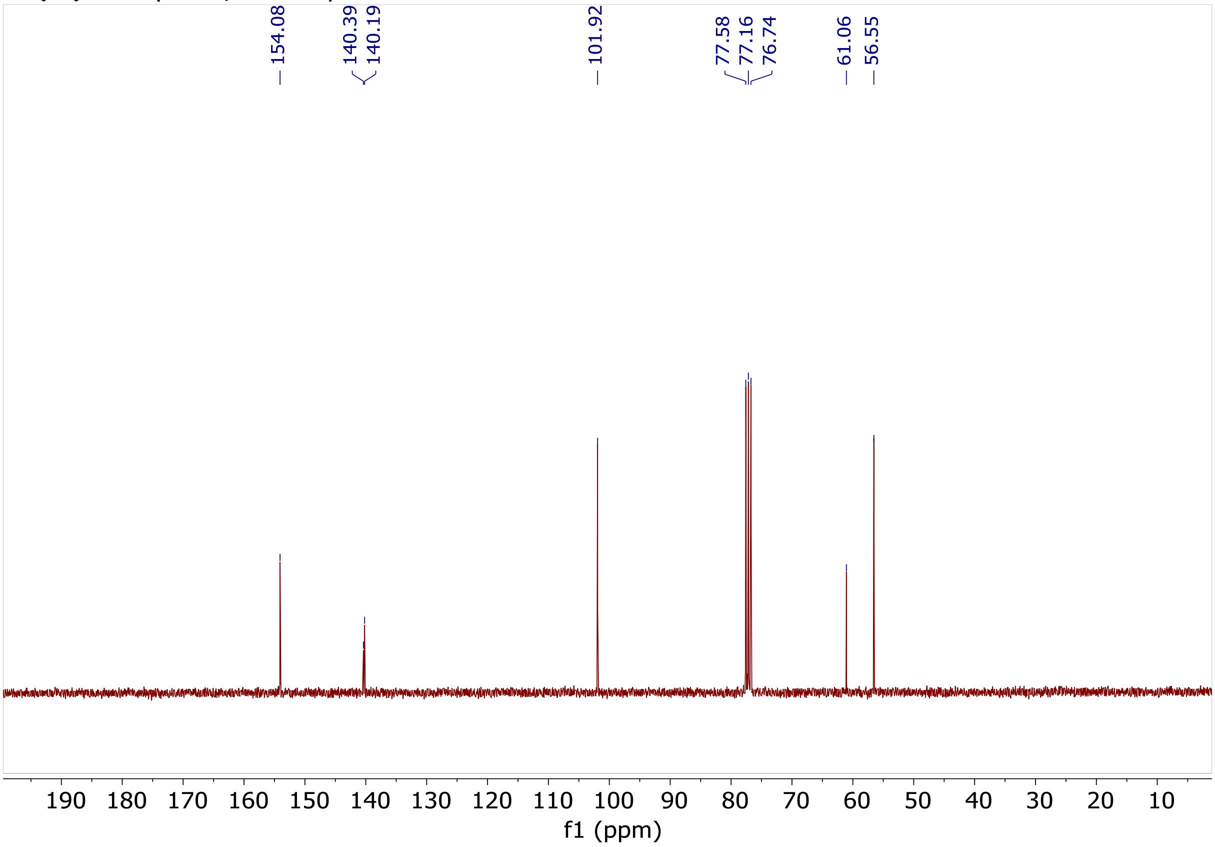

4,4'-sulfinylbis(methoxybenzene) 4d:

^{1}H NMR ($CDCl_3$, 300 MHz)

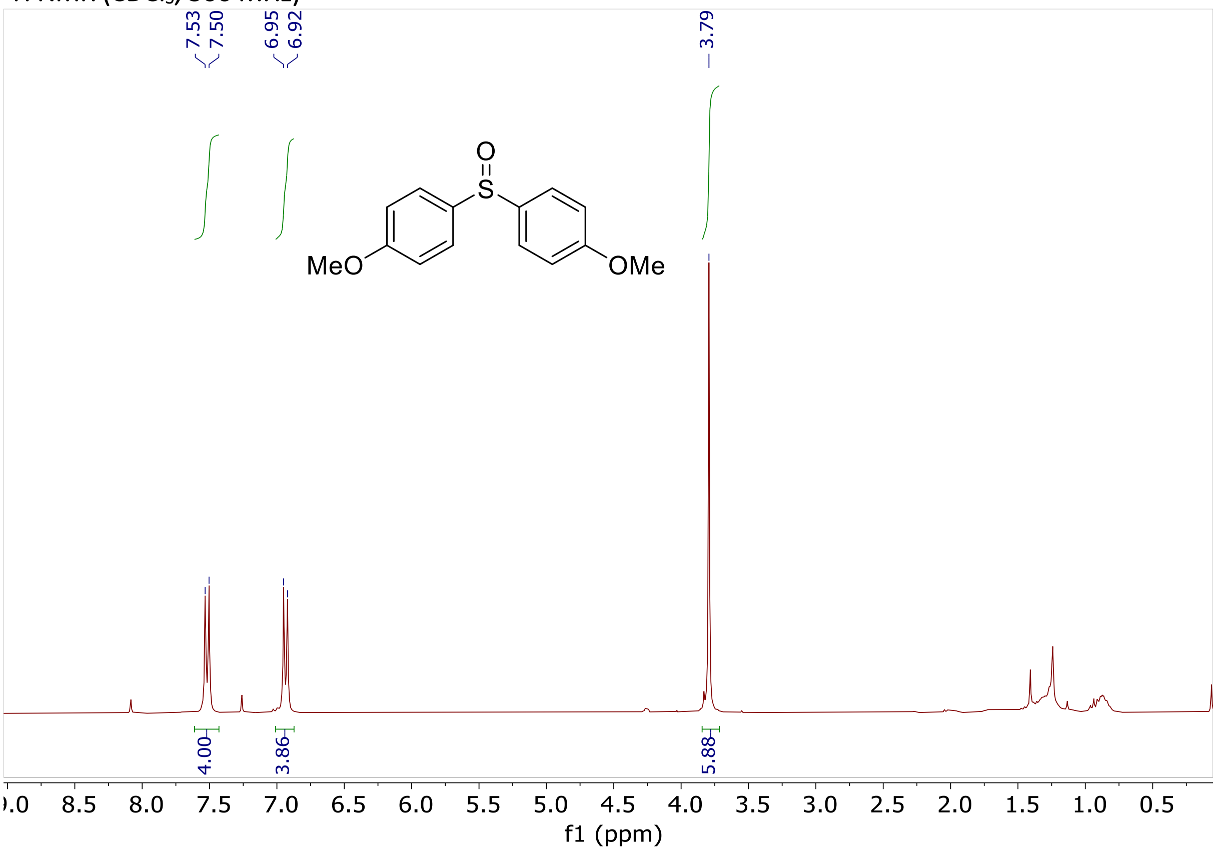

$^{13}C\{^{1}H\}$ NMR ($CDCl_3$, 75 MHz)

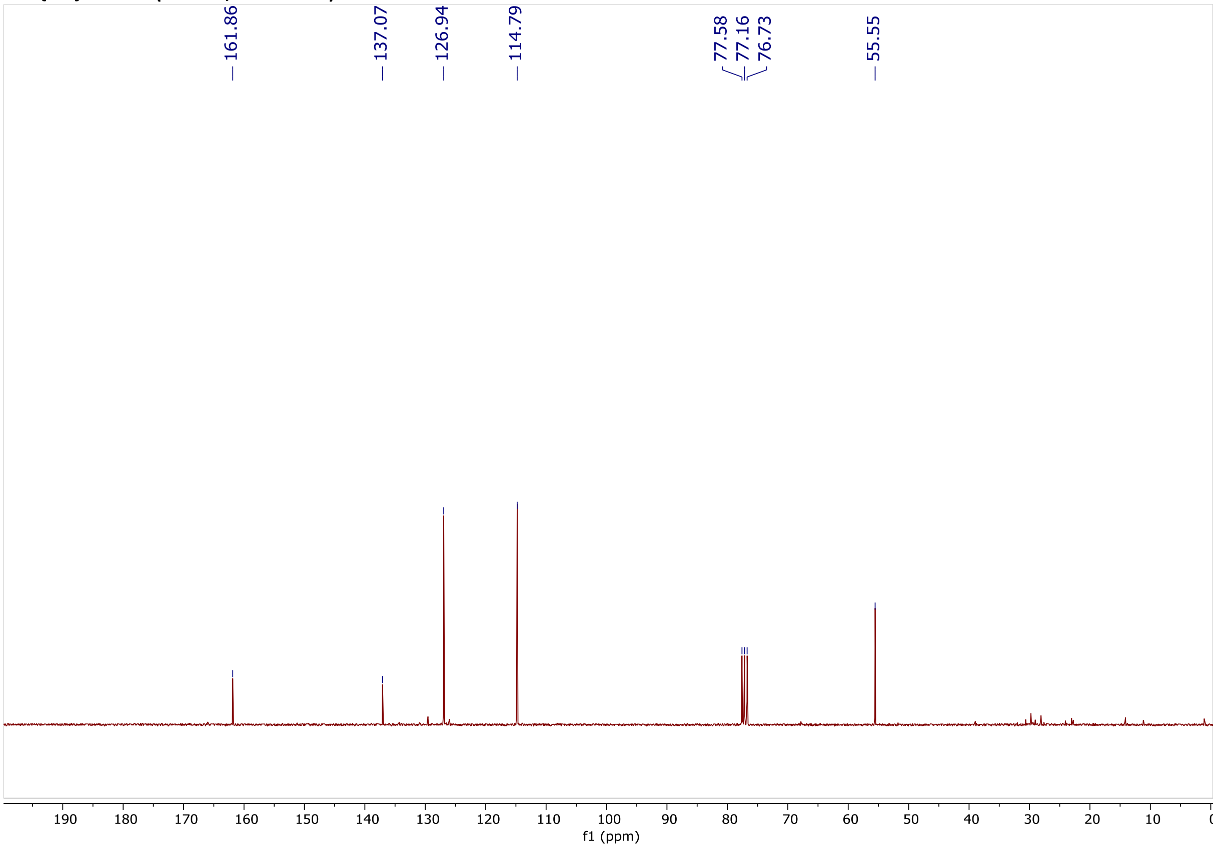

5,5'-sulfinylbis(benzo[d][1,3]dioxole) 4e:

^{1}H NMR ($CDCl_3$, 300 MHz)

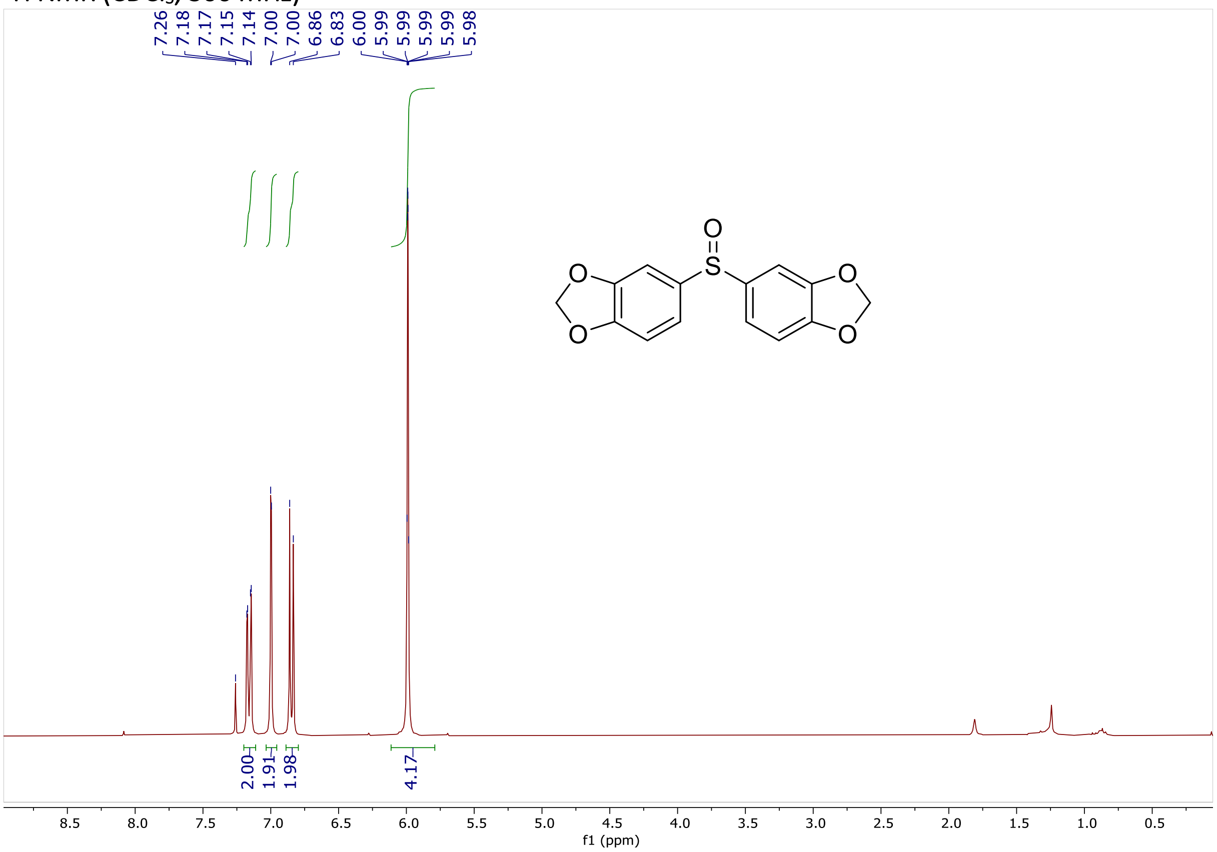

$^{13}C\{^{1}H\}$ NMR ($CDCl_3$, 75 MHz)

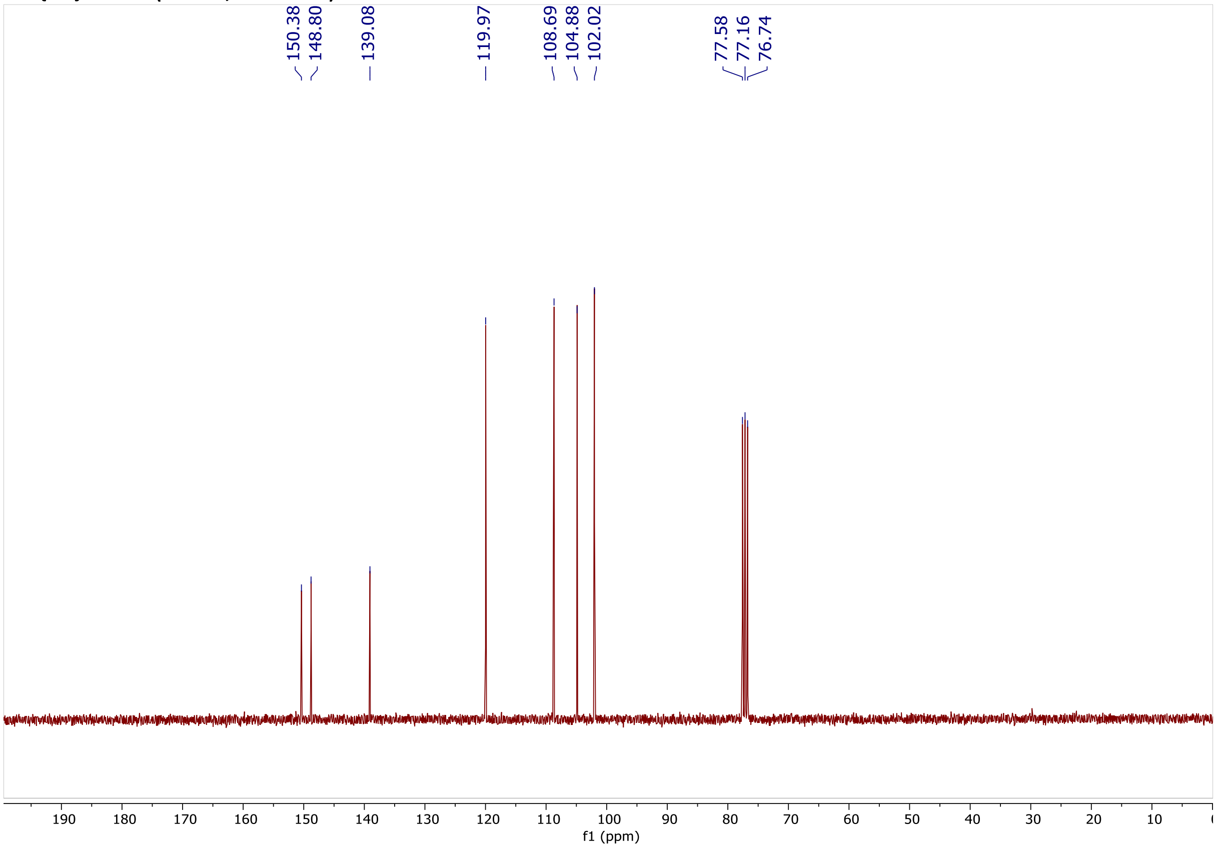

4,4'-sulfinylbis(tert-butylbenzene) 4f:

^{1}H NMR ($CDCl_3$, 300 MHz)

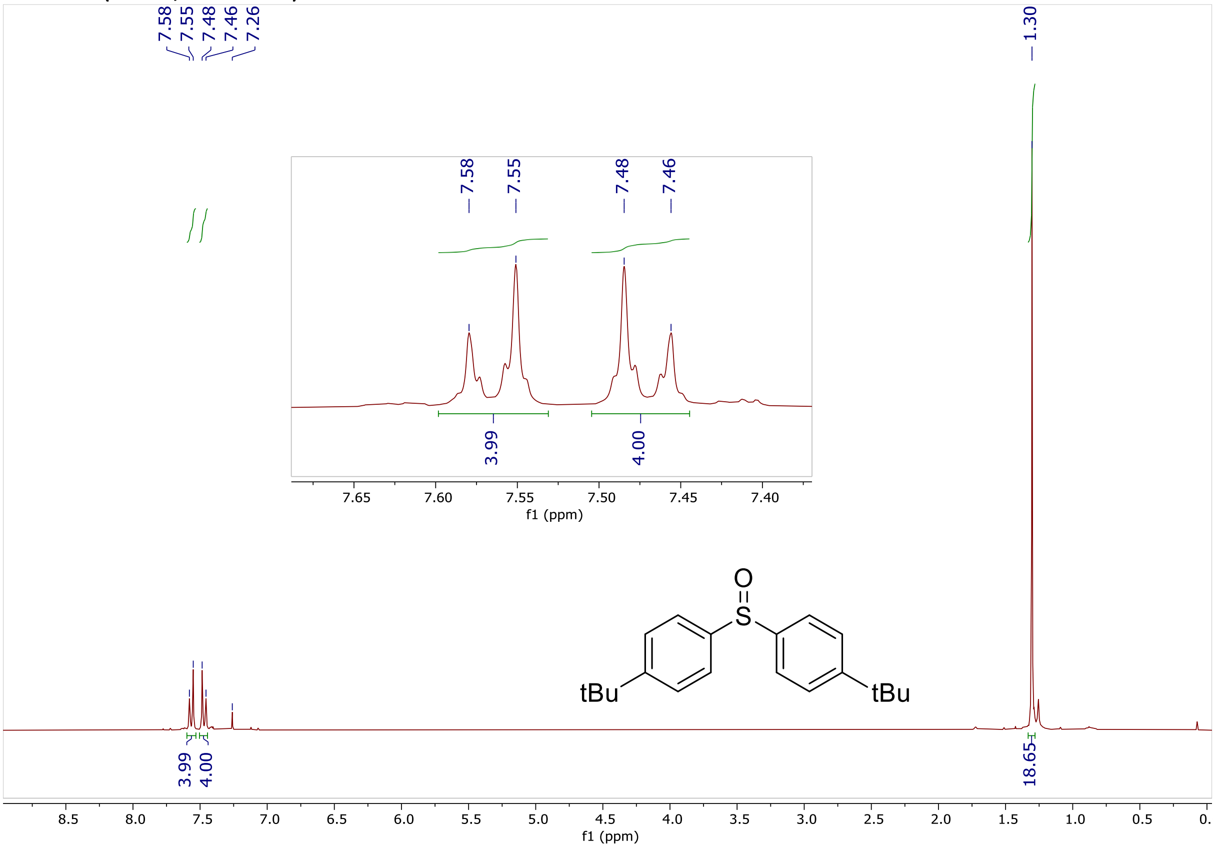

$^{13}C\{^{1}H\}$ NMR ($CDCl_3$, 75 MHz)

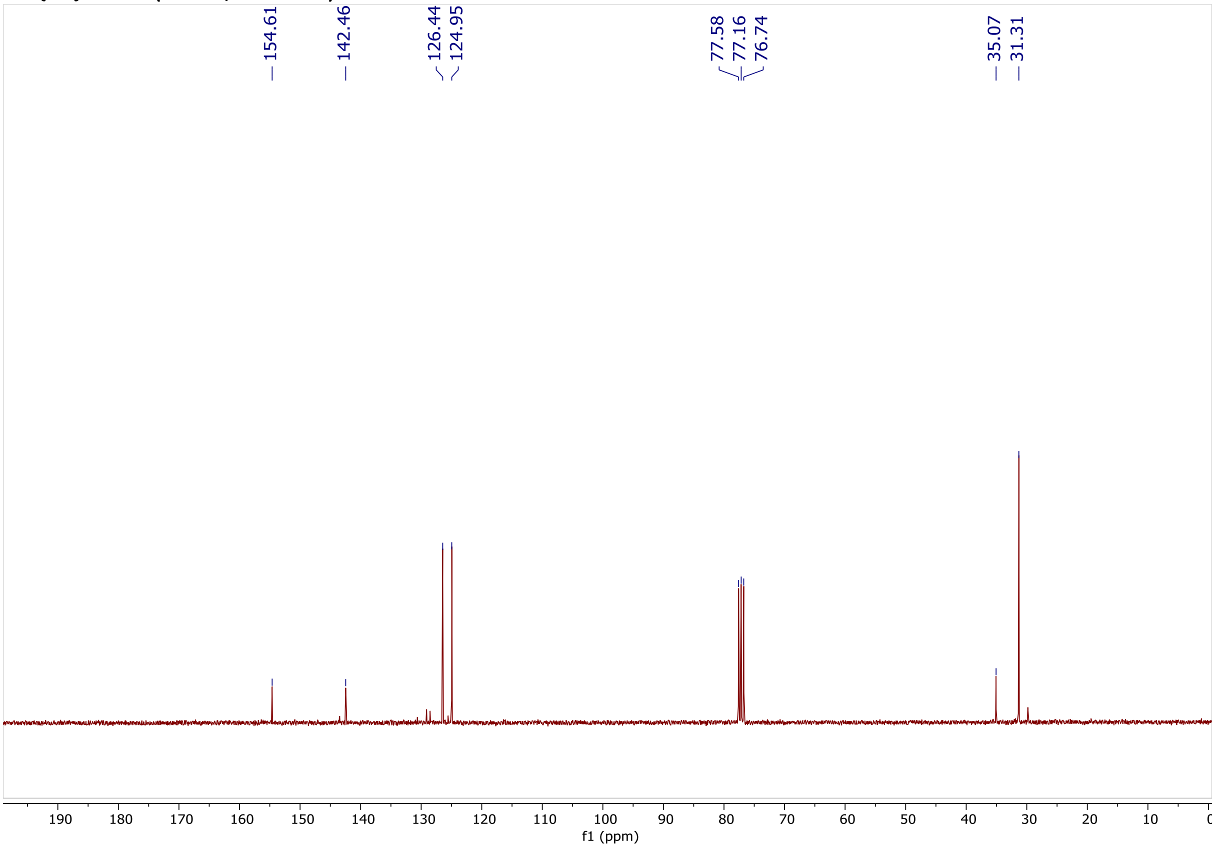

4,4'-sulfinylbis(methylbenzene) 4a:

^1H NMR ($CDCl_3$, 300 MHz)

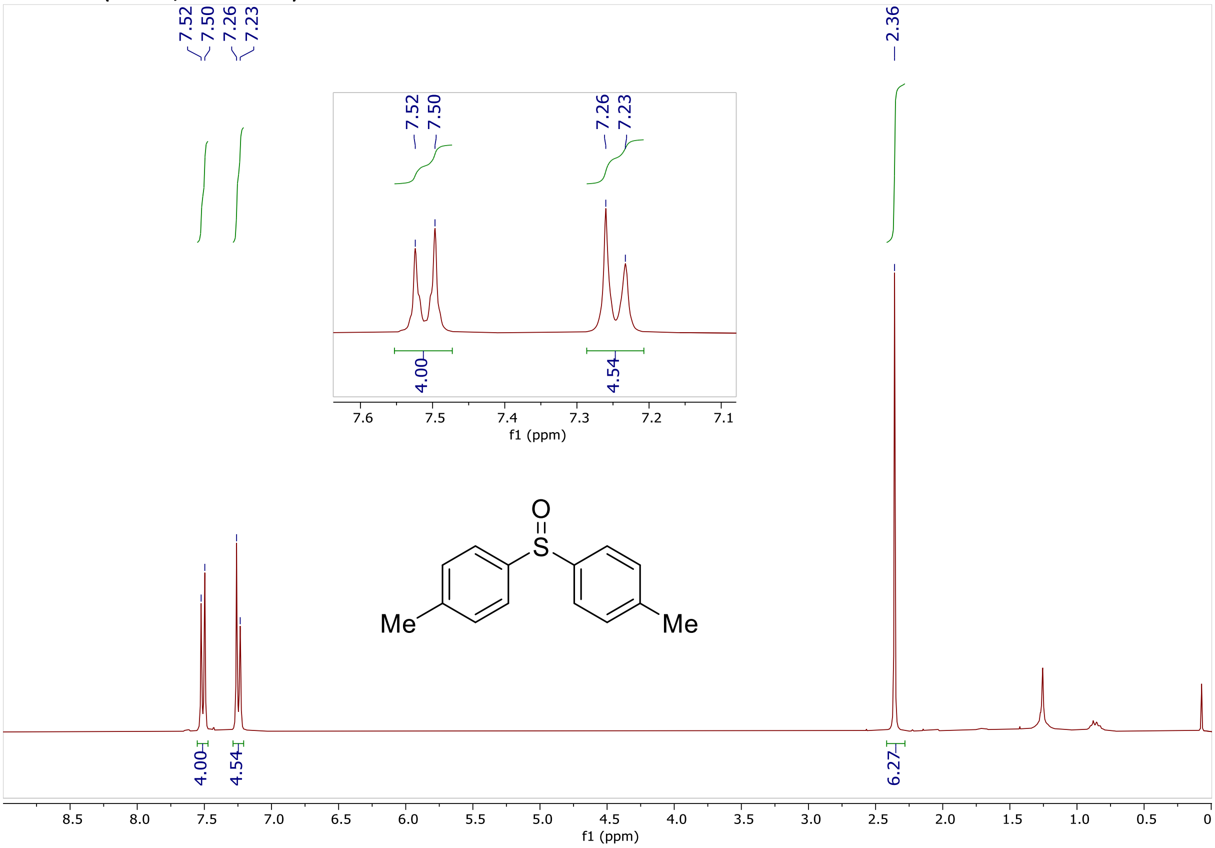

$^{13}C\{^1H\}$ NMR ($CDCl_3$, 75 MHz)

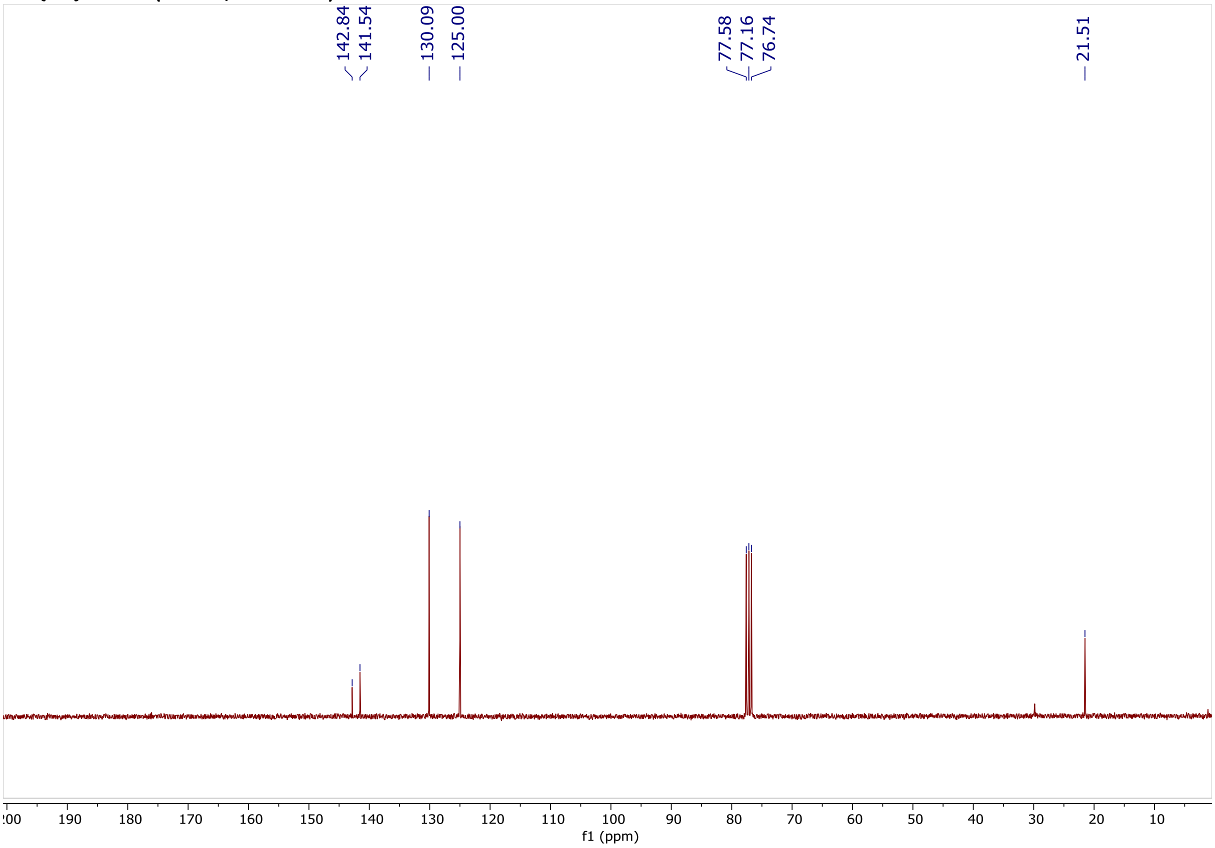

3,3'-sulfinylbis(methylbenzene) 4g:

^{1}H NMR ($CDCl_3$, 300 MHz)

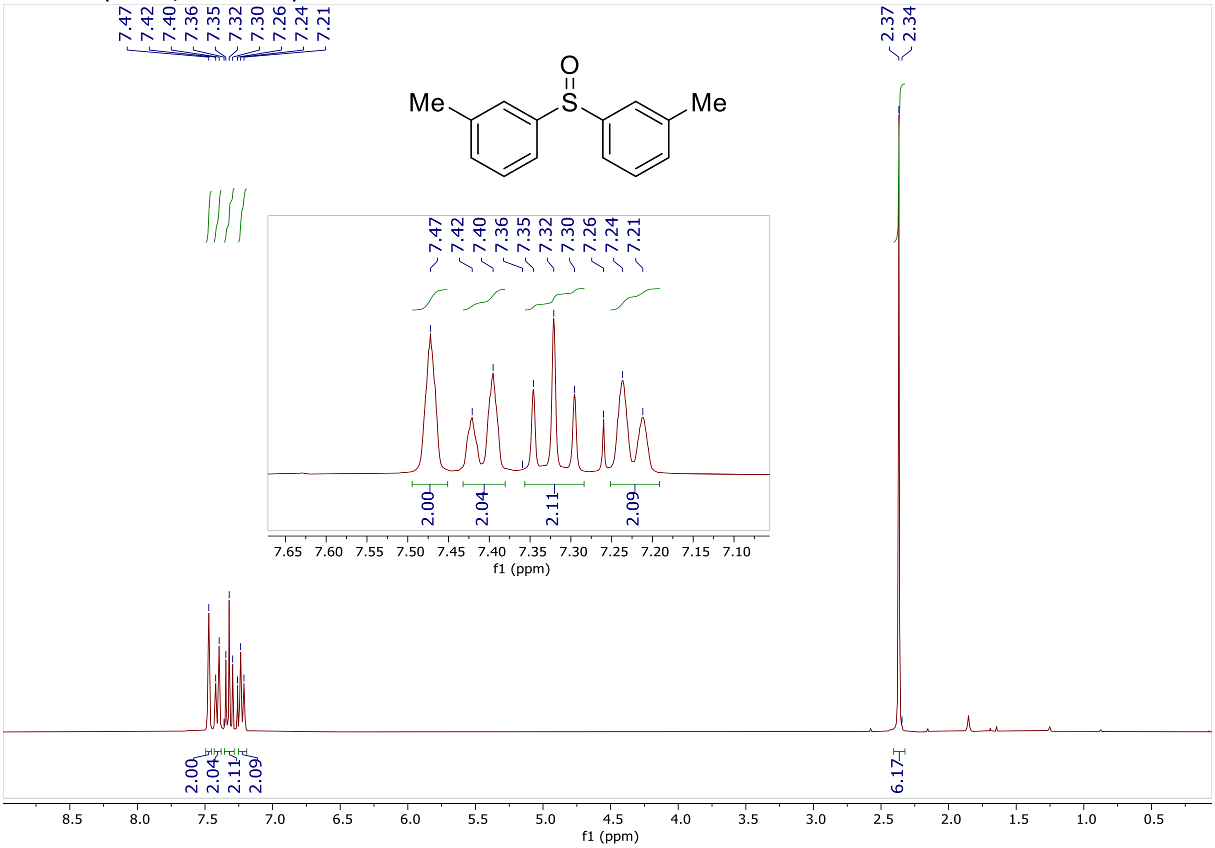

^{13}C{^{1}H} NMR ($CDCl_3$, 75 MHz)

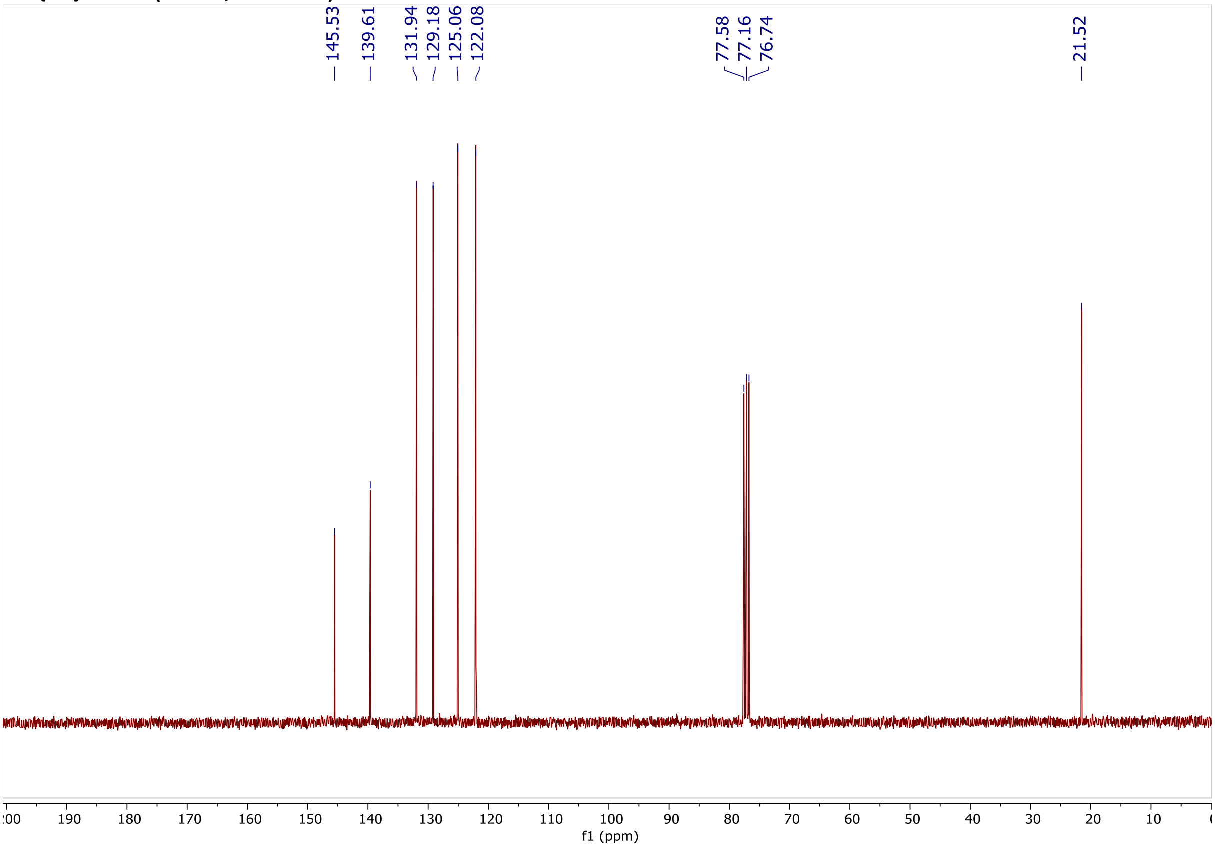

(sulfinylbis(4,1-phenylene))bis(methylsulfane) 4h:

^{1}H NMR ($CDCl_3$, 300 MHz)

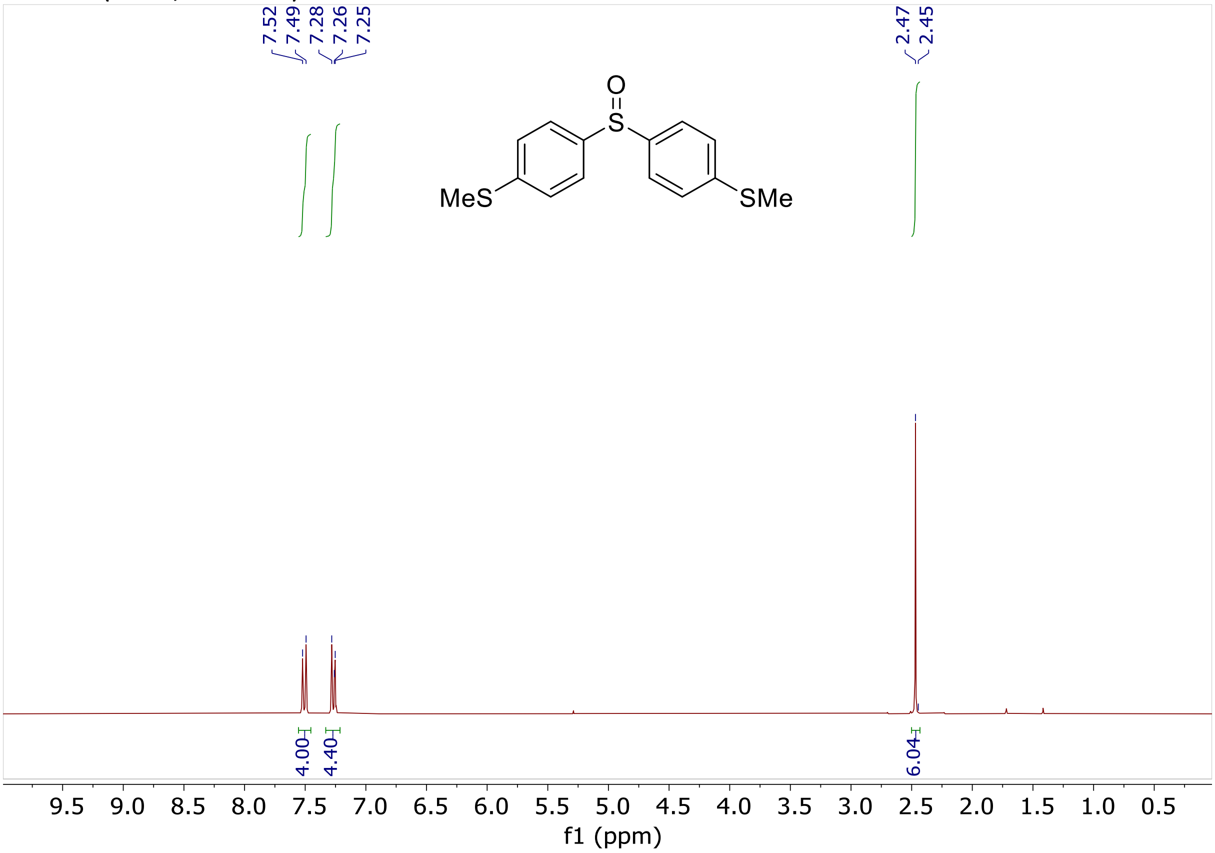

$^{13}C\{^{1}H\}$ NMR ($CDCl_3$, 75 MHz)

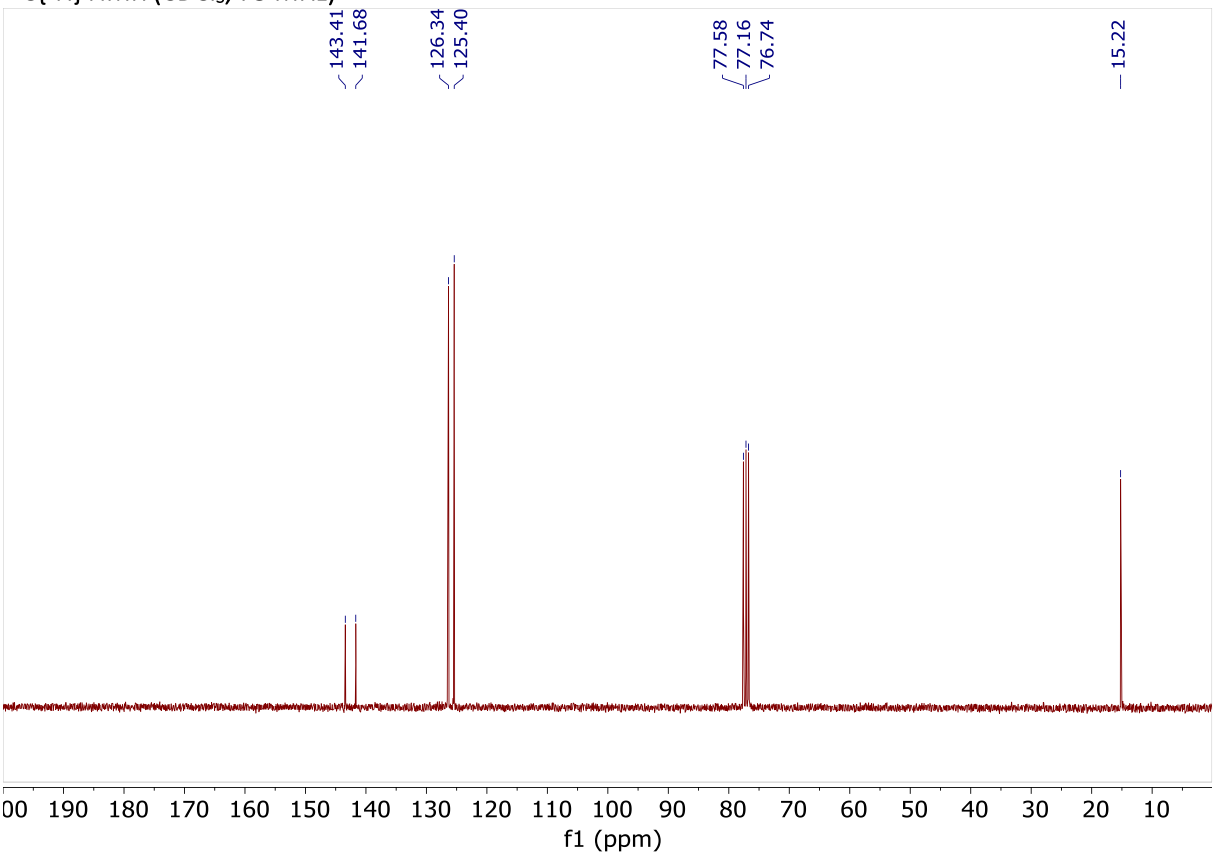

Sulfinyldibenzene 4i:

^{1}H NMR ($CDCl_3$, 300 MHz)

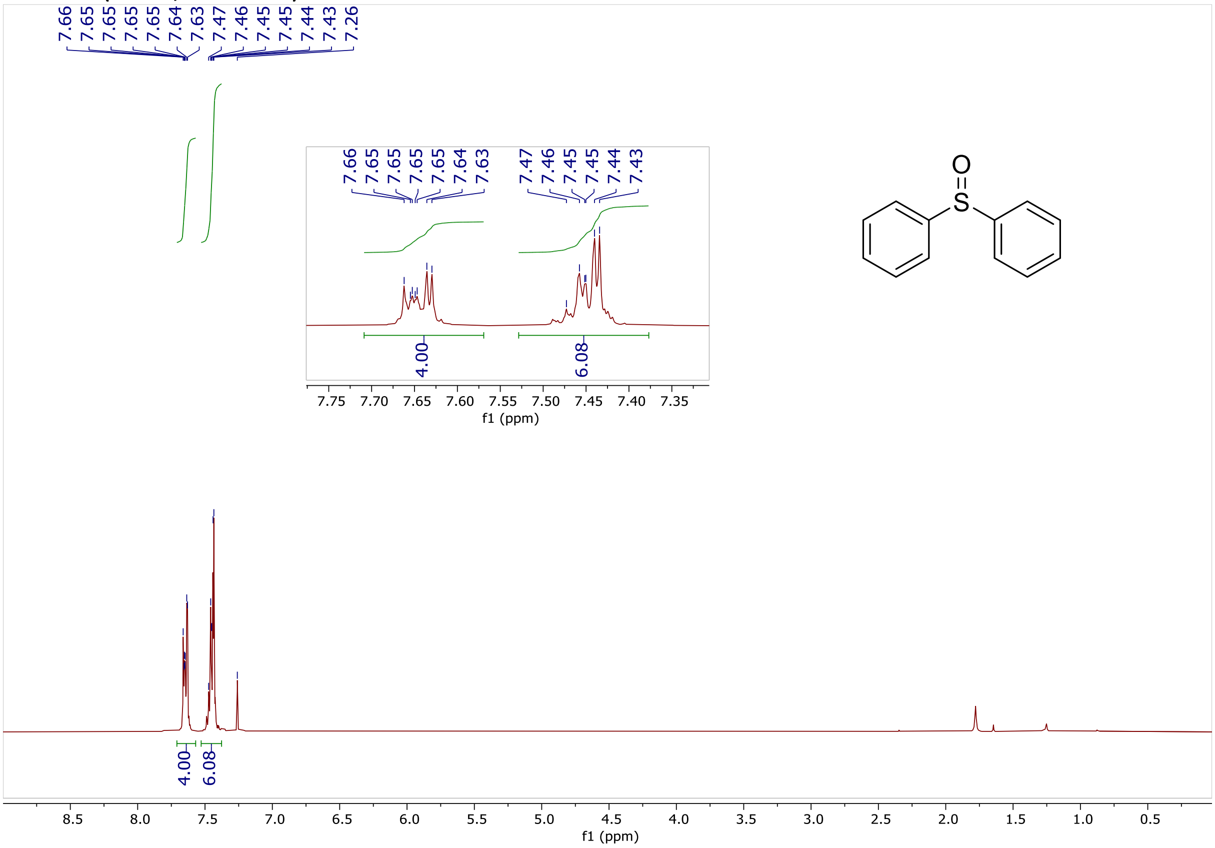

^{13}C{^{1}H} NMR ($CDCl_3$, 75 MHz)

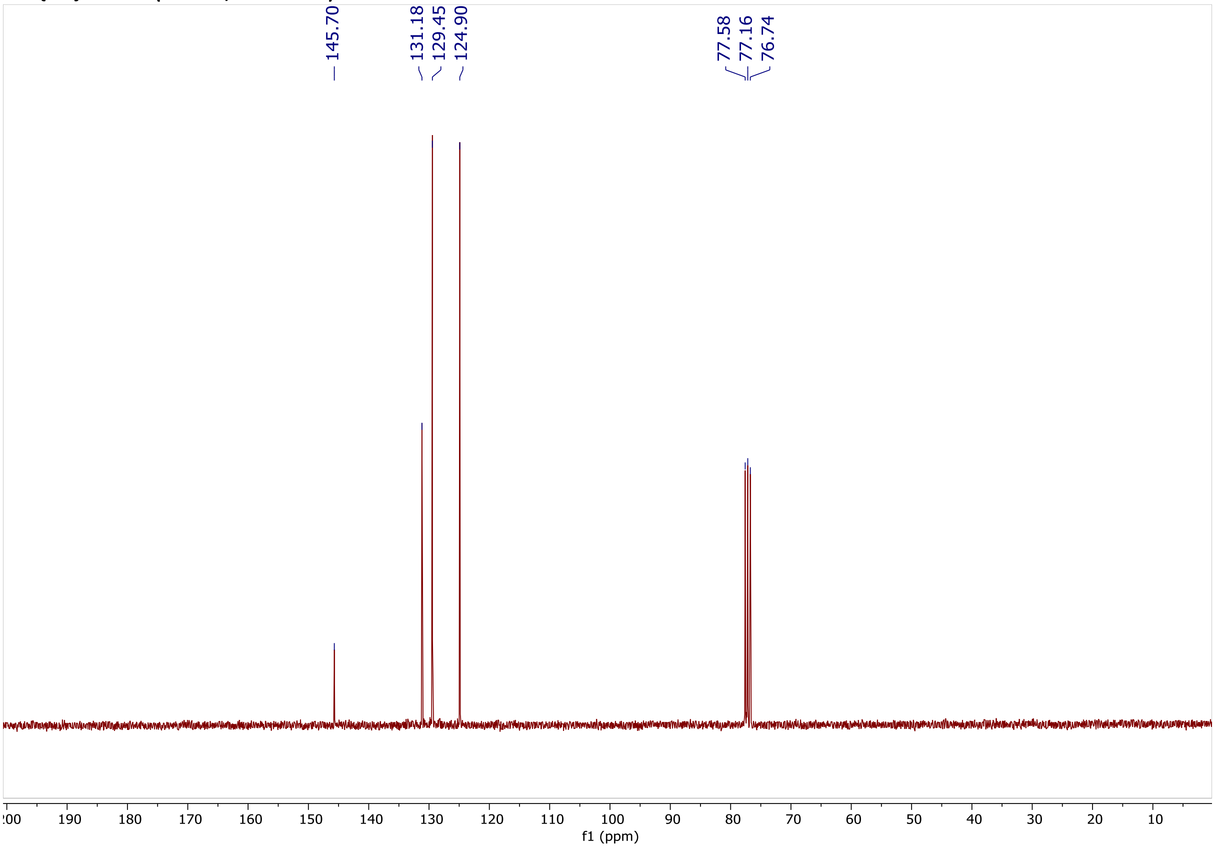

3,3'-sulfinylbis(methoxybenzene) 4j:

^{1}H NMR ($CDCl_3$, 300 MHz)

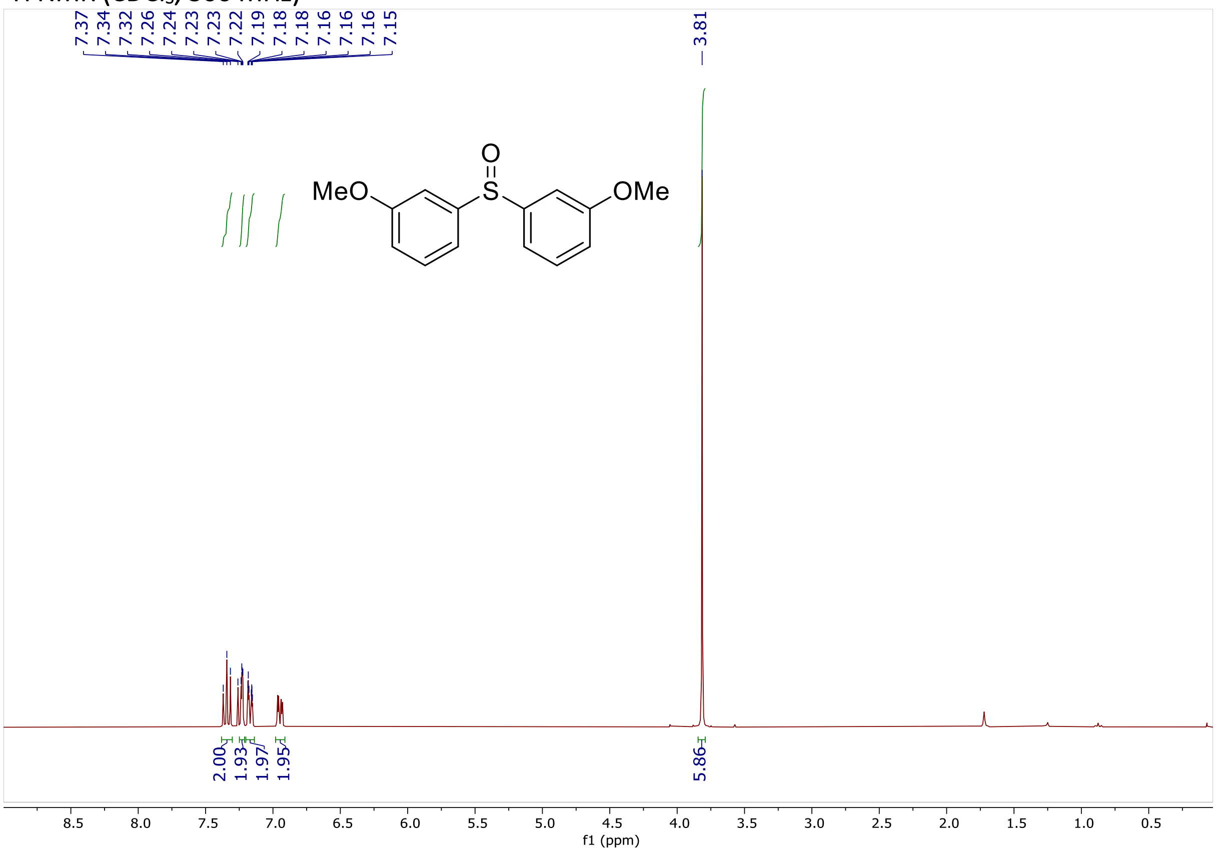

$^{13}C\{^{1}H\}$ NMR ($CDCl_3$, 75 MHz)

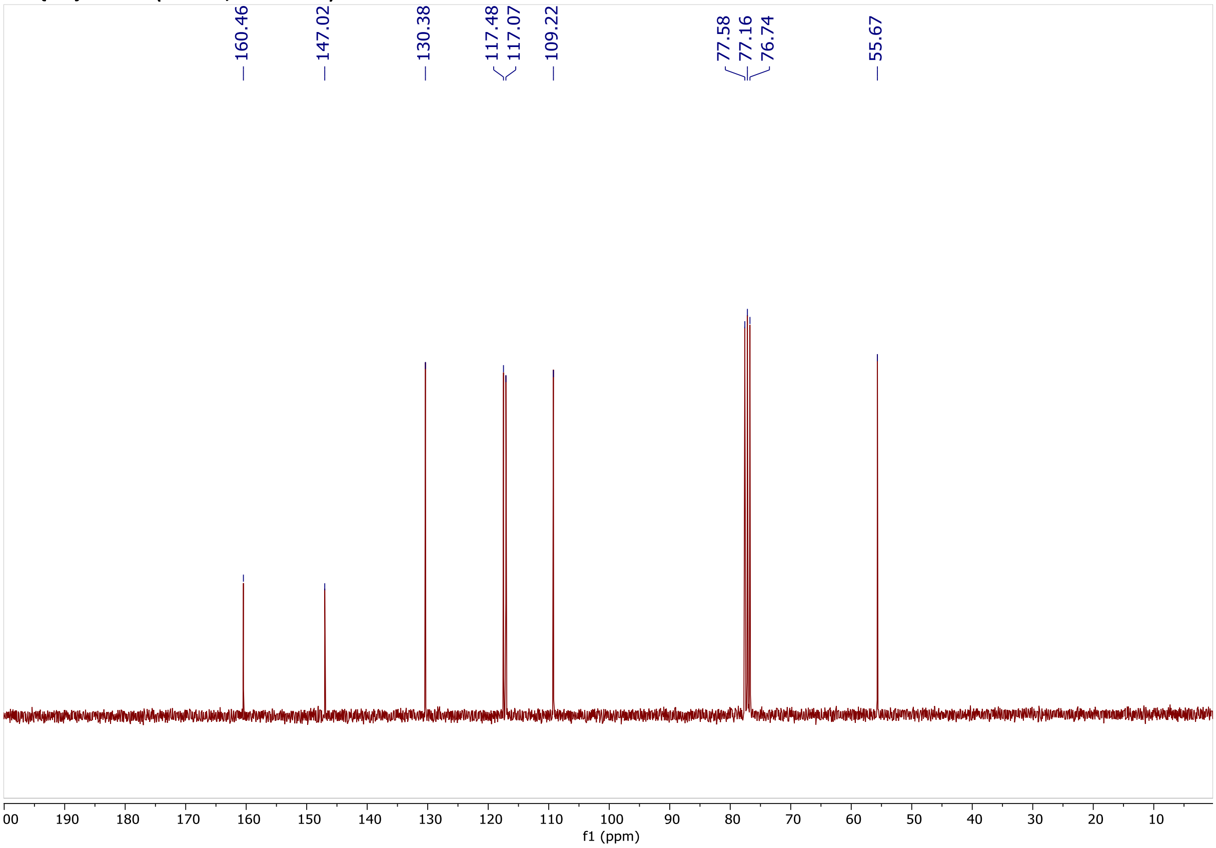

4,4'-sulfinylbis(fluorobenzene) 4k:

^{1}H NMR ($CDCl_3$, 300 MHz)

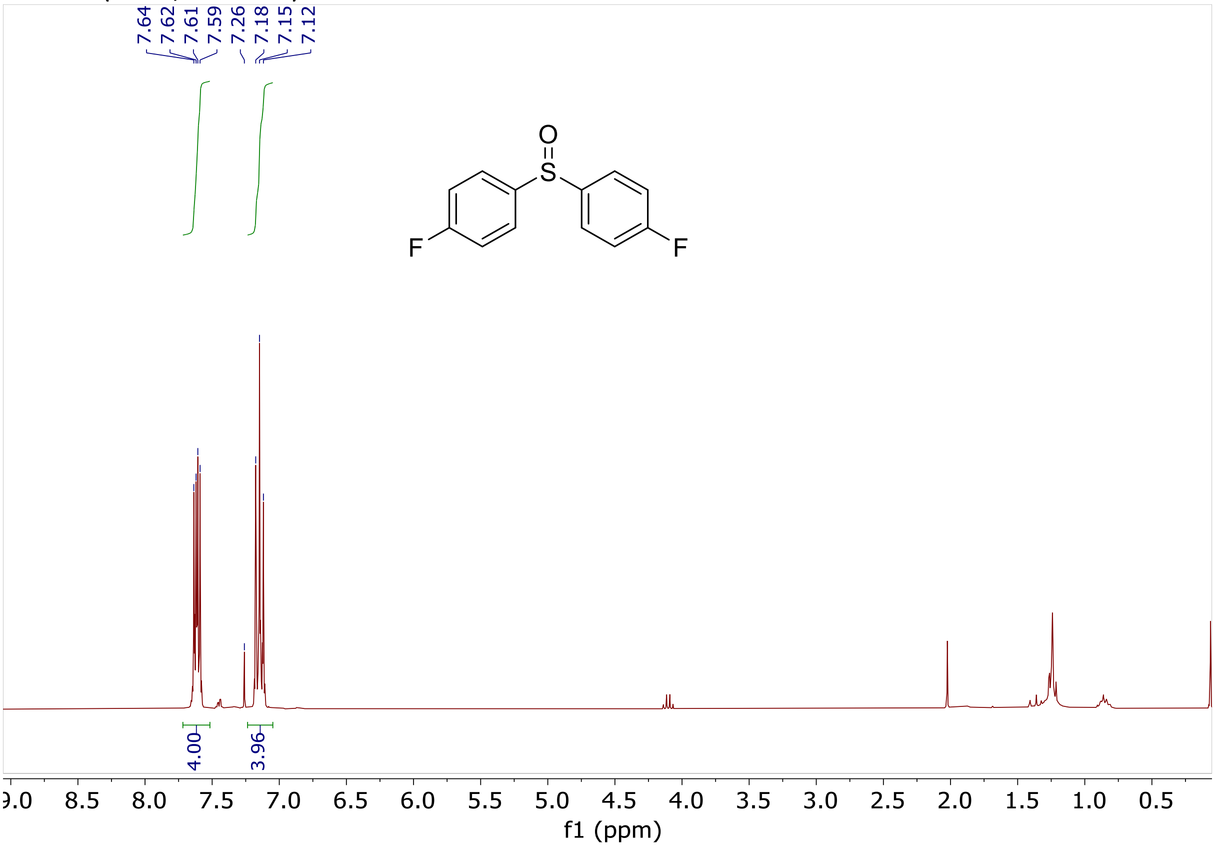

^{13}C{^{1}H} NMR ($CDCl_3$, 75 MHz)

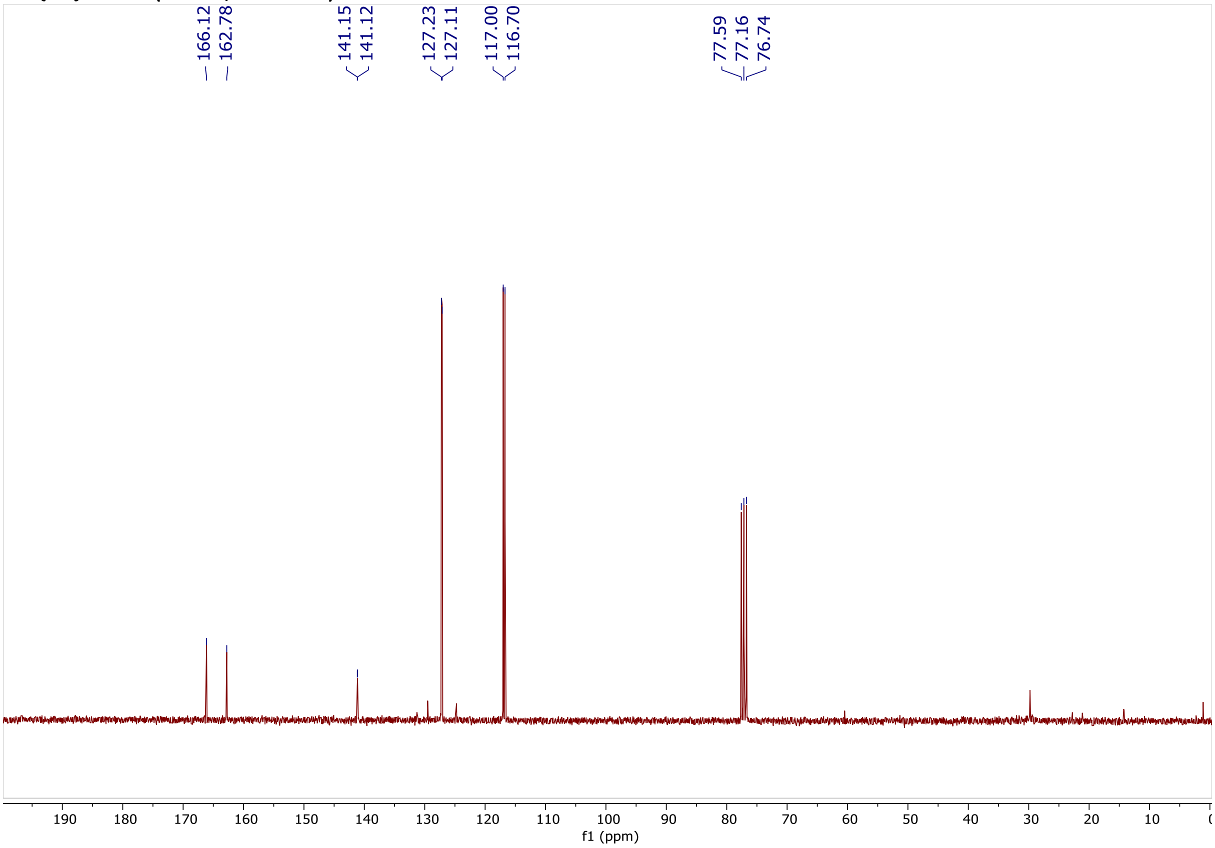

4,4'-sulfinylbis(bromobenzene) 4l:

^{1}H NMR ($CDCl_3$, 300 MHz)

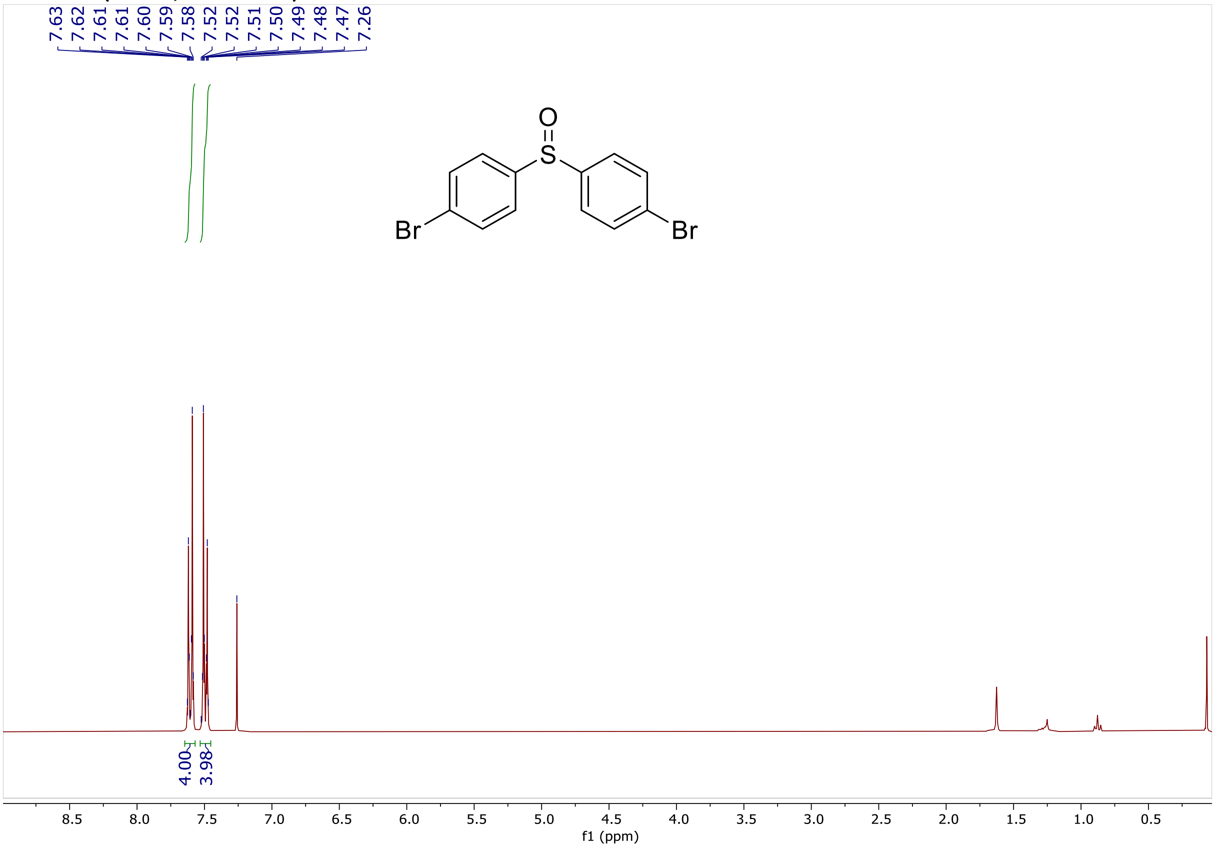

$^{13}C\{^{1}H\}$ NMR ($CDCl_3$, 75 MHz)

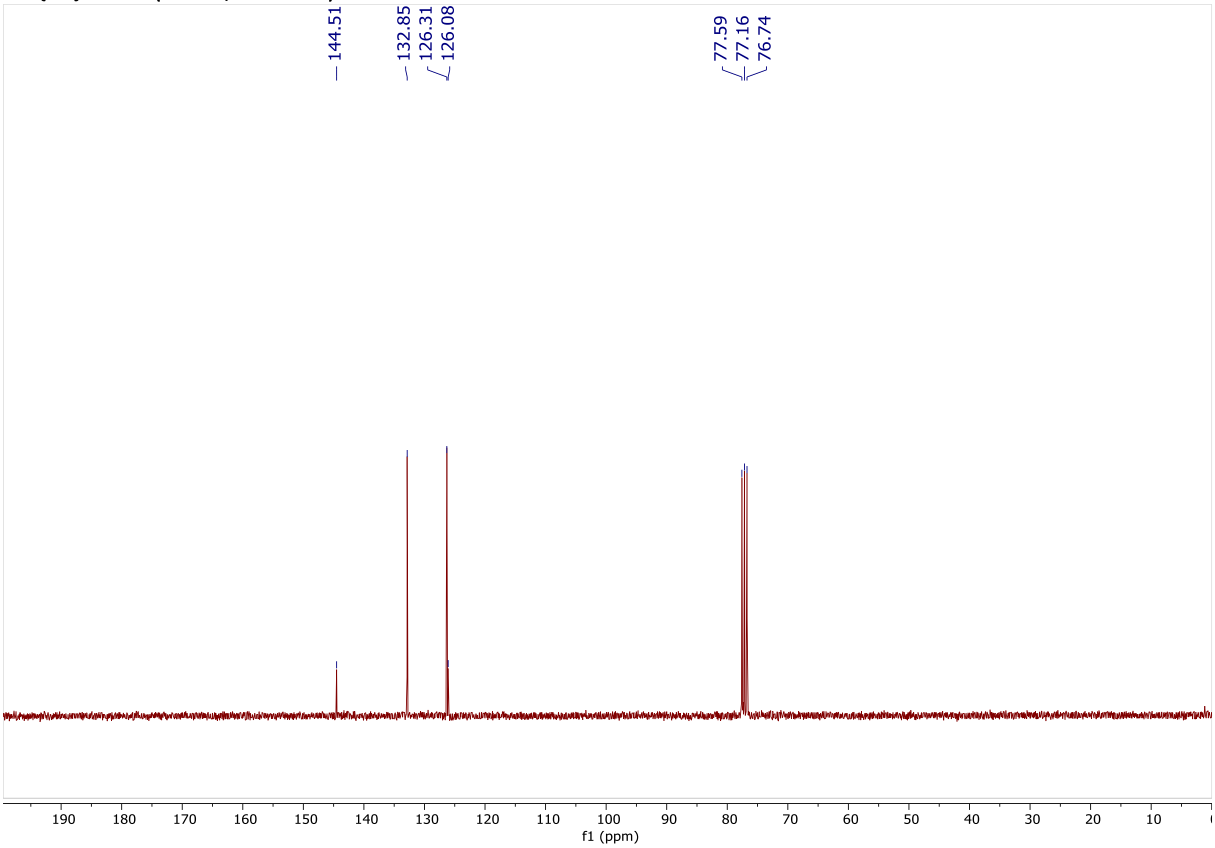

1,1'-(sulfinylbis(4,1-phenylene))bis(ethan-1-one) 4m:

^{1}H NMR ($CDCl_3$, 300 MHz)

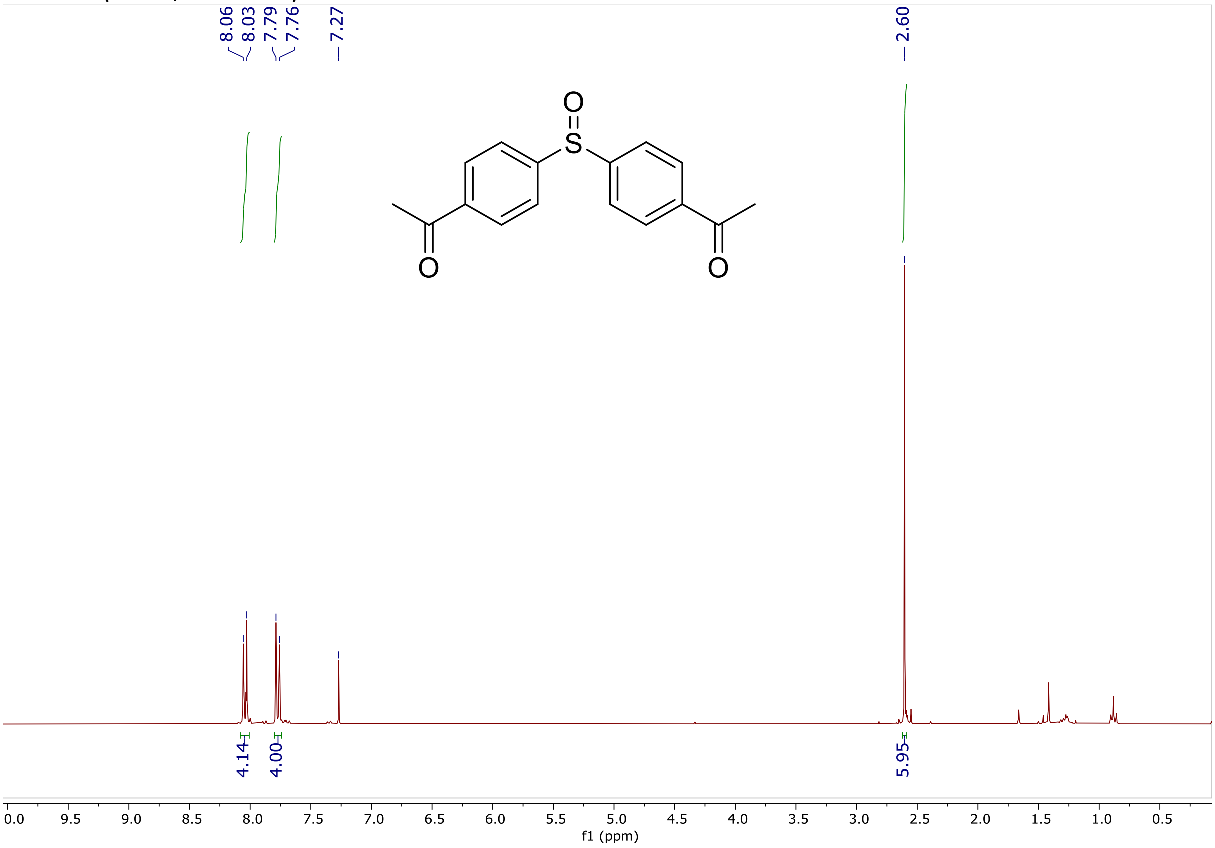

^{13}C{^{1}H} NMR ($CDCl_3$, 75 MHz)

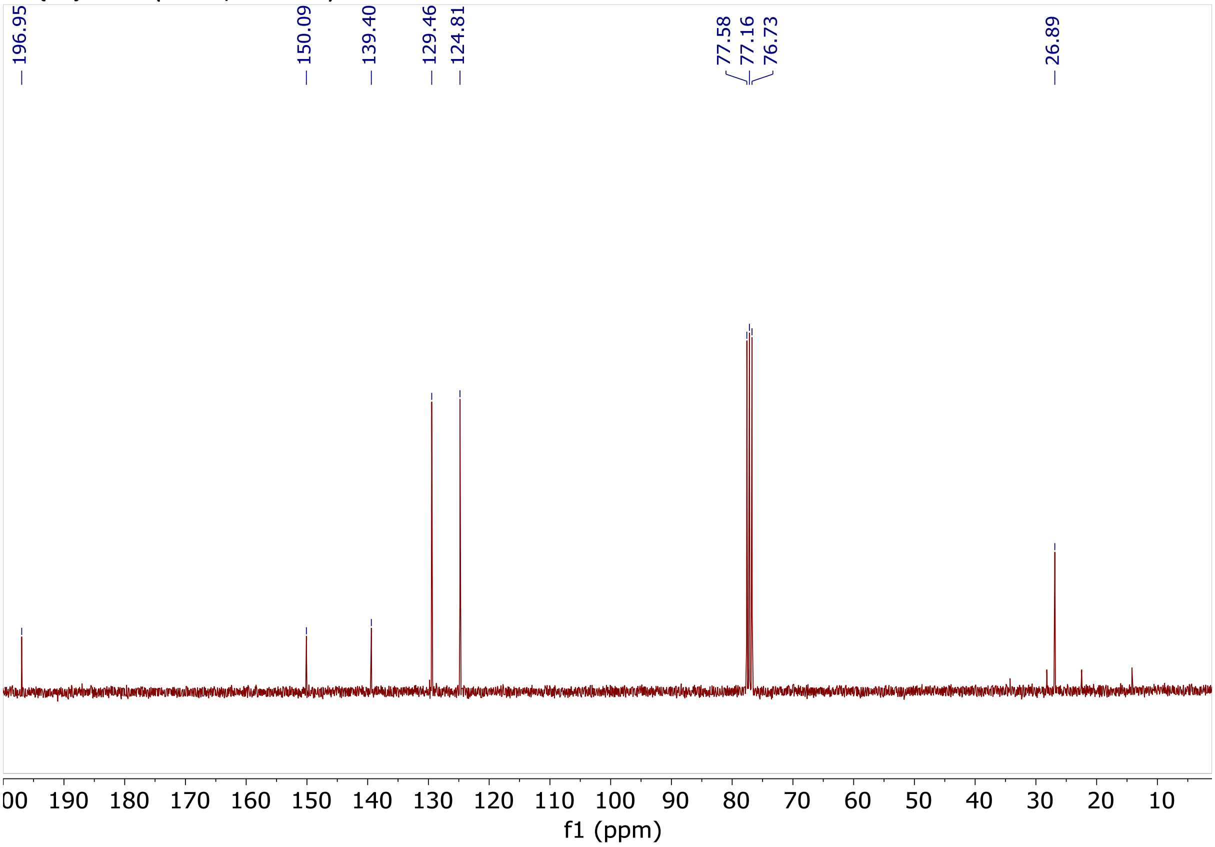

5,5'-sulfinylbis(1,3-bis(trifluoromethyl)benzene) 4n:

^{1}H NMR ($CDCl_3$, 600 MHz)

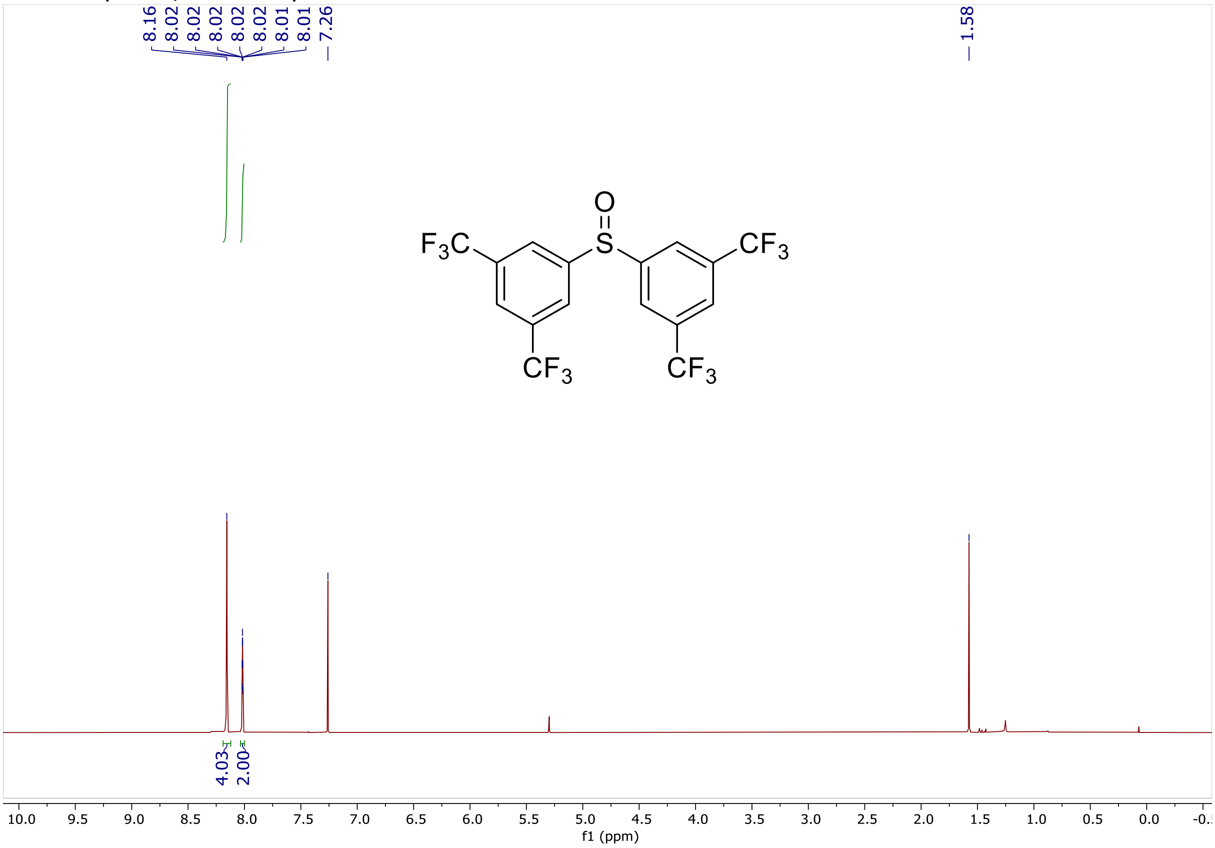

^{13}C{^{1}H} NMR ($CDCl_3$, 151 MHz)

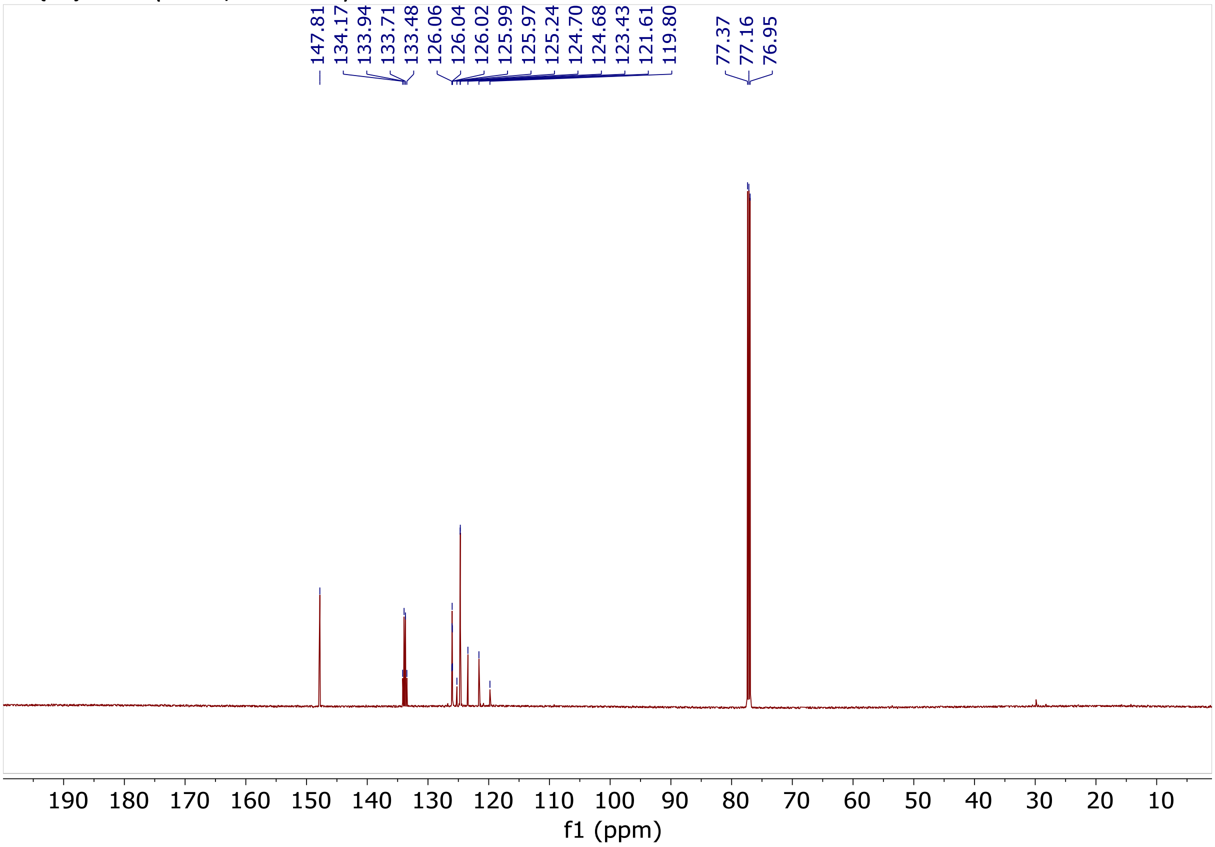

(sulfinylbis(4,1-phenylene))bis(pentafluoro-λ^6-sulfane) 4o:

^{1}H NMR ($CDCl_3$, 300 MHz)

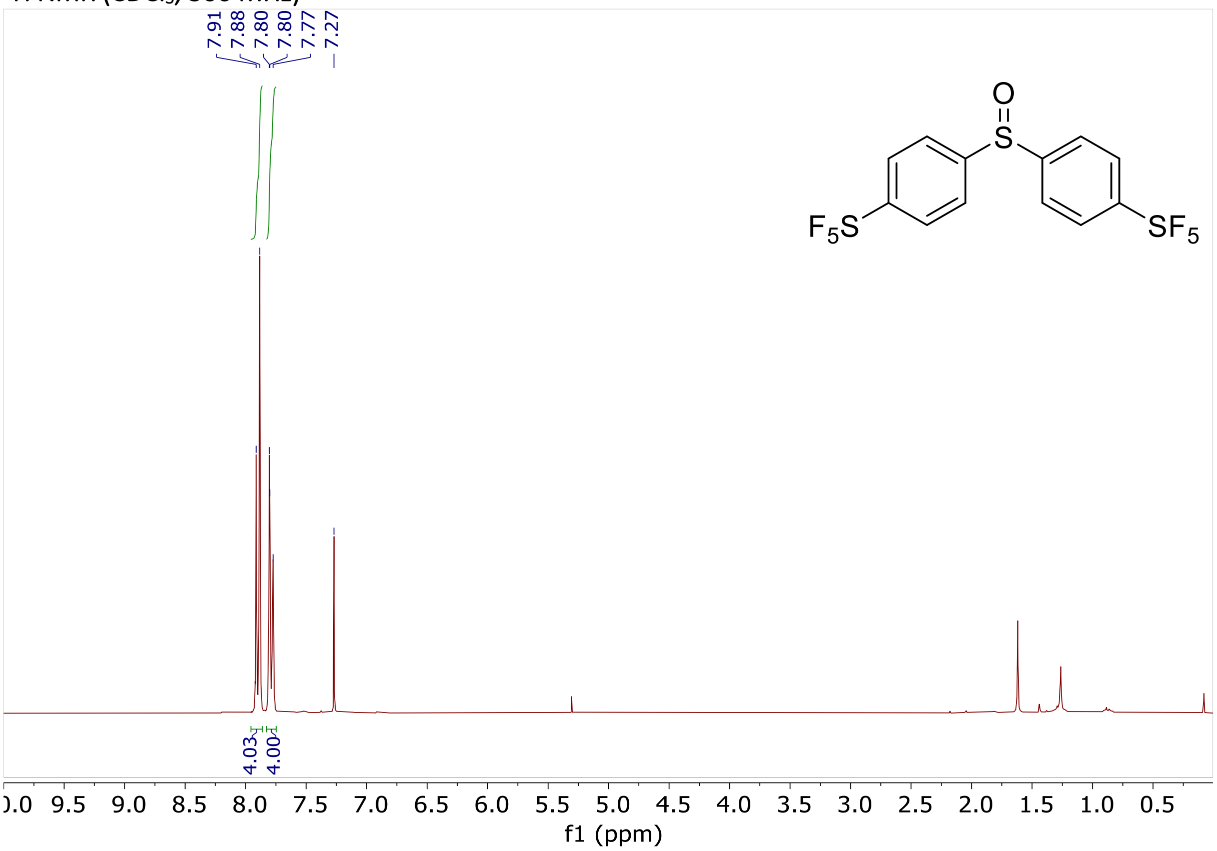

^{13}C{^{1}H} NMR ($CDCl_3$, 151 MHz)

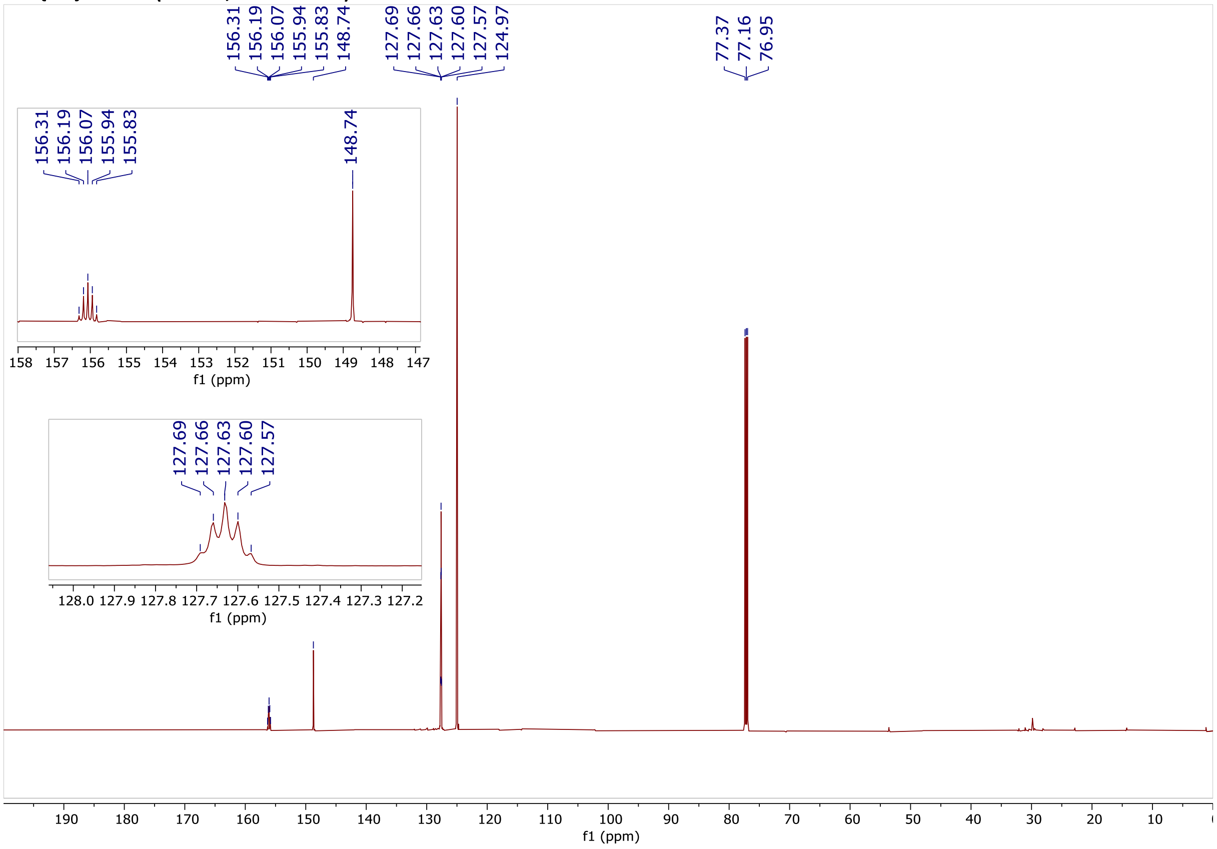

$^{19}F\{^{1}H\}$ NMR ($CDCl_3$, 282 MHz)

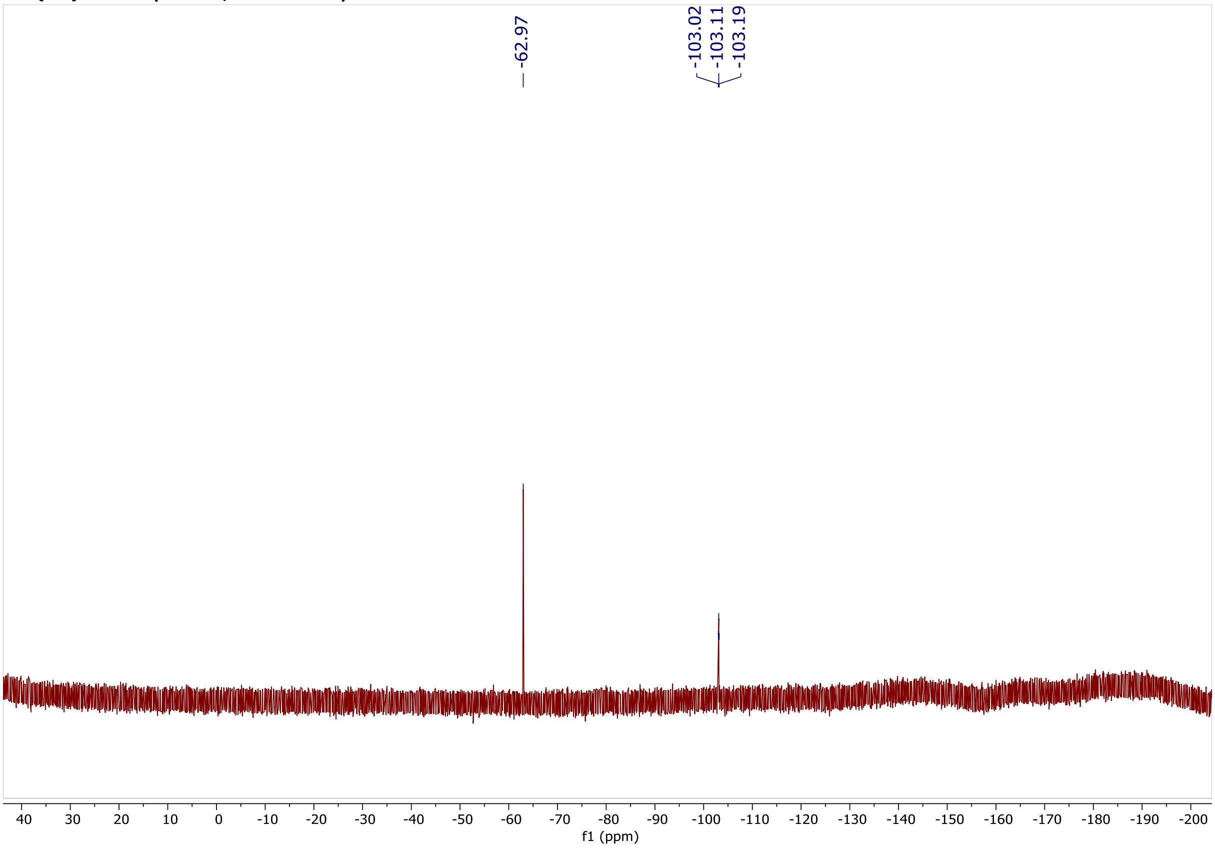

4,4'-sulfinylbis(nitrobenzene) 4p:

^{1}H NMR ($CDCl_3$, 300 MHz)

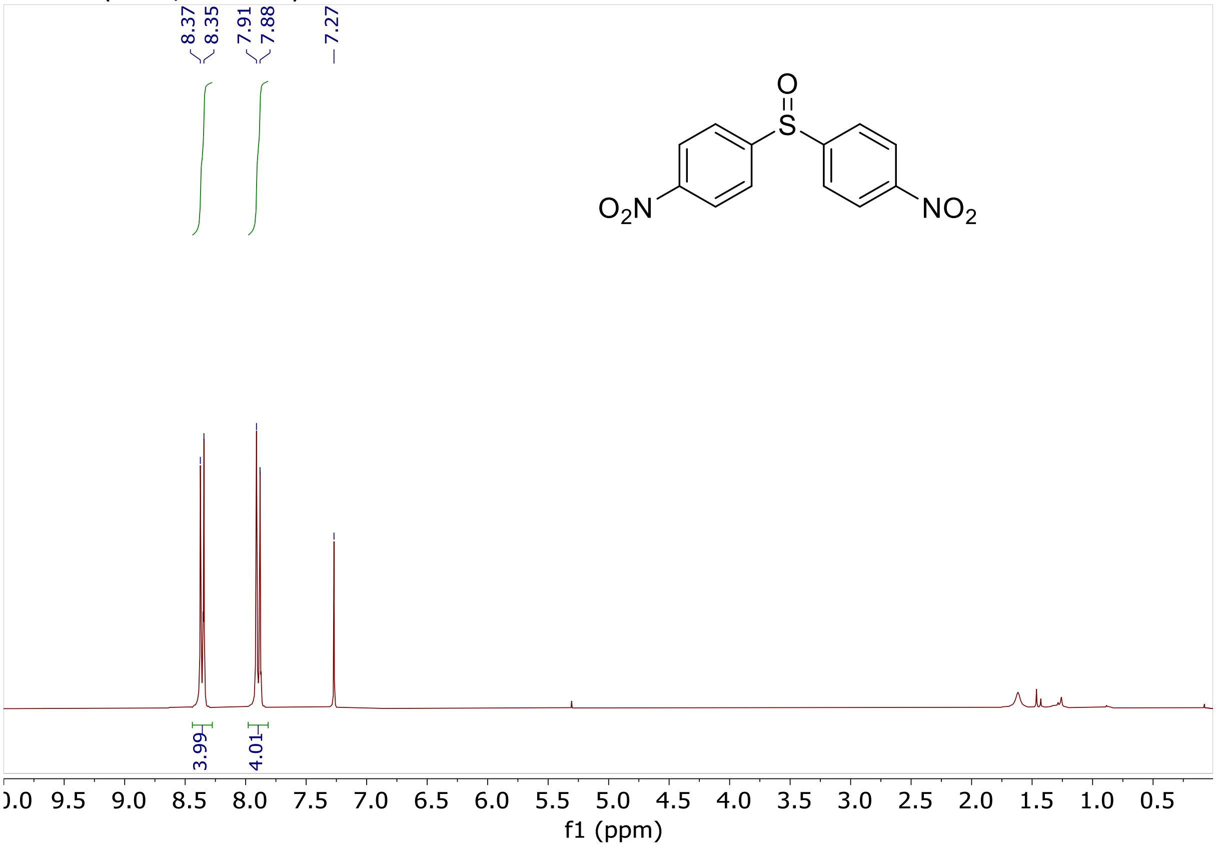

$^{13}C\{^{1}H\}$ NMR ($CDCl_3$, 75 MHz)

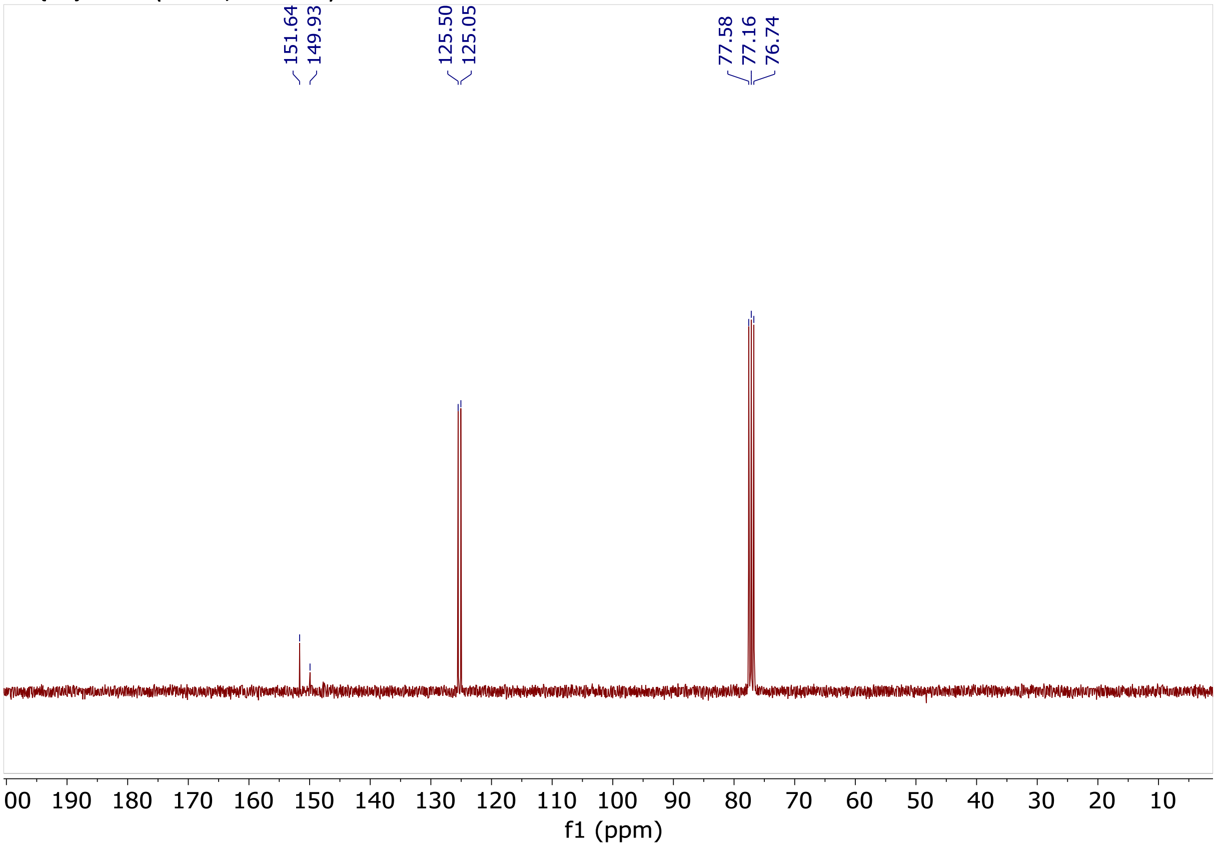

2,2'-sulfinylbis(methylbenzene) 4q:

^{1}H NMR ($CDCl_3$, 300 MHz)

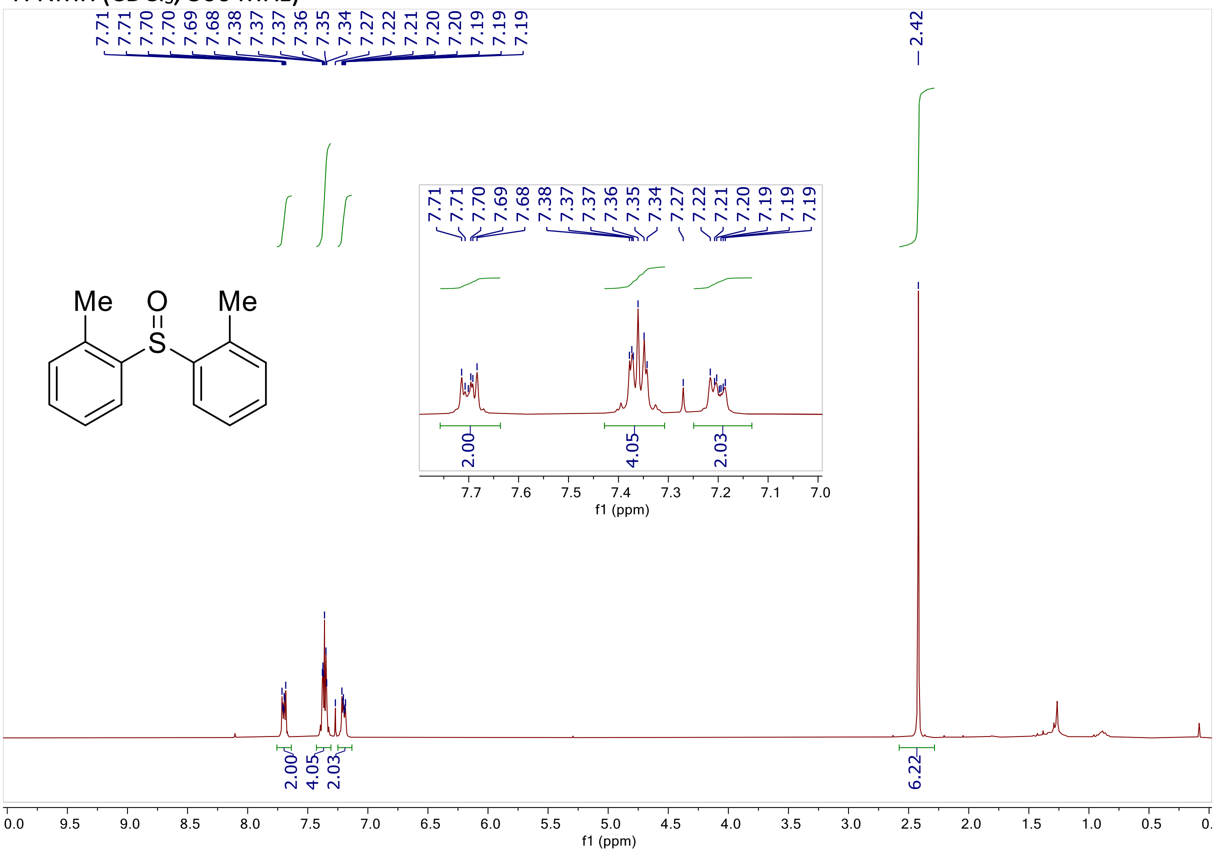

$^{13}C\{^{1}H\}$ NMR ($CDCl_3$, 75 MHz)

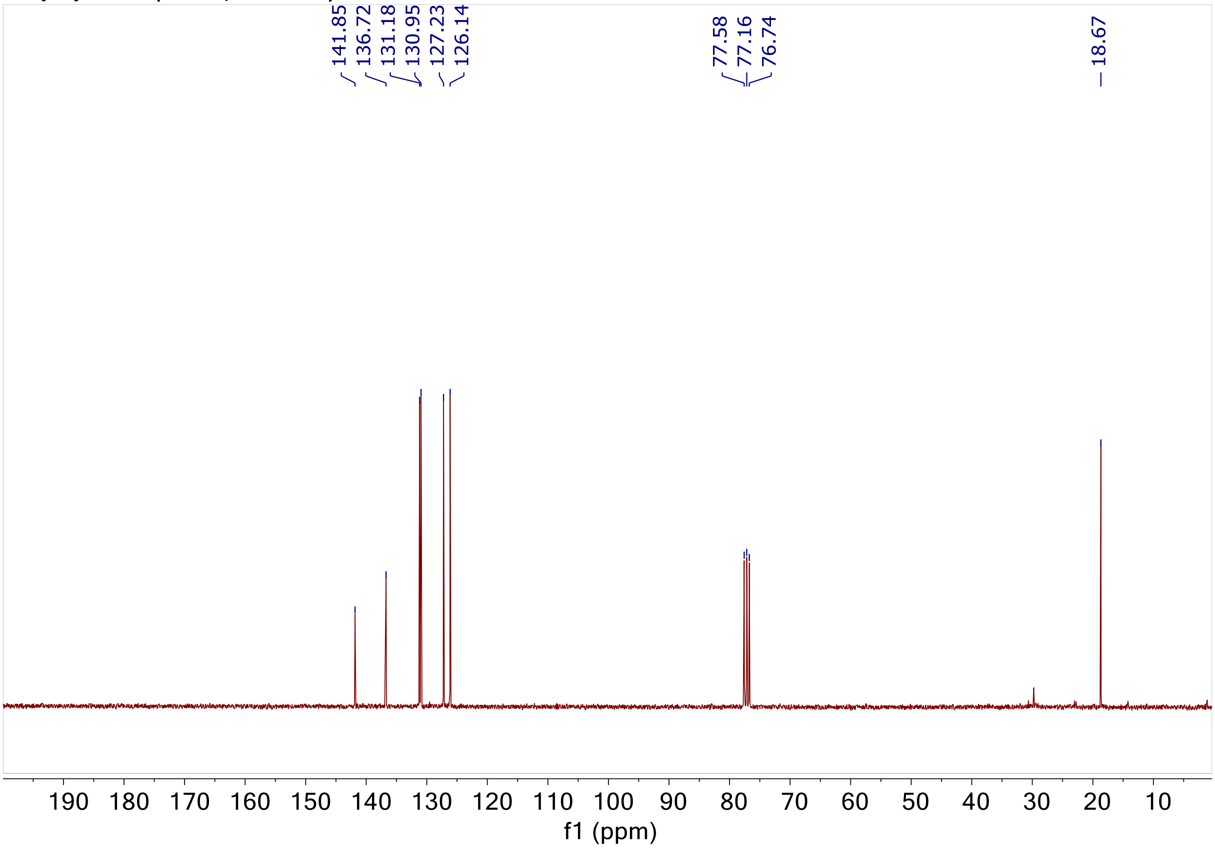

2,2'-sulfinylbis(methoxybenzene) 4r:

^1H NMR ($CDCl_3$, 300 MHz)

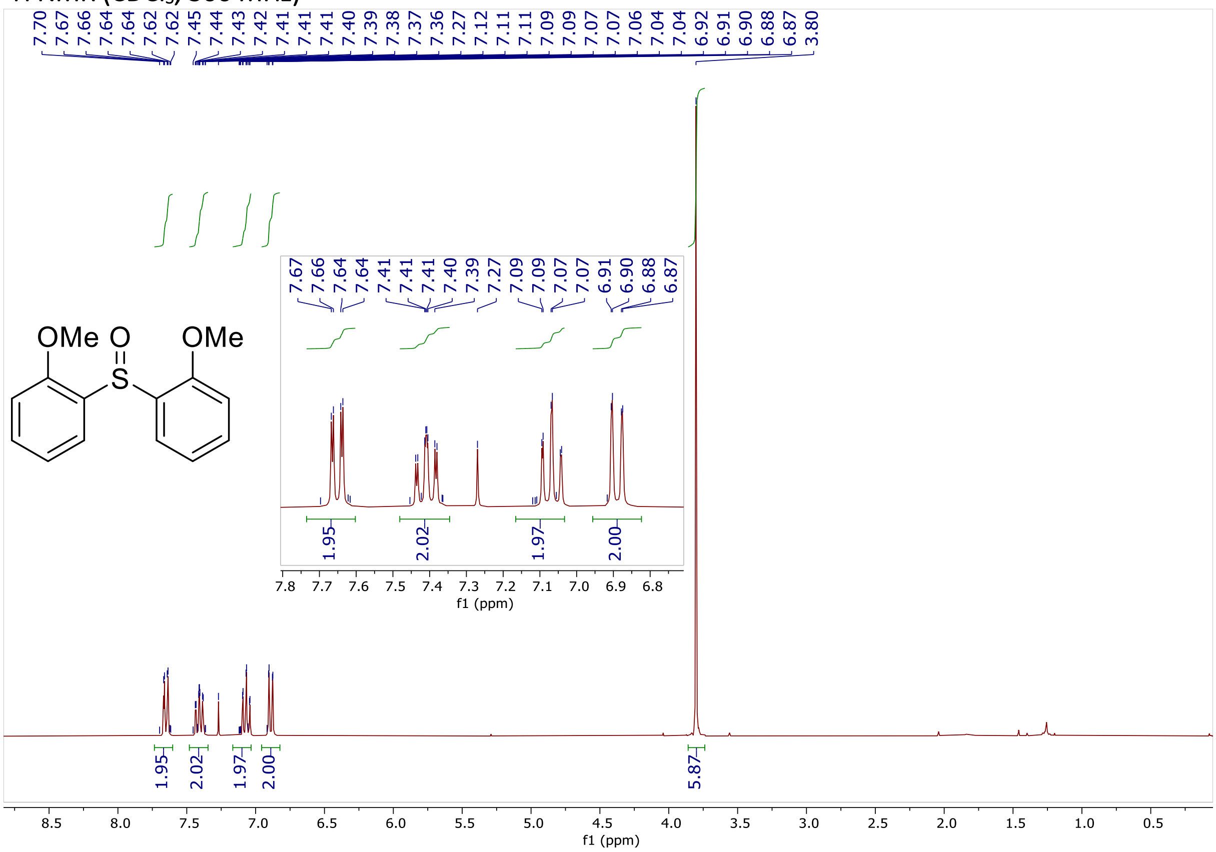

$^{13}C\{^1H\}$ NMR ($CDCl_3$, 75 MHz)

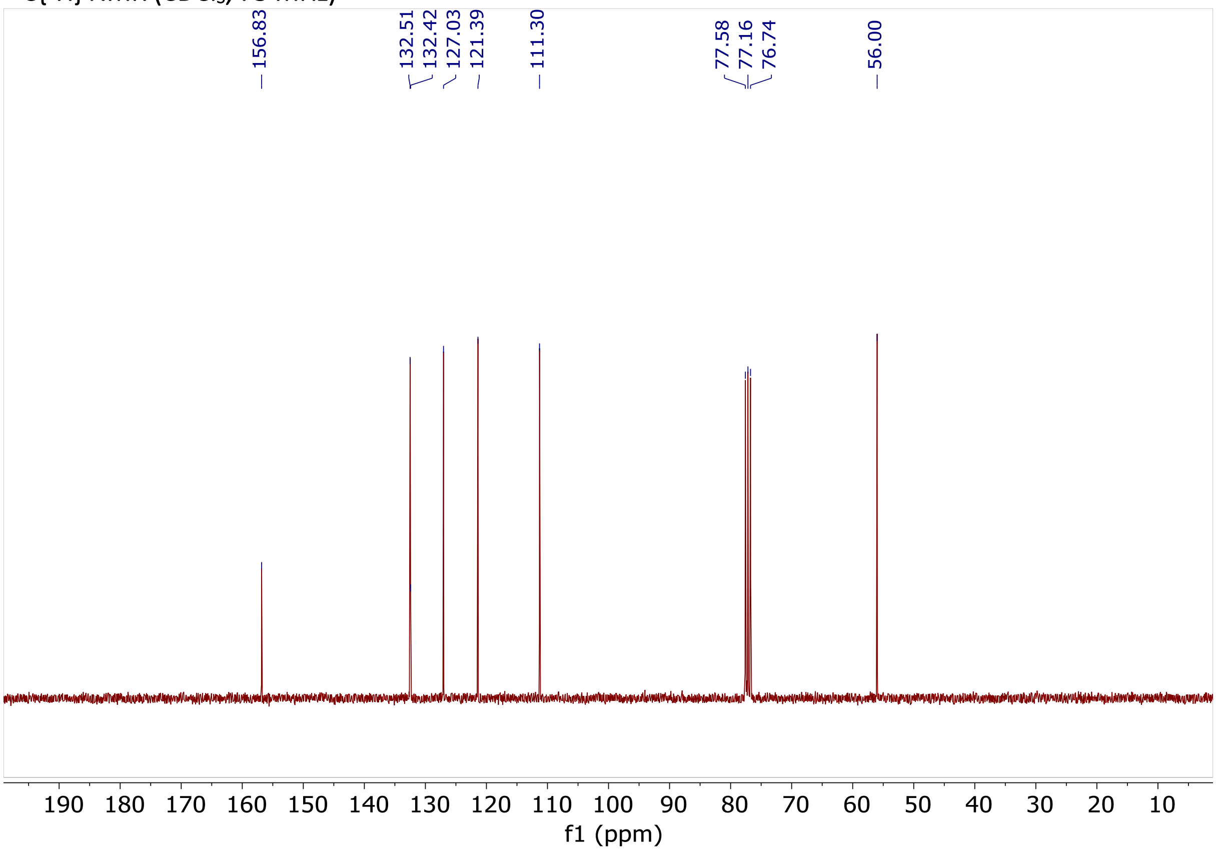

4,4'-sulfinyldidibenzo[b,d]thiophene 4s:

^{1}H NMR ($CDCl_3$, 300 MHz)

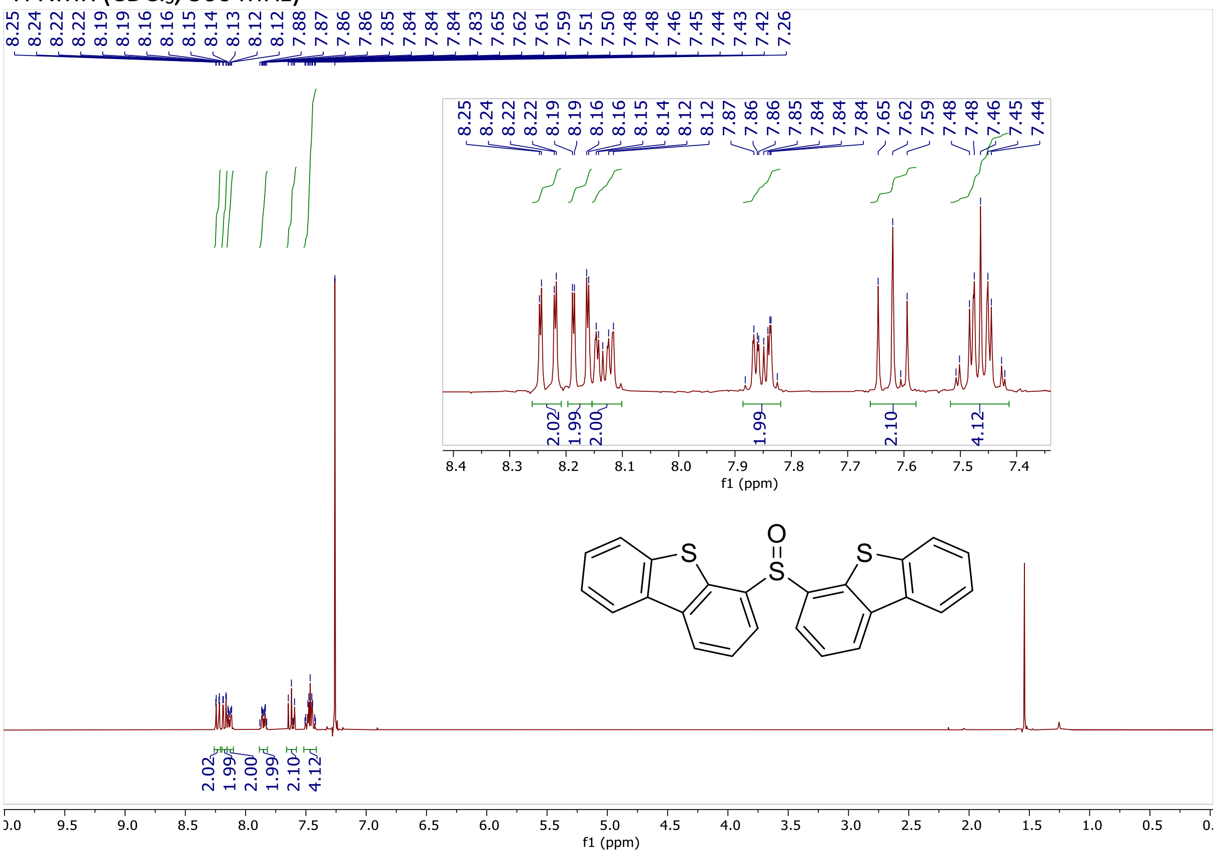

^{13}C{^{1}H} NMR ($CDCl_3$, 75 MHz)

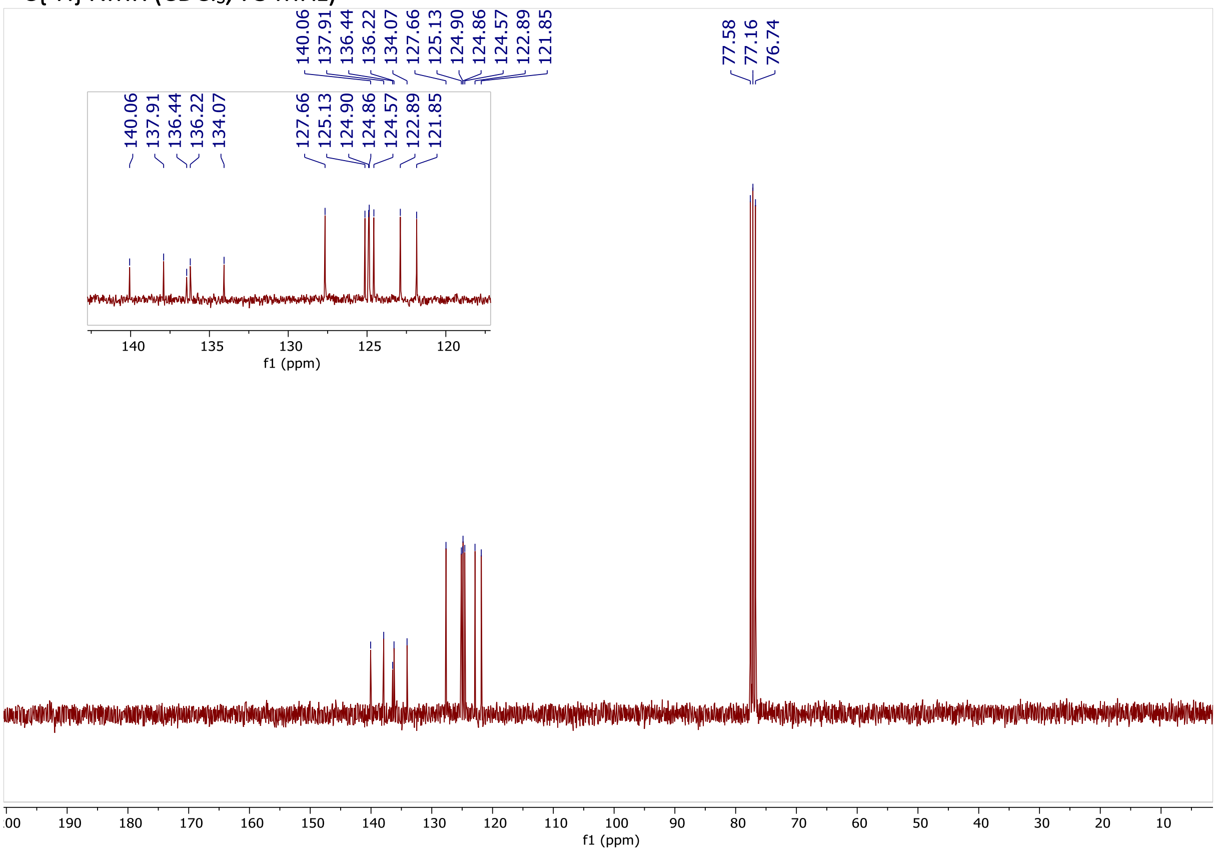

1,1'-sulfinyldinaphthalene 4t:

^{1}H NMR ($CDCl_3$, 300 MHz)

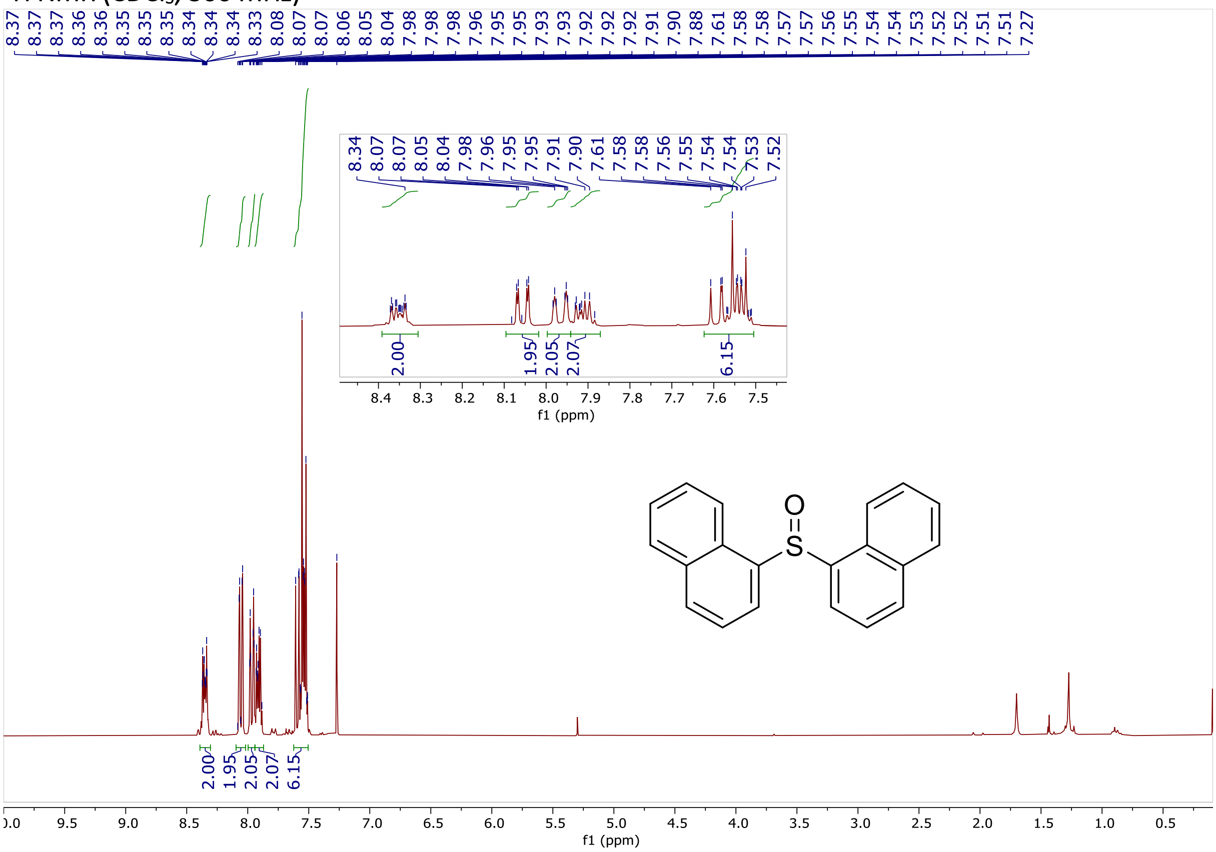

$^{13}C\{^{1}H\}$ NMR ($CDCl_3$, 75 MHz)

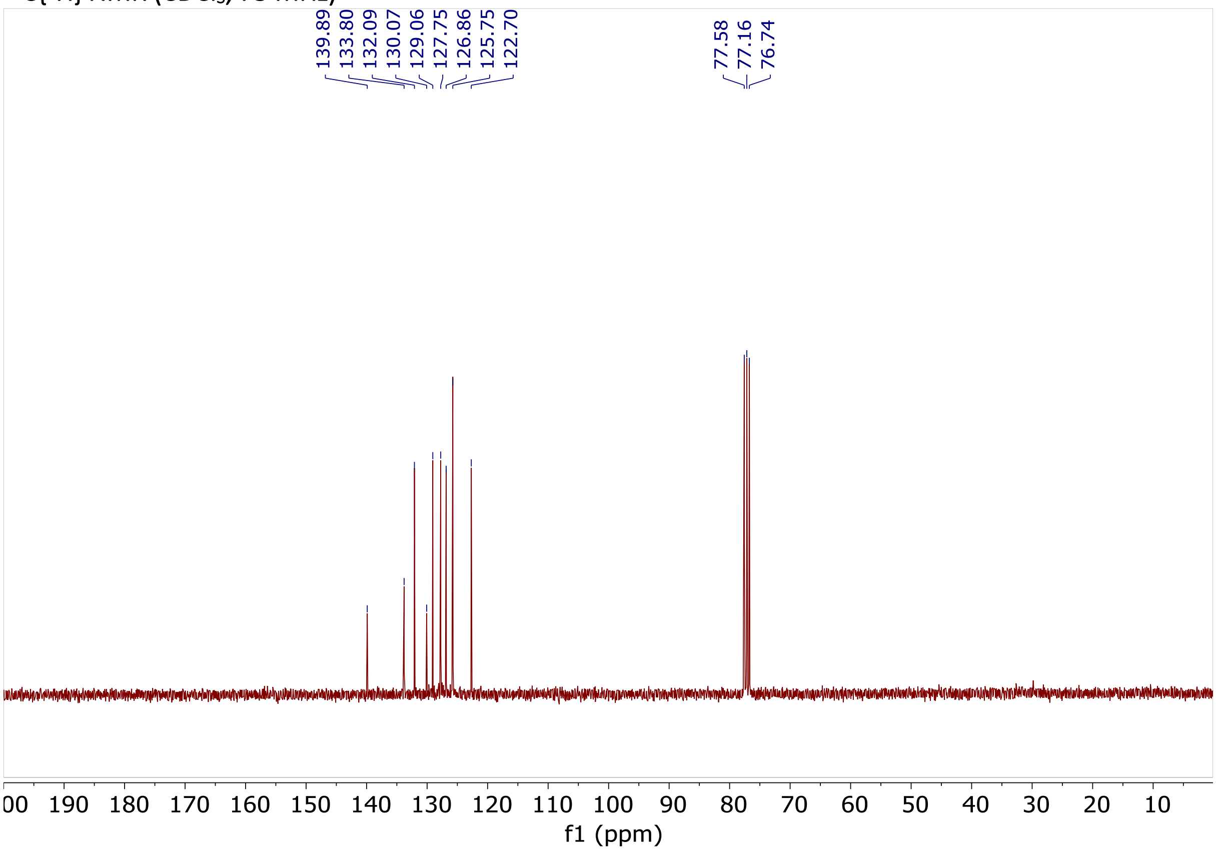

9,9'-sulfinyldianthracene 4u:

^{1}H NMR (CD_2Cl_2, 300 MHz)

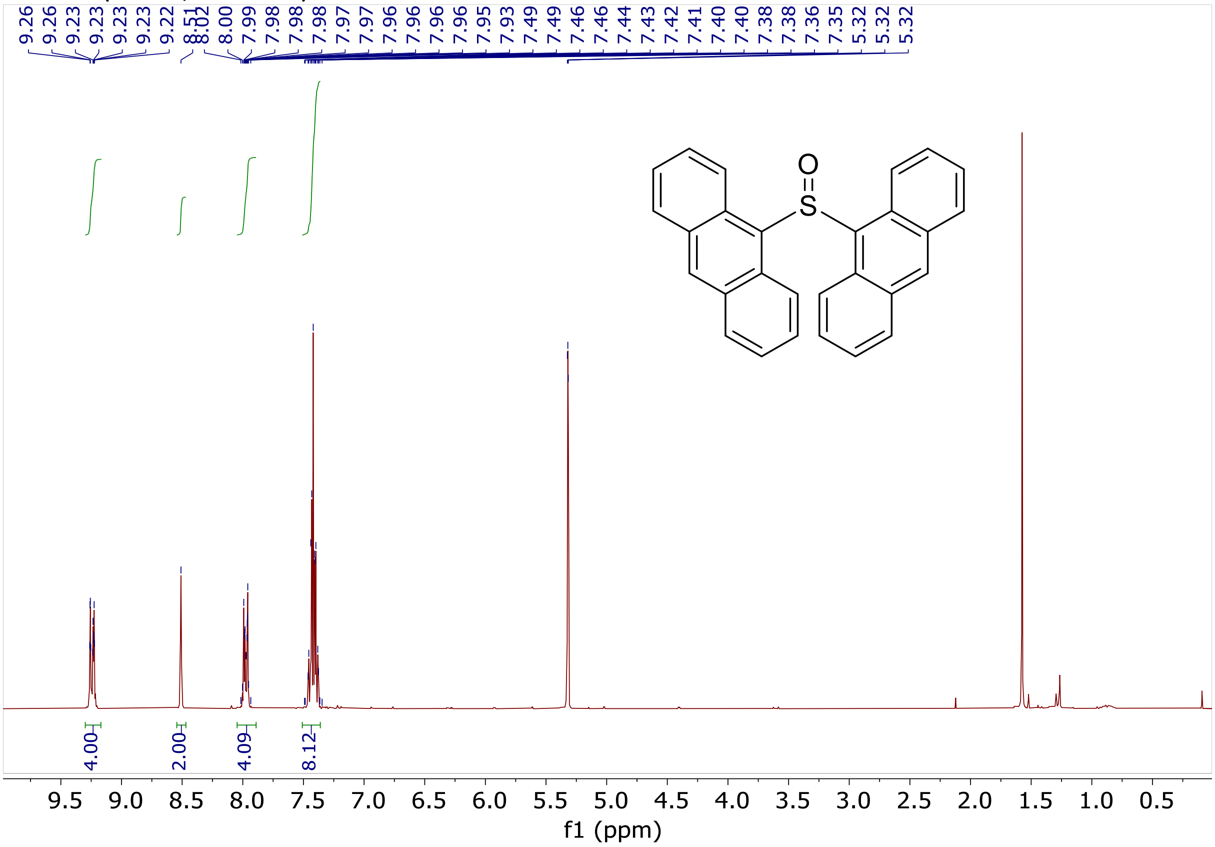

$^{13}C\{^1H\}$ NMR (CD_2Cl_2, 75 MHz)

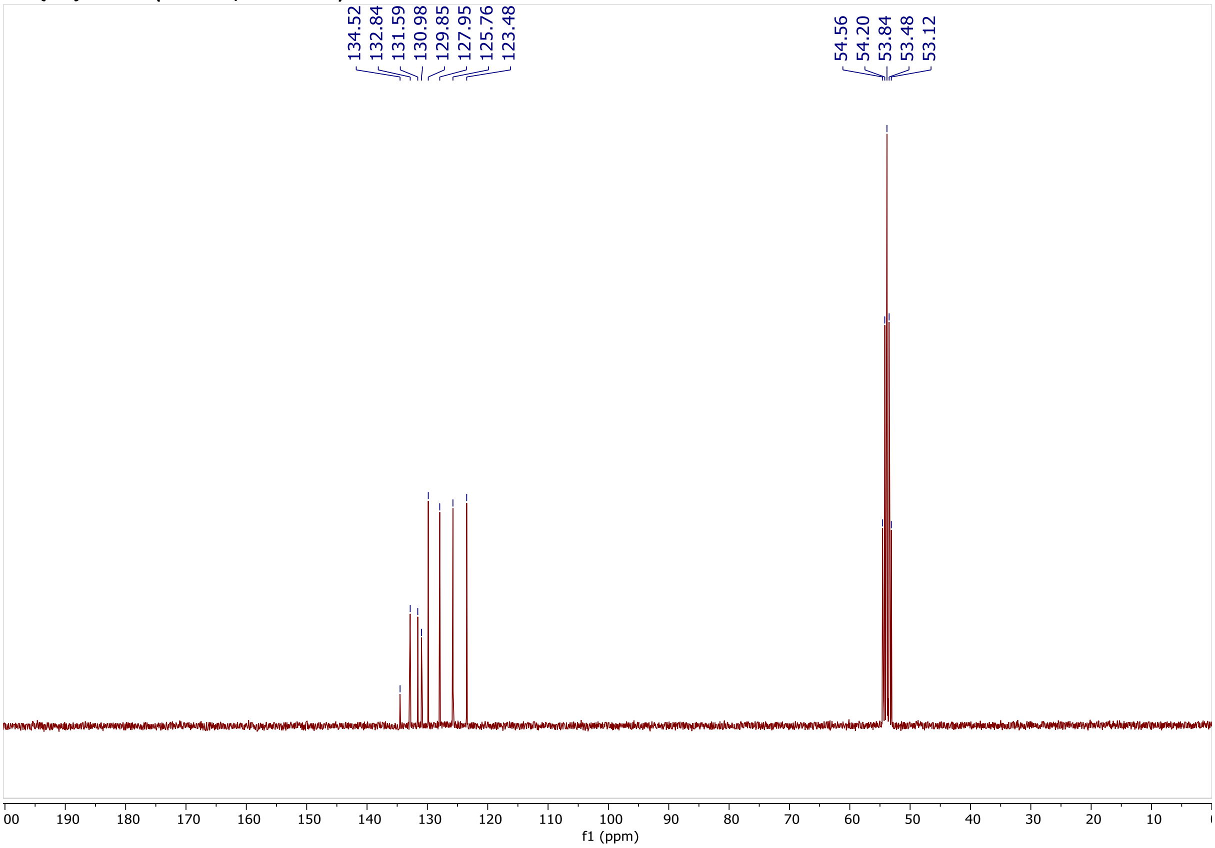

2,2'-sulfinylbis(1,3,5-trimethylbenzene) 4v:

^{1}H NMR ($CDCl_3$, 300 MHz)

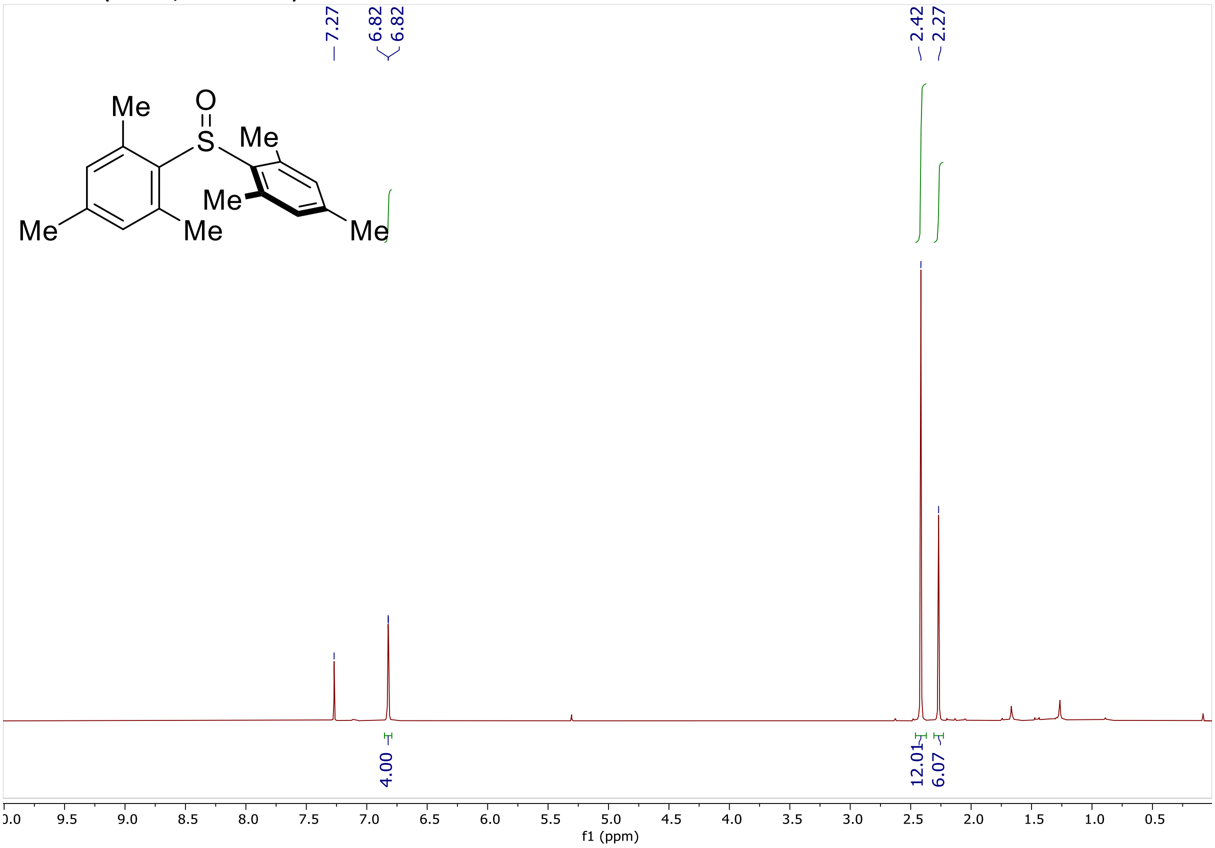

$^{13}C\{^{1}H\}$ NMR ($CDCl_3$, 75 MHz)

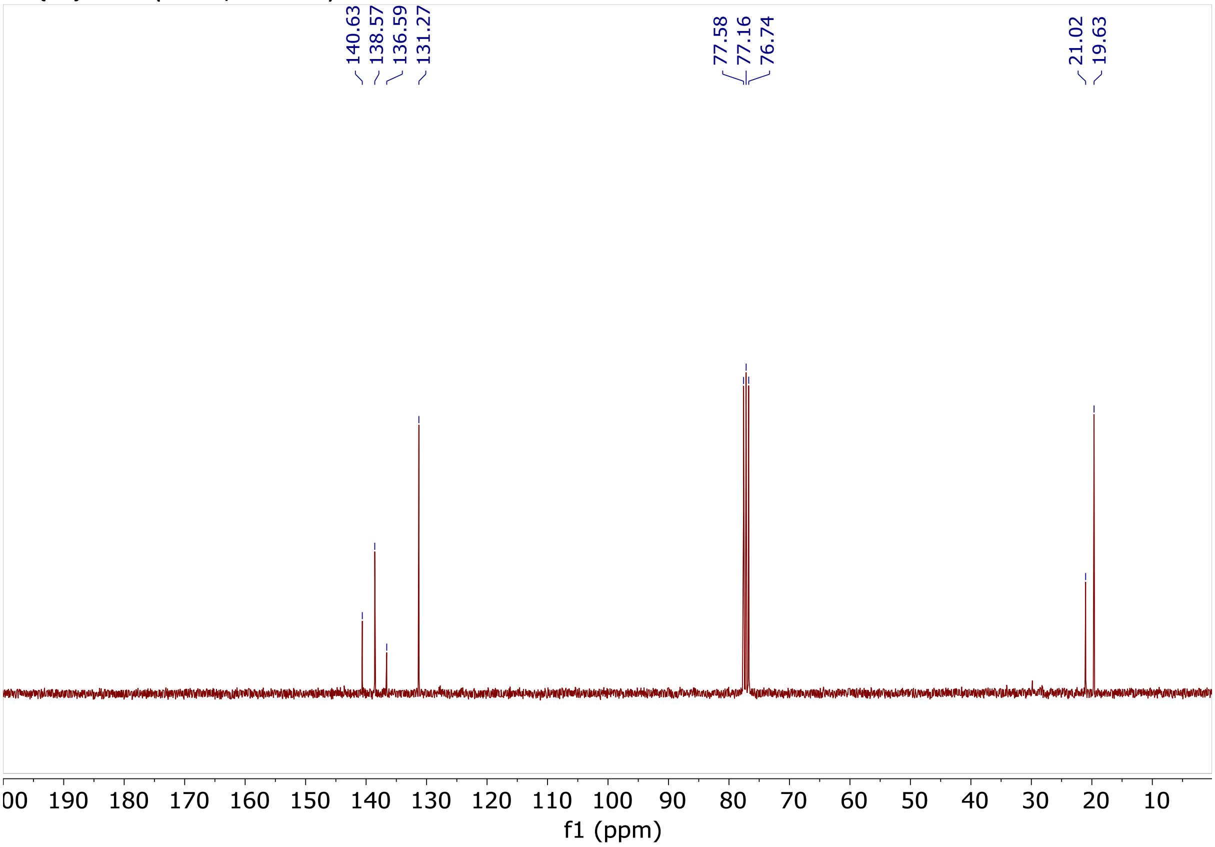

3,3'-sulfinyldithiophene:tert-butyl 3-(thiophen-3-ylsulfinyl)propanoate 4w: tert-butyl 3-(thiophen-3-ylsulfinyl)propanoate 4w' 90:10

^{1}H NMR ($CDCl_3$, 300 MHz)

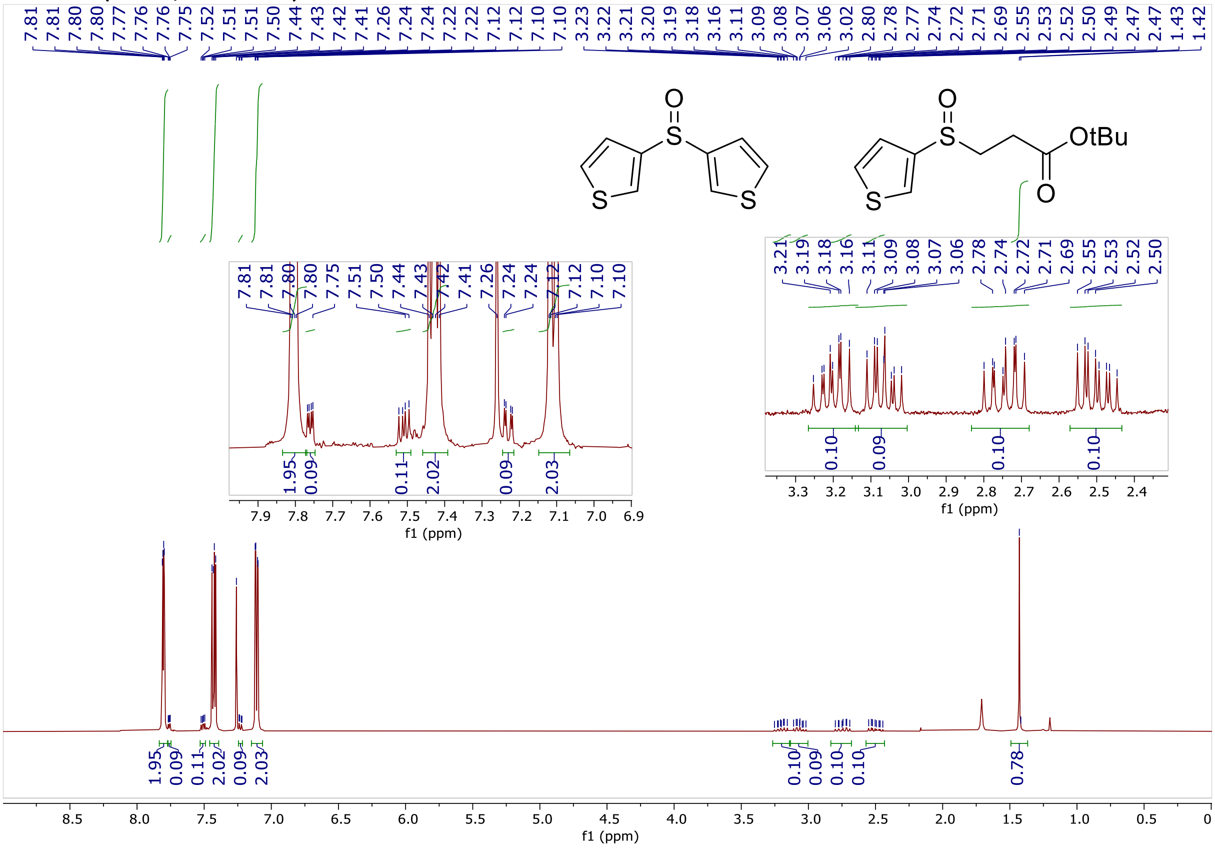

^{13}C{^{1}H} NMR ($CDCl_3$, 75 MHz)

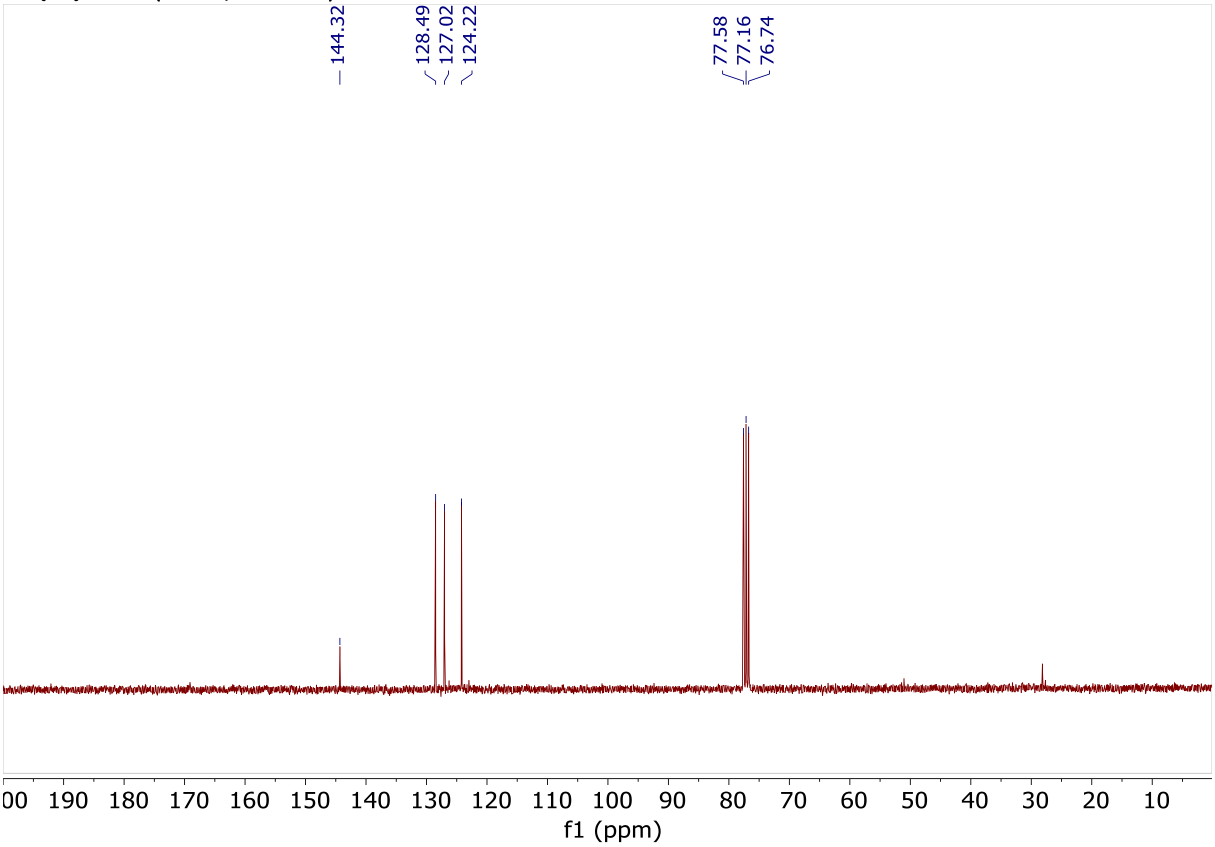

S3 XRay diffraction data :

Molecule **1** :

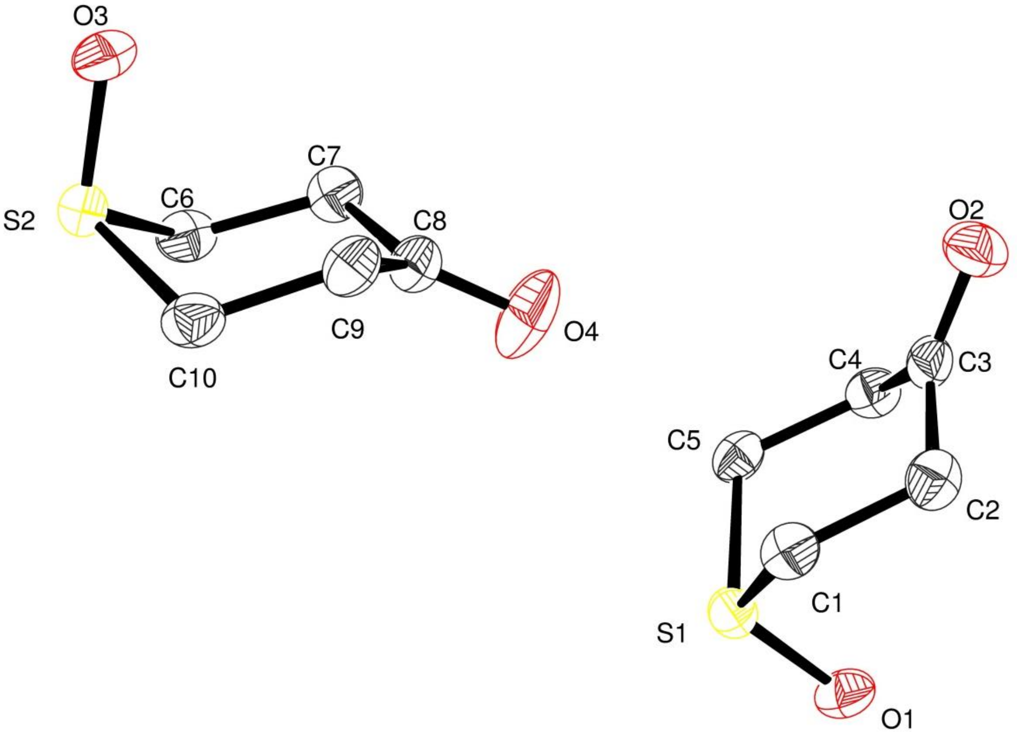

Figure 1 : Asymmetric Unit

Table 1. Crystal data and structure refinement for **1**.

Empirical formula	C5 H8 O2 S
Formula weight	132.17
Temperature	193(2) K
Wavelength	0.71073 A
Crystal system, space group	Monoclinic, P 21/c

Unit cell dimensions a = 18.2669(8) A alpha = 90 deg.
b = 6.3393(3) A beta = 107.215(3) deg.
c = 11.1426(5) A gamma = 90 deg.

Volume 1232.50(10) A^3

Z, Calculated density 8, 1.425 Mg/m^3

Absorption coefficient 0.428 mm^-1

F(000) 560

Crystal size 0.100 x 0.080 x 0.060 mm

Theta range for data collection 1.167 to 26.434 deg.

Limiting indices -22<=h<=22, -7<=k<=7, -13<=l<=13

Reflections collected / unique 11349 / 2480 [R(int) = 0.0391]

Completeness to theta = 25.242 99.0 %

Refinement method Full-matrix least-squares on F^2

Data / restraints / parameters 2480 / 0 / 145

Goodness-of-fit on F^2 1.130

Final R indices [I>2sigma(I)] R1 = 0.0402, wR2 = 0.1007

R indices (all data) R1 = 0.0502, wR2 = 0.1057

Largest diff. peak and hole 0.625 and -0.286 e.A^-3

Table 2. Atomic coordinates (x 10^4) and equivalent isotropic displacement parameters (A^2 x 10^3) for **1**.
U(eq) is defined as one third of the trace of the orthogonalized Uij tensor.

	x	y	z	U(eq)
C(1)	1267(1)	6993(4)	8187(2)	30(1)
C(2)	1313(1)	4987(4)	8967(2)	31(1)
C(3)	1666(1)	3156(4)	8487(2)	28(1)
C(4)	1325(1)	2597(4)	7130(2)	29(1)
C(5)	1263(1)	4472(4)	6244(2)	28(1)
C(6)	3748(1)	9669(4)	5014(2)	32(1)
C(7)	3655(1)	7822(4)	5831(2)	30(1)
C(8)	3320(1)	8401(4)	6860(2)	30(1)
C(9)	3667(1)	10244(4)	7672(2)	30(1)
C(10)	3732(1)	12223(4)	6928(2)	32(1)
O(1)	-43(1)	5663(3)	6616(2)	34(1)
O(2)	2191(1)	2153(3)	9175(2)	41(1)
O(3)	5048(1)	10815(3)	6682(2)	34(1)
O(4)	2807(1)	7392(4)	7059(2)	58(1)
S(1)	703(1)	6603(1)	6577(1)	29(1)
S(2)	4313(1)	11784(1)	5900(1)	31(1)

Table 3. Bond lengths [A] and angles [deg] for **1**.

C(1)-C(2)	1.529(3)
C(1)-S(1)	1.802(2)
C(1)-H(1A)	0.9900
C(1)-H(1B)	0.9900
C(2)-C(3)	1.500(3)
C(2)-H(2A)	0.9900
C(2)-H(2B)	0.9900
C(3)-O(2)	1.215(3)
C(3)-C(4)	1.498(3)
C(4)-C(5)	1.528(3)
C(4)-H(4A)	0.9900
C(4)-H(4B)	0.9900
C(5)-S(1)	1.799(2)
C(5)-H(5A)	0.9900
C(5)-H(5B)	0.9900
C(6)-C(7)	1.524(3)
C(6)-S(2)	1.798(2)
C(6)-H(6A)	0.9900
C(6)-H(6B)	0.9900
C(7)-C(8)	1.497(3)
C(7)-H(7A)	0.9900
C(7)-H(7B)	0.9900
C(8)-O(4)	1.207(3)
C(8)-C(9)	1.499(3)
C(9)-C(10)	1.528(3)
C(9)-H(9A)	0.9900
C(9)-H(9B)	0.9900
C(10)-S(2)	1.799(3)
C(10)-H(10A)	0.9900
C(10)-H(10B)	0.9900
O(1)-S(1)	1.5000(17)
O(3)-S(2)	1.5002(16)
C(2)-C(1)-S(1)	111.80(15)
C(2)-C(1)-H(1A)	109.3
S(1)-C(1)-H(1A)	109.3
C(2)-C(1)-H(1B)	109.3
S(1)-C(1)-H(1B)	109.3
H(1A)-C(1)-H(1B)	107.9
C(3)-C(2)-C(1)	113.48(19)
C(3)-C(2)-H(2A)	108.9
C(1)-C(2)-H(2A)	108.9

C(3)-C(2)-H(2B)	108.9
C(1)-C(2)-H(2B)	108.9
H(2A)-C(2)-H(2B)	107.7
O(2)-C(3)-C(4)	121.8(2)
O(2)-C(3)-C(2)	121.5(2)
C(4)-C(3)-C(2)	116.69(19)
C(3)-C(4)-C(5)	113.41(19)
C(3)-C(4)-H(4A)	108.9
C(5)-C(4)-H(4A)	108.9
C(3)-C(4)-H(4B)	108.9
C(5)-C(4)-H(4B)	108.9
H(4A)-C(4)-H(4B)	107.7
C(4)-C(5)-S(1)	112.87(16)
C(4)-C(5)-H(5A)	109.0
S(1)-C(5)-H(5A)	109.0
C(4)-C(5)-H(5B)	109.0
S(1)-C(5)-H(5B)	109.0
H(5A)-C(5)-H(5B)	107.8
C(7)-C(6)-S(2)	113.10(15)
C(7)-C(6)-H(6A)	109.0
S(2)-C(6)-H(6A)	109.0
C(7)-C(6)-H(6B)	109.0
S(2)-C(6)-H(6B)	109.0
H(6A)-C(6)-H(6B)	107.8
C(8)-C(7)-C(6)	114.3(2)
C(8)-C(7)-H(7A)	108.7
C(6)-C(7)-H(7A)	108.7
C(8)-C(7)-H(7B)	108.7
C(6)-C(7)-H(7B)	108.7
H(7A)-C(7)-H(7B)	107.6
O(4)-C(8)-C(7)	121.8(2)
O(4)-C(8)-C(9)	120.9(2)
C(7)-C(8)-C(9)	117.2(2)
C(8)-C(9)-C(10)	113.53(19)
C(8)-C(9)-H(9A)	108.9
C(10)-C(9)-H(9A)	108.9
C(8)-C(9)-H(9B)	108.9
C(10)-C(9)-H(9B)	108.9
H(9A)-C(9)-H(9B)	107.7
C(9)-C(10)-S(2)	111.98(16)
C(9)-C(10)-H(10A)	109.2
S(2)-C(10)-H(10A)	109.2
C(9)-C(10)-H(10B)	109.2
S(2)-C(10)-H(10B)	109.2
H(10A)-C(10)-H(10B)	107.9

O(1)-S(1)-C(5)	106.57(10)
O(1)-S(1)-C(1)	106.39(10)
C(5)-S(1)-C(1)	96.65(11)
O(3)-S(2)-C(6)	106.49(11)
O(3)-S(2)-C(10)	106.77(11)
C(6)-S(2)-C(10)	96.59(11)

Symmetry transformations used to generate equivalent atoms:

Table 4. Anisotropic displacement parameters (A^2 x 10^3) for **1**.
The anisotropic displacement factor exponent takes the form:
-2 pi^2 [h^2 a*^2 U11 + ... + 2 h k a* b* U12]

	U11	U22	U33	U23	U13	U12
C(1)	28(1)	27(1)	33(1)	-7(1)	9(1)	-4(1)
C(2)	32(1)	36(1)	23(1)	-3(1)	8(1)	-3(1)
C(3)	23(1)	29(1)	31(1)	4(1)	8(1)	-5(1)
C(4)	28(1)	27(1)	32(1)	-5(1)	10(1)	0(1)
C(5)	25(1)	36(1)	25(1)	-4(1)	9(1)	-3(1)
C(6)	29(1)	42(1)	24(1)	0(1)	7(1)	4(1)
C(7)	31(1)	26(1)	32(1)	-5(1)	9(1)	-3(1)
C(8)	27(1)	30(1)	34(1)	1(1)	9(1)	-4(1)
C(9)	29(1)	35(1)	29(1)	-5(1)	12(1)	-3(1)
C(10)	28(1)	28(1)	39(1)	-5(1)	11(1)	2(1)
O(1)	21(1)	40(1)	40(1)	-1(1)	7(1)	1(1)
O(2)	32(1)	42(1)	45(1)	11(1)	2(1)	4(1)
O(3)	20(1)	40(1)	42(1)	1(1)	7(1)	1(1)
O(4)	51(1)	60(1)	74(2)	-14(1)	36(1)	-29(1)
S(1)	27(1)	28(1)	31(1)	5(1)	7(1)	0(1)
S(2)	26(1)	30(1)	37(1)	8(1)	11(1)	1(1)

Molecule **2**:

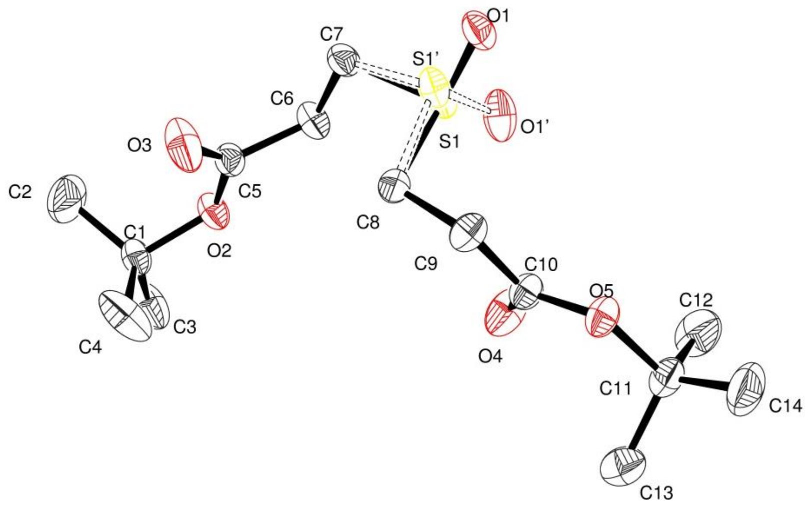

Figure 1 : Asymmetric Unit

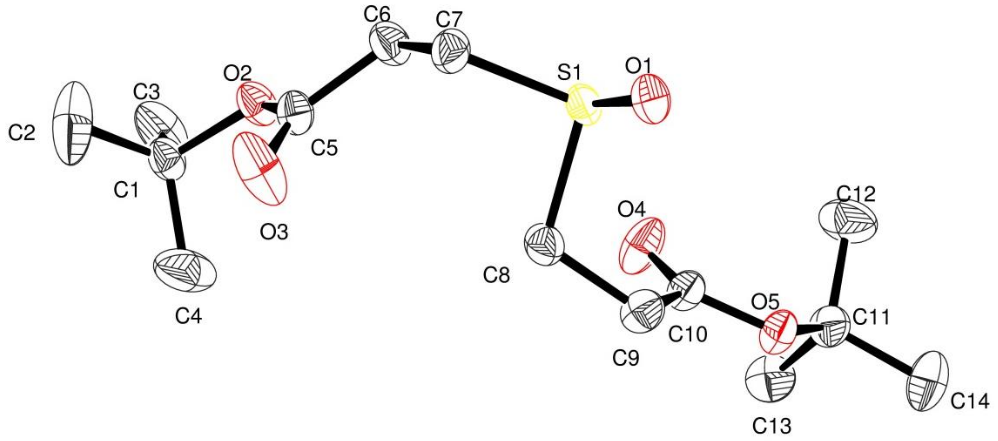

Figure 2 : Compound

Table 1. Crystal data and structure refinement for **2**.

Empirical formula	C14 H26 O5 S
Formula weight	306.41
Temperature	193(2) K
Wavelength	0.71073 A
Crystal system, space group	Monoclinic, C 2/c

Unit cell dimensions a = 22.8590(15) A alpha = 90 deg.
b = 5.7148(4) A beta = 94.984(2) deg.
c = 25.7983(15) A gamma = 90 deg.

Volume 3357.4(4) A^3

Z, Calculated density 8, 1.212 Mg/m^3

Absorption coefficient 0.208 mm^-1

F(000) 1328

Crystal size 0.400 x 0.180 x 0.080 mm

Theta range for data collection 3.503 to 35.087 deg.

Limiting indices -36<=h<=36, -9<=k<=9, -41<=l<=41

Reflections collected / unique 130538 / 7361 [R(int) = 0.0477]

Completeness to theta = 25.242 98.3 %

Refinement method Full-matrix least-squares on F^2

Data / restraints / parameters 7361 / 37 / 207

Goodness-of-fit on F^2 1.107

Final R indices [I>2sigma(I)] R1 = 0.0452, wR2 = 0.1205

R indices (all data) R1 = 0.0527, wR2 = 0.1255

Largest diff. peak and hole 0.417 and -0.322 e.A^-3

Table 2. Atomic coordinates (x 10^4) and equivalent isotropic displacement parameters (A^2 x 10^3) for **2**.
U(eq) is defined as one third of the trace of the orthogonalized Uij tensor.

	x	y	z	U(eq)
C(1)	8556(1)	2886(2)	7530(1)	31(1)
C(2)	9199(1)	2595(4)	7464(1)	73(1)
C(3)	8468(1)	4214(2)	8027(1)	53(1)
C(4)	8220(1)	618(2)	7533(1)	61(1)
C(5)	8252(1)	3849(2)	6617(1)	28(1)
C(6)	7969(1)	5800(2)	6290(1)	33(1)
C(7)	7875(1)	5221(2)	5712(1)	31(1)
C(8)	6934(1)	2309(2)	5772(1)	30(1)
S(1)	7094(1)	5191(1)	5511(1)	26(1)
O(1)	7045(1)	4901(2)	4931(1)	34(1)
S(1')	7153(1)	4695(4)	5399(1)	31(1)
O(1')	6801(2)	6737(5)	5554(1)	47(1)
C(9)	6297(1)	1670(2)	5612(1)	31(1)
C(10)	5863(1)	3050(2)	5898(1)	28(1)
C(11)	4807(1)	3859(2)	5887(1)	34(1)
C(12)	4866(1)	6505(2)	5893(1)	55(1)
C(13)	4737(1)	2870(2)	6424(1)	44(1)
C(14)	4302(1)	3089(3)	5506(1)	54(1)
O(2)	8288(1)	4451(1)	7118(1)	32(1)
O(3)	8421(1)	2033(2)	6448(1)	56(1)
O(4)	5992(1)	4199(2)	6282(1)	45(1)
O(5)	5321(1)	2774(1)	5668(1)	31(1)

Table 3. Bond lengths [A] and angles [deg] for **2**.

C(1)-O(2)	1.4815(11)
C(1)-C(2)	1.5024(18)
C(1)-C(4)	1.5066(17)
C(1)-C(3)	1.5183(15)
C(2)-H(2A)	0.9800
C(2)-H(2B)	0.9800
C(2)-H(2C)	0.9800
C(3)-H(3A)	0.9800
C(3)-H(3B)	0.9800
C(3)-H(3C)	0.9800
C(4)-H(4A)	0.9800
C(4)-H(4B)	0.9800
C(4)-H(4C)	0.9800
C(5)-O(3)	1.2020(12)
C(5)-O(2)	1.3319(11)
C(5)-C(6)	1.5095(13)
C(6)-C(7)	1.5256(13)
C(6)-H(6A)	0.9900
C(6)-H(6B)	0.9900
C(7)-S(1')	1.798(2)
C(7)-S(1)	1.8142(11)
C(7)-H(7A)	0.9900
C(7)-H(7B)	0.9900
C(8)-C(9)	1.5215(13)
C(8)-S(1')	1.767(2)
C(8)-S(1)	1.8286(12)
C(8)-H(8A)	0.9900
C(8)-H(8B)	0.9900
S(1)-O(1)	1.5014(12)
S(1')-O(1')	1.491(4)
C(9)-C(10)	1.5104(13)
C(9)-H(9A)	0.9900
C(9)-H(9B)	0.9900
C(10)-O(4)	1.2039(11)
C(10)-O(5)	1.3345(11)
C(11)-O(5)	1.4823(12)
C(11)-C(13)	1.5162(16)
C(11)-C(14)	1.5167(16)
C(11)-C(12)	1.5180(17)
C(12)-H(12A)	0.9800
C(12)-H(12B)	0.9800
C(12)-H(12C)	0.9800

C(13)-H(13A)	0.9800
C(13)-H(13B)	0.9800
C(13)-H(13C)	0.9800
C(14)-H(14A)	0.9800
C(14)-H(14B)	0.9800
C(14)-H(14C)	0.9800
O(2)-C(1)-C(2)	109.47(10)
O(2)-C(1)-C(4)	110.06(9)
C(2)-C(1)-C(4)	114.10(14)
O(2)-C(1)-C(3)	103.20(8)
C(2)-C(1)-C(3)	110.68(12)
C(4)-C(1)-C(3)	108.79(11)
C(1)-C(2)-H(2A)	109.5
C(1)-C(2)-H(2B)	109.5
H(2A)-C(2)-H(2B)	109.5
C(1)-C(2)-H(2C)	109.5
H(2A)-C(2)-H(2C)	109.5
H(2B)-C(2)-H(2C)	109.5
C(1)-C(3)-H(3A)	109.5
C(1)-C(3)-H(3B)	109.5
H(3A)-C(3)-H(3B)	109.5
C(1)-C(3)-H(3C)	109.5
H(3A)-C(3)-H(3C)	109.5
H(3B)-C(3)-H(3C)	109.5
C(1)-C(4)-H(4A)	109.5
C(1)-C(4)-H(4B)	109.5
H(4A)-C(4)-H(4B)	109.5
C(1)-C(4)-H(4C)	109.5
H(4A)-C(4)-H(4C)	109.5
H(4B)-C(4)-H(4C)	109.5
O(3)-C(5)-O(2)	125.43(8)
O(3)-C(5)-C(6)	124.76(8)
O(2)-C(5)-C(6)	109.80(8)
C(5)-C(6)-C(7)	113.82(8)
C(5)-C(6)-H(6A)	108.8
C(7)-C(6)-H(6A)	108.8
C(5)-C(6)-H(6B)	108.8
C(7)-C(6)-H(6B)	108.8
H(6A)-C(6)-H(6B)	107.7
C(6)-C(7)-S(1')	121.37(11)
C(6)-C(7)-S(1)	109.40(7)
C(6)-C(7)-H(7A)	109.8
S(1)-C(7)-H(7A)	109.8
C(6)-C(7)-H(7B)	109.8

S(1)-C(7)-H(7B)	109.8
H(7A)-C(7)-H(7B)	108.2
C(9)-C(8)-S(1')	110.28(9)
C(9)-C(8)-S(1)	109.50(7)
C(9)-C(8)-H(8A)	109.8
S(1)-C(8)-H(8A)	109.8
C(9)-C(8)-H(8B)	109.8
S(1)-C(8)-H(8B)	109.8
H(8A)-C(8)-H(8B)	108.2
O(1)-S(1)-C(7)	105.81(6)
O(1)-S(1)-C(8)	105.55(7)
C(7)-S(1)-C(8)	97.34(5)
O(1')-S(1')-C(8)	105.8(2)
O(1')-S(1')-C(7)	104.09(18)
C(8)-S(1')-C(7)	100.21(10)
C(10)-C(9)-C(8)	113.31(8)
C(10)-C(9)-H(9A)	108.9
C(8)-C(9)-H(9A)	108.9
C(10)-C(9)-H(9B)	108.9
C(8)-C(9)-H(9B)	108.9
H(9A)-C(9)-H(9B)	107.7
O(4)-C(10)-O(5)	125.54(9)
O(4)-C(10)-C(9)	124.23(8)
O(5)-C(10)-C(9)	110.22(8)
O(5)-C(11)-C(13)	110.01(8)
O(5)-C(11)-C(14)	102.37(9)
C(13)-C(11)-C(14)	110.47(10)
O(5)-C(11)-C(12)	110.41(9)
C(13)-C(11)-C(12)	112.31(11)
C(14)-C(11)-C(12)	110.84(11)
C(11)-C(12)-H(12A)	109.5
C(11)-C(12)-H(12B)	109.5
H(12A)-C(12)-H(12B)	109.5
C(11)-C(12)-H(12C)	109.5
H(12A)-C(12)-H(12C)	109.5
H(12B)-C(12)-H(12C)	109.5
C(11)-C(13)-H(13A)	109.5
C(11)-C(13)-H(13B)	109.5
H(13A)-C(13)-H(13B)	109.5
C(11)-C(13)-H(13C)	109.5
H(13A)-C(13)-H(13C)	109.5
H(13B)-C(13)-H(13C)	109.5
C(11)-C(14)-H(14A)	109.5
C(11)-C(14)-H(14B)	109.5
H(14A)-C(14)-H(14B)	109.5

C(11)-C(14)-H(14C)	109.5
H(14A)-C(14)-H(14C)	109.5
H(14B)-C(14)-H(14C)	109.5
C(5)-O(2)-C(1)	121.84(7)
C(10)-O(5)-C(11)	121.06(7)

Symmetry transformations used to generate equivalent atoms:

Table 4. Anisotropic displacement parameters (A^2 x 10^3) for **2**.
The anisotropic displacement factor exponent takes the form:
-2 pi^2 [h^2 a*^2 U11 + ... + 2 h k a* b* U12]

	U11	U22	U33	U23	U13	U12
C(1)	37(1)	30(1)	24(1)	0(1)	-5(1)	6(1)
C(2)	40(1)	128(2)	49(1)	14(1)	-6(1)	24(1)
C(3)	88(1)	45(1)	25(1)	-3(1)	-7(1)	18(1)
C(4)	99(1)	38(1)	42(1)	11(1)	-19(1)	-17(1)
C(5)	29(1)	32(1)	24(1)	1(1)	-1(1)	4(1)
C(6)	41(1)	29(1)	28(1)	3(1)	-4(1)	3(1)
C(7)	28(1)	39(1)	26(1)	7(1)	-3(1)	-4(1)
C(8)	24(1)	31(1)	36(1)	7(1)	4(1)	2(1)
S(1)	25(1)	27(1)	27(1)	6(1)	-1(1)	4(1)
O(1)	36(1)	39(1)	26(1)	7(1)	-7(1)	1(1)
S(1')	33(1)	28(1)	31(1)	4(1)	-7(1)	2(1)
O(1')	49(2)	31(1)	58(2)	0(1)	-9(1)	15(1)
C(9)	27(1)	31(1)	37(1)	-6(1)	5(1)	0(1)
C(10)	24(1)	30(1)	31(1)	-4(1)	1(1)	-1(1)
C(11)	24(1)	33(1)	45(1)	-2(1)	4(1)	3(1)
C(12)	46(1)	32(1)	88(1)	4(1)	18(1)	9(1)
C(13)	40(1)	47(1)	46(1)	-2(1)	13(1)	0(1)
C(14)	27(1)	73(1)	60(1)	-2(1)	-8(1)	1(1)
O(2)	44(1)	28(1)	23(1)	0(1)	-3(1)	7(1)
O(3)	86(1)	53(1)	28(1)	-6(1)	-5(1)	38(1)
O(4)	31(1)	61(1)	43(1)	-24(1)	-1(1)	-1(1)
O(5)	23(1)	35(1)	33(1)	-7(1)	-1(1)	1(1)

Molecule **4b**:

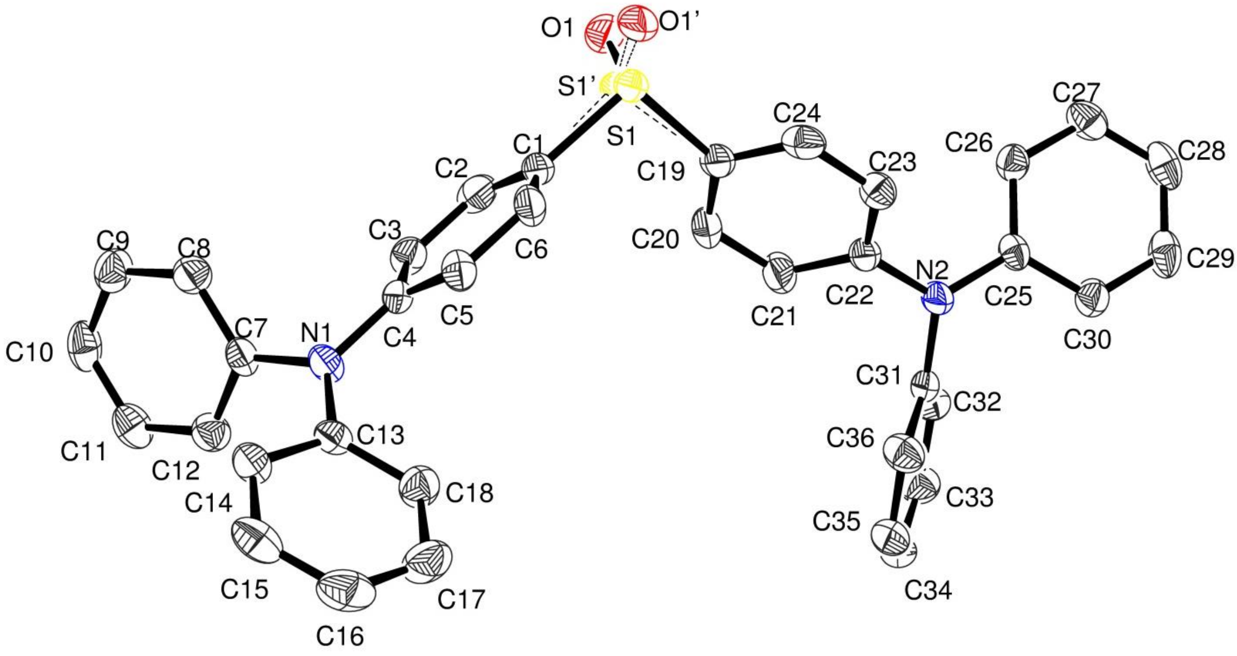

Figure 1 : Asymmetric Unit

Table 1. Crystal data and structure refinement for **4b**.

Empirical formula	C36 H28 N2 O S
Formula weight	536.66
Temperature	193(2) K
Wavelength	0.71073 A
Crystal system, space group	Monoclinic, P 21/n
Unit cell dimensions	a = 12.9683(8) A alpha = 90 deg.
	b = 15.5285(10) A beta = 112.370(3) deg.
	c = 14.6878(11) A gamma = 90 deg

Volume 2735.2(3) A^3

Z, Calculated density 4, 1.303 Mg/m^3

Absorption coefficient 0.151 mm^-1

F(000) 1128

Crystal size 0.240 x 0.100 x 0.080 mm

Theta range for data collection 2.623 to 26.426 deg.

Limiting indices -16<=h<=16, -19<=k<=19,
-18<=l<=18

Reflections collected / unique 61093 / 5591 [R(int) = 0.0579]

Completeness to theta = 25.242 99.5 %

Refinement method Full-matrix least-squares on F^2

Data / restraints / parameters 5591 / 43 / 381

Goodness-of-fit on F^2 1.193

Final R indices [I>2sigma(I)] R1 = 0.0539, wR2 = 0.1076

R indices (all data) R1 = 0.0669, wR2 = 0.1126

Largest diff. peak and hole 0.417 and -0.193 e.A^-3

Table 2. Atomic coordinates (x 10^4) and equivalent isotropic displacement parameters (A^2 x 10^3) for **4b**.
U(eq) is defined as one third of the trace of the orthogonalized Uij tensor.

	x	y	z	U(eq)
C(1)	6941(2)	4257(1)	2922(2)	39(1)
C(19)	8491(2)	5533(2)	3181(2)	41(1)
S(1)	7863(1)	4646(1)	2346(1)	27(1)
O(1)	8782(2)	3995(2)	2684(2)	42(1)
S(1')	8233(2)	4408(1)	2740(2)	27(1)
O(1')	7841(3)	4533(2)	1666(3)	42(1)
C(2)	7145(2)	3680(1)	3692(2)	40(1)
C(3)	6291(2)	3444(1)	3988(2)	31(1)
C(4)	5223(2)	3781(1)	3514(1)	25(1)
C(5)	5027(2)	4348(1)	2727(2)	30(1)
C(6)	5881(2)	4581(1)	2435(2)	38(1)
C(7)	4349(2)	2717(1)	4231(2)	28(1)
C(8)	4466(2)	1980(1)	3749(2)	33(1)
C(9)	4455(2)	1179(1)	4157(2)	39(1)
C(10)	4324(2)	1106(1)	5045(2)	40(1)
C(11)	4204(2)	1839(2)	5528(2)	39(1)
C(12)	4216(2)	2644(1)	5119(2)	34(1)
C(13)	3429(2)	4107(1)	3667(1)	27(1)
C(14)	2355(2)	3770(1)	3350(2)	33(1)
C(15)	1453(2)	4309(2)	3206(2)	42(1)
C(16)	1612(2)	5179(2)	3374(2)	47(1)
C(17)	2679(2)	5514(2)	3691(2)	46(1)
C(18)	3589(2)	4985(1)	3848(2)	35(1)
C(20)	8794(2)	5576(2)	4191(2)	50(1)
C(21)	9307(2)	6307(2)	4706(2)	43(1)
C(22)	9503(2)	7011(1)	4208(2)	28(1)
C(23)	9144(2)	6981(1)	3184(2)	30(1)
C(24)	8657(2)	6238(2)	2678(2)	35(1)
C(25)	10935(2)	8154(1)	4544(1)	27(1)
C(26)	11527(2)	7725(1)	4067(2)	30(1)
C(27)	12376(2)	8138(2)	3885(2)	37(1)
C(28)	12654(2)	8981(2)	4180(2)	41(1)
C(29)	12085(2)	9399(2)	4672(2)	41(1)
C(30)	11228(2)	9002(1)	4849(2)	35(1)
C(31)	9891(2)	7971(1)	5619(2)	27(1)

C(32)	10773(2)	7979(1)	6527(2)	32(1)
C(33)	10580(2)	8192(2)	7360(2)	39(1)
C(34)	9518(2)	8390(1)	7300(2)	40(1)
C(35)	8646(2)	8389(2)	6397(2)	40(1)
C(36)	8826(2)	8182(2)	5554(2)	38(1)
N(1)	4352(1)	3550(1)	3817(1)	30(1)
N(2)	10065(1)	7741(1)	4742(1)	32(1)

Table 3. Bond lengths [A] and angles [deg] for **4b**.

C(1)-C(6)	1.381(3)
C(1)-C(2)	1.386(3)
C(1)-S(1')	1.810(2)
C(1)-S(1)	1.812(2)
C(19)-C(24)	1.383(3)
C(19)-C(20)	1.385(3)
C(19)-S(1)	1.817(2)
C(19)-S(1')	1.849(3)
S(1)-O(1)	1.497(3)
S(1')-O(1')	1.474(4)
C(2)-C(3)	1.385(3)
C(2)-H(2)	0.9500
C(3)-C(4)	1.394(3)
C(3)-H(3)	0.9500
C(4)-C(5)	1.397(3)
C(4)-N(1)	1.409(2)
C(5)-C(6)	1.379(3)
C(5)-H(5)	0.9500
C(6)-H(6)	0.9500
C(7)-C(8)	1.383(3)
C(7)-C(12)	1.384(3)
C(7)-N(1)	1.430(2)
C(8)-C(9)	1.384(3)
C(8)-H(8)	0.9500
C(9)-C(10)	1.382(3)
C(9)-H(9)	0.9500
C(10)-C(11)	1.381(3)
C(10)-H(10)	0.9500
C(11)-C(12)	1.390(3)
C(11)-H(11)	0.9500
C(12)-H(12)	0.9500
C(13)-C(18)	1.389(3)
C(13)-C(14)	1.391(3)
C(13)-N(1)	1.425(2)
C(14)-C(15)	1.388(3)
C(14)-H(14)	0.9500
C(15)-C(16)	1.374(4)
C(15)-H(15)	0.9500
C(16)-C(17)	1.383(4)
C(16)-H(16)	0.9500
C(17)-C(18)	1.383(3)
C(17)-H(17)	0.9500

C(18)-H(18)	0.9500
C(20)-C(21)	1.387(3)
C(20)-H(20)	0.9500
C(21)-C(22)	1.391(3)
C(21)-H(21)	0.9500
C(22)-C(23)	1.395(3)
C(22)-N(2)	1.413(2)
C(23)-C(24)	1.387(3)
C(23)-H(23)	0.9500
C(24)-H(24)	0.9500
C(25)-C(26)	1.390(3)
C(25)-C(30)	1.397(3)
C(25)-N(2)	1.421(2)
C(26)-C(27)	1.385(3)
C(26)-H(26)	0.9500
C(27)-C(28)	1.383(3)
C(27)-H(27)	0.9500
C(28)-C(29)	1.376(3)
C(28)-H(28)	0.9500
C(29)-C(30)	1.380(3)
C(29)-H(29)	0.9500
C(30)-H(30)	0.9500
C(31)-C(36)	1.387(3)
C(31)-C(32)	1.388(3)
C(31)-N(2)	1.435(2)
C(32)-C(33)	1.378(3)
C(32)-H(32)	0.9500
C(33)-C(34)	1.381(3)
C(33)-H(33)	0.9500
C(34)-C(35)	1.377(3)
C(34)-H(34)	0.9500
C(35)-C(36)	1.384(3)
C(35)-H(35)	0.9500
C(36)-H(36)	0.9500
C(6)-C(1)-C(2)	120.11(19)
C(6)-C(1)-S(1')	132.1(2)
C(2)-C(1)-S(1')	107.72(19)
C(6)-C(1)-S(1)	109.85(19)
C(2)-C(1)-S(1)	129.92(19)
C(24)-C(19)-C(20)	119.8(2)
C(24)-C(19)-S(1)	111.10(18)
C(20)-C(19)-S(1)	129.13(19)
C(24)-C(19)-S(1')	127.22(18)
C(20)-C(19)-S(1')	111.1(2)

O(1)-S(1)-C(1)	101.70(16)
O(1)-S(1)-C(19)	101.04(16)
C(1)-S(1)-C(19)	98.27(11)
O(1')-S(1')-C(1)	102.3(2)
O(1')-S(1')-C(19)	101.2(2)
C(1)-S(1')-C(19)	97.18(11)
C(3)-C(2)-C(1)	120.0(2)
C(3)-C(2)-H(2)	120.0
C(1)-C(2)-H(2)	120.0
C(2)-C(3)-C(4)	120.3(2)
C(2)-C(3)-H(3)	119.8
C(4)-C(3)-H(3)	119.8
C(3)-C(4)-C(5)	118.89(18)
C(3)-C(4)-N(1)	120.66(18)
C(5)-C(4)-N(1)	120.46(17)
C(6)-C(5)-C(4)	120.6(2)
C(6)-C(5)-H(5)	119.7
C(4)-C(5)-H(5)	119.7
C(5)-C(6)-C(1)	120.1(2)
C(5)-C(6)-H(6)	119.9
C(1)-C(6)-H(6)	119.9
C(8)-C(7)-C(12)	119.49(19)
C(8)-C(7)-N(1)	120.80(18)
C(12)-C(7)-N(1)	119.70(19)
C(7)-C(8)-C(9)	120.0(2)
C(7)-C(8)-H(8)	120.0
C(9)-C(8)-H(8)	120.0
C(10)-C(9)-C(8)	120.5(2)
C(10)-C(9)-H(9)	119.8
C(8)-C(9)-H(9)	119.8
C(11)-C(10)-C(9)	119.8(2)
C(11)-C(10)-H(10)	120.1
C(9)-C(10)-H(10)	120.1
C(10)-C(11)-C(12)	119.8(2)
C(10)-C(11)-H(11)	120.1
C(12)-C(11)-H(11)	120.1
C(7)-C(12)-C(11)	120.4(2)
C(7)-C(12)-H(12)	119.8
C(11)-C(12)-H(12)	119.8
C(18)-C(13)-C(14)	119.36(19)
C(18)-C(13)-N(1)	120.70(18)
C(14)-C(13)-N(1)	119.93(18)
C(15)-C(14)-C(13)	120.2(2)
C(15)-C(14)-H(14)	119.9
C(13)-C(14)-H(14)	119.9

C(16)-C(15)-C(14)	120.3(2)
C(16)-C(15)-H(15)	119.8
C(14)-C(15)-H(15)	119.8
C(15)-C(16)-C(17)	119.5(2)
C(15)-C(16)-H(16)	120.2
C(17)-C(16)-H(16)	120.2
C(18)-C(17)-C(16)	120.9(2)
C(18)-C(17)-H(17)	119.5
C(16)-C(17)-H(17)	119.5
C(17)-C(18)-C(13)	119.7(2)
C(17)-C(18)-H(18)	120.2
C(13)-C(18)-H(18)	120.2
C(19)-C(20)-C(21)	120.4(2)
C(19)-C(20)-H(20)	119.8
C(21)-C(20)-H(20)	119.8
C(20)-C(21)-C(22)	120.1(2)
C(20)-C(21)-H(21)	119.9
C(22)-C(21)-H(21)	119.9
C(21)-C(22)-C(23)	119.17(19)
C(21)-C(22)-N(2)	119.82(18)
C(23)-C(22)-N(2)	121.01(18)
C(24)-C(23)-C(22)	120.2(2)
C(24)-C(23)-H(23)	119.9
C(22)-C(23)-H(23)	119.9
C(19)-C(24)-C(23)	120.2(2)
C(19)-C(24)-H(24)	119.9
C(23)-C(24)-H(24)	119.9
C(26)-C(25)-C(30)	118.56(18)
C(26)-C(25)-N(2)	121.46(18)
C(30)-C(25)-N(2)	119.97(18)
C(27)-C(26)-C(25)	120.5(2)
C(27)-C(26)-H(26)	119.8
C(25)-C(26)-H(26)	119.8
C(28)-C(27)-C(26)	120.6(2)
C(28)-C(27)-H(27)	119.7
C(26)-C(27)-H(27)	119.7
C(29)-C(28)-C(27)	118.9(2)
C(29)-C(28)-H(28)	120.5
C(27)-C(28)-H(28)	120.5
C(28)-C(29)-C(30)	121.3(2)
C(28)-C(29)-H(29)	119.4
C(30)-C(29)-H(29)	119.4
C(29)-C(30)-C(25)	120.1(2)
C(29)-C(30)-H(30)	119.9
C(25)-C(30)-H(30)	119.9

C(36)-C(31)-C(32)	119.89(19)
C(36)-C(31)-N(2)	119.32(18)
C(32)-C(31)-N(2)	120.79(18)
C(33)-C(32)-C(31)	119.6(2)
C(33)-C(32)-H(32)	120.2
C(31)-C(32)-H(32)	120.2
C(32)-C(33)-C(34)	120.7(2)
C(32)-C(33)-H(33)	119.6
C(34)-C(33)-H(33)	119.6
C(35)-C(34)-C(33)	119.7(2)
C(35)-C(34)-H(34)	120.2
C(33)-C(34)-H(34)	120.2
C(34)-C(35)-C(36)	120.4(2)
C(34)-C(35)-H(35)	119.8
C(36)-C(35)-H(35)	119.8
C(35)-C(36)-C(31)	119.8(2)
C(35)-C(36)-H(36)	120.1
C(31)-C(36)-H(36)	120.1
C(4)-N(1)-C(13)	121.61(16)
C(4)-N(1)-C(7)	119.77(16)
C(13)-N(1)-C(7)	118.50(16)
C(22)-N(2)-C(25)	122.00(16)
C(22)-N(2)-C(31)	118.29(16)
C(25)-N(2)-C(31)	118.71(16)

Symmetry transformations used to generate equivalent atoms:

Table 4. Anisotropic displacement parameters (A^2 x 10^3) for **4b**.
The anisotropic displacement factor exponent takes the form:
-2 pi^2 [h^2 a*^2 U11 + ... + 2 h k a* b* U12]

	U11	U22	U33	U23	U13	U12
C(1)	41(1)	36(1)	55(2)	-23(1)	35(1)	-21(1)
C(19)	38(1)	42(1)	56(2)	-24(1)	32(1)	-22(1)
S(1)	27(1)	30(1)	27(1)	-4(1)	13(1)	-4(1)
O(1)	35(2)	35(2)	66(2)	-10(1)	29(2)	-2(1)
S(1')	25(1)	25(1)	38(1)	-8(1)	19(1)	-5(1)
O(1')	53(2)	47(2)	36(2)	-14(2)	28(2)	-17(2)
C(2)	24(1)	36(1)	61(2)	-19(1)	17(1)	-5(1)
C(3)	27(1)	28(1)	36(1)	-2(1)	10(1)	1(1)
C(4)	24(1)	23(1)	28(1)	-4(1)	11(1)	-4(1)
C(5)	30(1)	30(1)	29(1)	1(1)	10(1)	-5(1)
C(6)	50(1)	35(1)	36(1)	-7(1)	24(1)	-20(1)
C(7)	23(1)	26(1)	37(1)	7(1)	14(1)	1(1)
C(8)	33(1)	31(1)	38(1)	3(1)	16(1)	3(1)
C(9)	39(1)	26(1)	51(1)	2(1)	17(1)	2(1)
C(10)	32(1)	28(1)	54(1)	15(1)	12(1)	0(1)
C(11)	36(1)	44(1)	36(1)	13(1)	14(1)	0(1)
C(12)	37(1)	34(1)	35(1)	3(1)	16(1)	1(1)
C(13)	27(1)	29(1)	27(1)	6(1)	14(1)	5(1)
C(14)	31(1)	34(1)	37(1)	4(1)	16(1)	-1(1)
C(15)	28(1)	59(2)	42(1)	11(1)	17(1)	6(1)
C(16)	43(1)	53(2)	52(2)	10(1)	25(1)	22(1)
C(17)	53(2)	32(1)	55(2)	1(1)	24(1)	12(1)
C(18)	34(1)	31(1)	41(1)	1(1)	14(1)	2(1)
C(20)	74(2)	37(1)	57(2)	-12(1)	45(1)	-26(1)
C(21)	60(2)	40(1)	34(1)	-5(1)	25(1)	-19(1)
C(22)	25(1)	29(1)	31(1)	-5(1)	13(1)	-6(1)
C(23)	25(1)	34(1)	30(1)	0(1)	10(1)	-2(1)
C(24)	23(1)	50(1)	32(1)	-13(1)	11(1)	-6(1)
C(25)	25(1)	30(1)	25(1)	2(1)	8(1)	-6(1)
C(26)	29(1)	26(1)	35(1)	3(1)	12(1)	1(1)
C(27)	27(1)	47(1)	40(1)	8(1)	15(1)	5(1)
C(28)	28(1)	51(1)	39(1)	9(1)	9(1)	-12(1)
C(29)	44(1)	38(1)	34(1)	-1(1)	8(1)	-20(1)
C(30)	42(1)	31(1)	33(1)	-3(1)	15(1)	-7(1)
C(31)	33(1)	22(1)	31(1)	-4(1)	17(1)	-6(1)
C(32)	33(1)	30(1)	36(1)	-1(1)	15(1)	2(1)

C(33)	44(1)	41(1)	31(1)	-3(1)	13(1)	0(1)
C(34)	53(1)	34(1)	43(1)	-6(1)	31(1)	-3(1)
C(35)	37(1)	38(1)	55(2)	-1(1)	28(1)	0(1)
C(36)	31(1)	46(1)	37(1)	-1(1)	14(1)	-3(1)
N(1)	28(1)	27(1)	41(1)	10(1)	20(1)	5(1)
N(2)	37(1)	32(1)	33(1)	-9(1)	20(1)	-13(1)

Molecule **4s**:

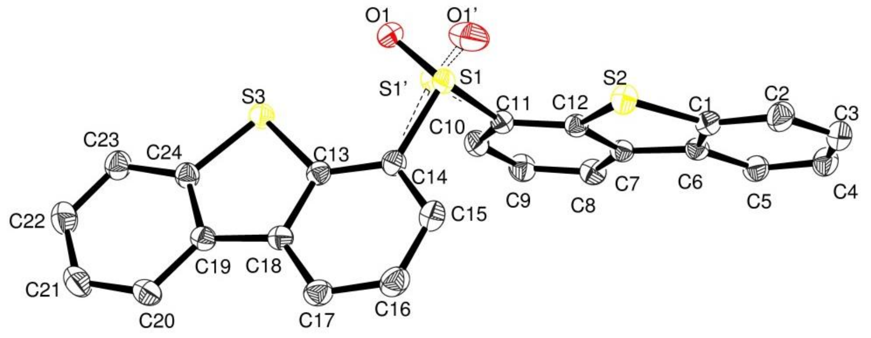

Figure 1 : Asymmetric Unit

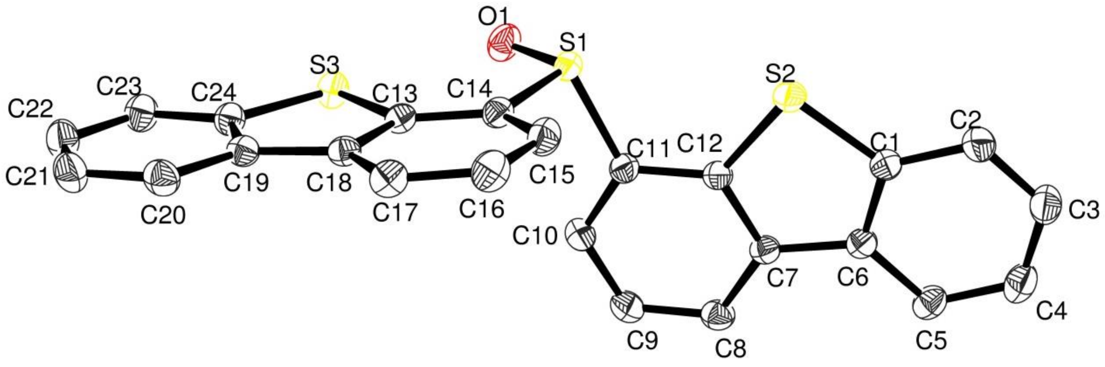

Figure 2 : Compound

Table 1. Crystal data and structure refinement for **4s**.

Empirical formula	C24 H14 O S3
Formula weight	414.53
Temperature	193(2) K
Wavelength	0.71073 A

Crystal system, space group Monoclinic, P 21/c

Unit cell dimensions a = 14.0948(8) A alpha = 90 deg.
b = 15.4204(9) A beta = 100.915(2) deg.
c = 8.6011(5) A gamma = 90 deg.

Volume 1835.61(18) A^3

Z, Calculated density 4, 1.500 Mg/m^3

Absorption coefficient 0.417 mm^-1

F(000) 856

Crystal size 0.200 x 0.080 x 0.060 mm

Theta range for data collection 2.750 to 34.402 deg.

Limiting indices -22<=h<=22, -24<=k<=24,
-13<=l<=13

Reflections collected / unique 68657 / 7700 [R(int) = 0.0396]

Completeness to theta = 25.242 99.6 %

Refinement method Full-matrix least-squares on F^2

Data / restraints / parameters 7700 / 37 / 273

Goodness-of-fit on F^2 1.055

Final R indices [I>2sigma(I)] R1 = 0.0349, wR2 = 0.0915

R indices (all data) R1 = 0.0450, wR2 = 0.0977

Largest diff. peak and hole 0.495 and -0.501 e.A^-3

Table 2. Atomic coordinates (x 10^4) and equivalent isotropic displacement parameters (A^2 x 10^3) for **4s**.
U(eq) is defined as one third of the trace of the orthogonalized Uij tensor.

	x	y	z	U(eq)
C(1)	847(1)	3889(1)	5699(1)	23(1)
C(2)	370(1)	3159(1)	6126(2)	31(1)
C(3)	10(1)	3187(1)	7511(2)	34(1)
C(4)	141(1)	3917(1)	8485(2)	31(1)
C(5)	625(1)	4638(1)	8077(1)	26(1)
C(6)	976(1)	4632(1)	6659(1)	21(1)
C(7)	1489(1)	5311(1)	5990(1)	20(1)
C(8)	1759(1)	6136(1)	6588(1)	25(1)
C(9)	2232(1)	6698(1)	5728(1)	28(1)
C(10)	2446(1)	6447(1)	4274(1)	26(1)
C(11)	2191(1)	5625(1)	3673(1)	22(1)
C(14)	3658(1)	5160(1)	2196(1)	22(1)
S(1)	2370(1)	5293(1)	1753(1)	23(1)
O(1)	2170(1)	6061(1)	676(1)	30(1)
S(1')	2416(2)	5492(4)	1705(1)	34(2)
O(1')	1853(2)	4717(7)	1006(9)	47(3)
C(12)	1713(1)	5056(1)	4525(1)	20(1)
C(13)	4283(1)	5699(1)	1556(1)	20(1)
C(15)	4017(1)	4444(1)	3101(1)	27(1)
C(16)	5005(1)	4271(1)	3411(2)	29(1)
C(17)	5638(1)	4800(1)	2803(1)	25(1)
C(18)	5284(1)	5514(1)	1865(1)	20(1)
C(19)	5825(1)	6122(1)	1089(1)	21(1)
C(20)	6818(1)	6142(1)	1096(1)	26(1)
C(21)	7190(1)	6790(1)	271(2)	32(1)
C(22)	6586(1)	7411(1)	-581(2)	32(1)
C(23)	5597(1)	7398(1)	-625(1)	27(1)
C(24)	5223(1)	6751(1)	226(1)	22(1)
S(2)	1327(1)	4002(1)	3977(1)	25(1)
S(3)	4006(1)	6611(1)	335(1)	24(1)

Table 3. Bond lengths [A] and angles [deg] for **4s**.

C(1)-C(2)	1.3956(16)
C(1)-C(6)	1.4040(15)
C(1)-S(2)	1.7504(11)
C(2)-C(3)	1.3808(18)
C(2)-H(2)	0.9500
C(3)-C(4)	1.395(2)
C(3)-H(3)	0.9500
C(4)-C(5)	1.3847(17)
C(4)-H(4)	0.9500
C(5)-C(6)	1.4002(15)
C(5)-H(5)	0.9500
C(6)-C(7)	1.4511(14)
C(7)-C(8)	1.3987(15)
C(7)-C(12)	1.4113(14)
C(8)-C(9)	1.3889(17)
C(8)-H(8)	0.9500
C(9)-C(10)	1.3961(17)
C(9)-H(9)	0.9500
C(10)-C(11)	1.3892(16)
C(10)-H(10)	0.9500
C(11)-C(12)	1.3956(14)
C(11)-S(1')	1.7923(14)
C(11)-S(1)	1.7926(11)
C(14)-C(15)	1.3900(16)
C(14)-C(13)	1.3975(14)
C(14)-S(1)	1.7944(11)
C(14)-S(1')	1.7947(14)
S(1)-O(1)	1.4967(11)
S(1')-O(1')	1.4971(15)
C(12)-S(2)	1.7498(10)
C(13)-C(18)	1.4153(14)
C(13)-S(3)	1.7536(10)
C(15)-C(16)	1.3934(17)
C(15)-H(15)	0.9500
C(16)-C(17)	1.3828(17)
C(16)-H(16)	0.9500
C(17)-C(18)	1.3995(15)
C(17)-H(17)	0.9500
C(18)-C(19)	1.4491(15)
C(19)-C(20)	1.3983(15)
C(19)-C(24)	1.4047(14)
C(20)-C(21)	1.3847(17)

C(20)-H(20)	0.9500
C(21)-C(22)	1.3945(19)
C(21)-H(21)	0.9500
C(22)-C(23)	1.3876(17)
C(22)-H(22)	0.9500
C(23)-C(24)	1.3972(15)
C(23)-H(23)	0.9500
C(24)-S(3)	1.7501(11)

C(2)-C(1)-C(6)	121.31(10)
C(2)-C(1)-S(2)	125.87(9)
C(6)-C(1)-S(2)	112.82(8)
C(3)-C(2)-C(1)	118.37(12)
C(3)-C(2)-H(2)	120.8
C(1)-C(2)-H(2)	120.8
C(2)-C(3)-C(4)	121.03(11)
C(2)-C(3)-H(3)	119.5
C(4)-C(3)-H(3)	119.5
C(5)-C(4)-C(3)	120.75(11)
C(5)-C(4)-H(4)	119.6
C(3)-C(4)-H(4)	119.6
C(4)-C(5)-C(6)	119.22(11)
C(4)-C(5)-H(5)	120.4
C(6)-C(5)-H(5)	120.4
C(5)-C(6)-C(1)	119.29(10)
C(5)-C(6)-C(7)	128.71(10)
C(1)-C(6)-C(7)	112.00(9)
C(8)-C(7)-C(12)	119.38(10)
C(8)-C(7)-C(6)	129.15(9)
C(12)-C(7)-C(6)	111.47(9)
C(9)-C(8)-C(7)	119.77(10)
C(9)-C(8)-H(8)	120.1
C(7)-C(8)-H(8)	120.1
C(8)-C(9)-C(10)	120.70(10)
C(8)-C(9)-H(9)	119.7
C(10)-C(9)-H(9)	119.7
C(11)-C(10)-C(9)	120.13(10)
C(11)-C(10)-H(10)	119.9
C(9)-C(10)-H(10)	119.9
C(10)-C(11)-C(12)	119.67(10)
C(10)-C(11)-S(1')	112.1(2)
C(12)-C(11)-S(1')	127.8(2)
C(10)-C(11)-S(1)	122.12(8)
C(12)-C(11)-S(1)	118.03(8)
C(15)-C(14)-C(13)	120.20(10)

C(15)-C(14)-S(1)	117.13(8)
C(13)-C(14)-S(1)	122.43(8)
C(15)-C(14)-S(1')	126.4(2)
C(13)-C(14)-S(1')	113.4(2)
O(1)-S(1)-C(11)	107.51(6)
O(1)-S(1)-C(14)	106.64(6)
C(11)-S(1)-C(14)	98.64(5)
O(1')-S(1')-C(11)	107.21(15)
O(1')-S(1')-C(14)	106.63(15)
C(11)-S(1')-C(14)	98.64(7)
C(11)-C(12)-C(7)	120.34(9)
C(11)-C(12)-S(2)	126.82(8)
C(7)-C(12)-S(2)	112.83(7)
C(14)-C(13)-C(18)	119.11(9)
C(14)-C(13)-S(3)	128.81(8)
C(18)-C(13)-S(3)	112.07(8)
C(14)-C(15)-C(16)	120.27(10)
C(14)-C(15)-H(15)	119.9
C(16)-C(15)-H(15)	119.9
C(17)-C(16)-C(15)	120.54(10)
C(17)-C(16)-H(16)	119.7
C(15)-C(16)-H(16)	119.7
C(16)-C(17)-C(18)	119.77(10)
C(16)-C(17)-H(17)	120.1
C(18)-C(17)-H(17)	120.1
C(17)-C(18)-C(13)	120.08(10)
C(17)-C(18)-C(19)	127.84(10)
C(13)-C(18)-C(19)	112.07(9)
C(20)-C(19)-C(24)	119.37(10)
C(20)-C(19)-C(18)	128.75(10)
C(24)-C(19)-C(18)	111.87(9)
C(21)-C(20)-C(19)	119.34(11)
C(21)-C(20)-H(20)	120.3
C(19)-C(20)-H(20)	120.3
C(20)-C(21)-C(22)	120.77(11)
C(20)-C(21)-H(21)	119.6
C(22)-C(21)-H(21)	119.6
C(23)-C(22)-C(21)	120.99(11)
C(23)-C(22)-H(22)	119.5
C(21)-C(22)-H(22)	119.5
C(22)-C(23)-C(24)	118.18(11)
C(22)-C(23)-H(23)	120.9
C(24)-C(23)-H(23)	120.9
C(23)-C(24)-C(19)	121.34(10)
C(23)-C(24)-S(3)	125.91(9)

C(19)-C(24)-S(3)	112.74(8)
C(12)-S(2)-C(1)	90.87(5)
C(24)-S(3)-C(13)	91.25(5)

Symmetry transformations used to generate equivalent atoms:

Table 4. Anisotropic displacement parameters (A^2 x 10^3) for **4s**.
The anisotropic displacement factor exponent takes the form:
-2 pi^2 [h^2 a*^2 U11 + ... + 2 h k a* b* U12]

	U11	U22	U33	U23	U13	U12
C(1)	23(1)	24(1)	21(1)	1(1)	3(1)	-1(1)
C(2)	37(1)	28(1)	27(1)	3(1)	2(1)	-9(1)
C(3)	32(1)	39(1)	30(1)	9(1)	4(1)	-9(1)
C(4)	28(1)	40(1)	28(1)	8(1)	10(1)	2(1)
C(5)	24(1)	32(1)	23(1)	2(1)	7(1)	4(1)
C(6)	18(1)	24(1)	20(1)	1(1)	3(1)	2(1)
C(7)	18(1)	22(1)	19(1)	-1(1)	3(1)	1(1)
C(8)	26(1)	25(1)	24(1)	-4(1)	5(1)	0(1)
C(9)	30(1)	24(1)	29(1)	-4(1)	5(1)	-5(1)
C(10)	26(1)	26(1)	27(1)	0(1)	4(1)	-6(1)
C(11)	19(1)	26(1)	19(1)	0(1)	4(1)	-1(1)
C(14)	22(1)	25(1)	19(1)	-1(1)	5(1)	0(1)
S(1)	21(1)	31(1)	18(1)	-1(1)	5(1)	-4(1)
O(1)	24(1)	43(1)	24(1)	9(1)	3(1)	1(1)
S(1')	18(2)	57(4)	27(2)	13(2)	4(2)	-7(2)
O(1')	33(5)	57(6)	46(5)	1(5)	-7(5)	-5(5)
C(12)	19(1)	21(1)	19(1)	-1(1)	3(1)	0(1)
C(13)	21(1)	21(1)	18(1)	-1(1)	4(1)	2(1)
C(15)	31(1)	26(1)	26(1)	4(1)	8(1)	-1(1)
C(16)	33(1)	26(1)	29(1)	7(1)	6(1)	5(1)
C(17)	24(1)	26(1)	26(1)	3(1)	3(1)	5(1)
C(18)	20(1)	21(1)	19(1)	-2(1)	3(1)	2(1)
C(19)	20(1)	21(1)	21(1)	-2(1)	4(1)	1(1)
C(20)	20(1)	30(1)	29(1)	-2(1)	3(1)	1(1)
C(21)	22(1)	37(1)	37(1)	-3(1)	8(1)	-6(1)
C(22)	32(1)	31(1)	35(1)	1(1)	11(1)	-7(1)
C(23)	30(1)	25(1)	29(1)	3(1)	8(1)	0(1)
C(24)	21(1)	22(1)	23(1)	0(1)	6(1)	1(1)
S(2)	31(1)	22(1)	22(1)	-4(1)	7(1)	-3(1)
S(3)	20(1)	25(1)	26(1)	6(1)	6(1)	5(1)
